# Dynamic measurements of geographical accessibility considering traffic congestion using open data: a cross-sectional assessment for haemodialysis services in Cali, Colombia

**DOI:** 10.1016/j.lana.2024.100752

**Published:** 2024-05-03

**Authors:** Luis Gabriel Cuervo, Carmen Juliana Villamizar, Lyda Osorio, María Beatriz Ospina, Diana E. Cuervo, Daniel Cuervo, María O. Bula, Pablo Zapata, Nancy J. Owens, Janet Hatcher-Roberts, Edith Alejandra Martín, Felipe Piquero, Luis Fernando Pinilla, Eliana Martínez-Herrera, Ciro Jaramillo, The AMORE Project Collaboration

**Affiliations:** a Universitat Autònoma de Barcelona, Barcelona, Spain; b Johns Hopkins Bloomberg School of Public Health, Baltimore, MD, USA; c School of Public Health, Universidad del Valle, Cali, Colombia; d Faculty of Health Sciences, Queen's University, Kingston, ON, Canada; e National Disability Board of Colombia, Bogotá, Colombia; f IQuartil SAS, Bogotá, Colombia; g Independent Researcher, Bogotá, Colombia; h Independent Content and Communications Consultant, Fairfax, VA, USA; i School of Epidemiology and Public Health in the Faculty of Medicine, and Bruyère Research Institute, University of Ottawa, Ottawa, ON, Canada; j Colombian Association of Transplanted Athletes, Bogota, Colombia; k Patient Advocate and Author of an Autopathography, Bogotá, Colombia; l Universidad de la Sabana, Campus del Puente del Común, Chía, Cundinamarca, Colombia; m National Faculty of Public Health, Universidad de Antioquia, Medellín, Colombia; n School of Civil and Geomatic Engineering of the Universidad del Valle, Cali, Colombia

**Keywords:** Accessibility indicators, Dynamic accessibility assessments, Health services accessibility, People-centred focus, Multistakeholder collaboration, Hemodialysis, Health services research, Geographic accessibility, Health services planning, Urban planning innovation, Intersectoral interactions, Knowledge translation and dissemination, Research for health

## Abstract

**Background:**

Many cities with traffic congestion lack accessibility assessments accounting for traffic congestion and equity considerations but have disaggregated georeferenced municipal-level open data on health services, populations, and travel times big data. We convened a multistakeholder intersectoral collaborative group that developed a digital, web-based platform integrating open and big data to derive dynamic spatial–temporal accessibility measurements (DSTAM) for haemodialysis services. We worked with stakeholders and data scientists and considered people's places of residence, service locations, and travel time to the service with the shortest travel time. Additionally, we predicted the impacts of strategically introducing haemodialysis services where they optimise accessibility.

**Methods:**

Cross-sectional analyses of DSTAM, accounting for traffic congestion, were conducted using a web-based platform. This platform integrated traffic analysis zones, public census and health services datasets, and Google Distance Matrix API travel-time data. Predictive and prescriptive analytics identified optimal locations for new haemodialysis services and estimated improvements. Primary outcomes included the percentage of residents within a 20-min car drive of a haemodialysis service during peak and free-flow traffic congestion. Secondary outcomes focused on optimal locations to maximise accessibility with new services and potential improvements. Findings were disaggregated by sociodemographic characteristics, providing an equity perspective. The study in Cali, Colombia, used geographic and disaggregated sociodemographic data from the adjusted 2018 Colombian census. Predicted travel times were obtained for two weeks in 2020.

**Findings:**

There were substantial traffic variations. Congestion reduced accessibility, especially among marginalised groups. For 6–12 July, free-flow and peak-traffic accessibility rates were 95.2% and 45.0%, respectively. For 23–29 November, free-flow and peak traffic accessibility rates were 89.1% and 69.7%. The locations where new services would optimise accessibility had slight variation and would notably enhance accessibility and health equity.

**Interpretation:**

Establishing haemodialysis services in targeted areas has significant potential benefits. By increasing accessibility, it would enhance urban health and equity.

**Funding:**

No external or institutional funding was received.

**Research in context**

**Evidence before this study**

Haemodialysis is a costly and critical ambulatory treatment that patients with conditions like terminal renal failure require several times weekly. These patients and their families face direct and indirect commuting costs; longer travel times, which can be determined by heavy traffic congestion, are detrimental to the quality of care and strain their finances.

Understanding how different sociodemographic groups are affected by commuting times to haemodialysis facilities is relevant when developing urban and health services plans.

Between February 1st and June 30 2020, we searched the Web of Science Core Collection and Google Scholar, plus specialised databases including (PubMed, Lilacs, Epistemonikos, EvidenceAid, and McMasters Health Forum's Health Systems Evidence). We searched for [accessibility OR spatial analysis OR dynamic accessibility OR geographic accessibility OR spatial–temporal accessibility OR spatial analysis OR travel time OR traffic congestion] AND [health services OR hemodialysis OR haemodialysis OR radiotherapy OR urban health]. The identified and subsequent references are kept in the Collaborative AMORE Project's Zotero collection https://www.zotero.org/groups/2499307/mapas_dinmicos_y_acceso_equitativo_en_salud with dynamic accessibility references are filed in a subcollection. Our focused literature search from February to June 2020 and an independent review in 2021 showed that current assessments seldom account for traffic fluctuations, do not consider the needs of diverse stakeholders, and do not provide an equity perspective. We cite the few existing dynamic accessibility assessments focused on health services. These cater to specific technical specialists. They recommend policymakers use their results but have yet to include their perspectives in the development of the study.

The importance of monitoring and measuring the alignment of urban and health agendas through participatory processes has garnered widespread recognition. It necessitates data and indicators linking traffic congestion, equity, and accessibility, that are accessible to stakehoders. Policy and technical documents, forums, and expert groups have raised and discussed these needs.

Significant discussions have occurred at events such as the launch of The Lancet Series on Urban Design, Transport, and Health, along with publications like “Health and Urbanism,” cited in this article. This study's protocoland its application have undergone thorough examination across various technical, academic, and collaborative setting, engaging policymakers, researchers, and experts spanning diverse disciplines, including health systems research, smart cities, public health, urban health, territorial and health services planning, healthcare quality and safety, innovations for health, and public policy and governance.

Moreover, the methodology has been showcased in innovations and data sciences competitions (https://padlet.com/Proyecto_AMORE_Project/products, shared and discussed with expert groups pursuing similar or complementary objectives. Presentations at urban health and learning networks, urban observatories, and within research communities such as UKRI-GCRF GREAT, OnTIME Consortium, CEDEUS, and IntalINC-LAC.

Thus, we concluded that simplified, efficient, and dynamic accessibility measurements are needed to guide improvements to haemodialysis and other health services in heavily congested cities. Our research aims to assess haemodialysis accessibility accounting for traffic fluctuations. The study included policymakers, data scientists, and other stakeholders at every stage, from conceptualisation to planning, implementation, and reporting. The results from the study can contribute an equity perspective to the alignment of health and urban planning.

**Added value of this study**

This study proposes a novel approach tested in Cali, Colombia. It has significant practical implications for urban and health services planning. It exposes the intricate connections between service distribution, population, and health equity. It leverages available open data and millions of travel time measurements. Descriptive statistics, simple graphs, and cartography illustrate haemodialysis accessibility across various traffic congestion levels and different time thresholds using disaggregated housing and population data. The study provides an interactive platform that allows parameters and assumptions to be adjusted for varying traffic congestion levels. This offers tangible insights into the territorial distribution of haemodialysis services and their link to health equity. Most importantly, it points to the sectors where new services can optimise accessibility, predicting their impact and providing a clear roadmap for future planning, monitoring, and refinement.

**Implications of all the available evidence**

This study paves the way for future research -including implementation research, knowledge translation, and a shared narrative on accessibility to haemodialysis and other health services in cities with traffic congestion. Subsequent studies could explore adopting and utilising these data in shaping urban and health services planning, as well as the development of new accessibility and quality metrics for cities with traffic congestion. Furthermore, integrating these metrics with real-time service availability, insurance coverage, or other services needed for comprehensive treatment could be a promising avenue for further investigation. Developing this new research line and applying our findings could significantly enhance the accessibility and quality of health services in urban areas.

## Introduction

Accessibility to essential health services advances sustainable development goals (SDGs), improves health and well-being (SDG 3), reduces inequalities (SDG 10) and makes cities and communities more sustainable (SDG 11).^1^ Accessibility measures how easily and quickly people can reach a particular location, such as a health service facility. It is a dynamic attribute because it can change over time due to factors such as changes in transportation infrastructure, traffic patterns, and population density; these changes usually happen over months or years, and therefore, it is helpful to monitor geographical accessibility to health services and its impact on equity.

In cities with notable traffic congestion, accessibility fluctuates throughout the day and week due to cyclical variations in traffic congestion, and tools are needed to measure these fluctuations and find ways of reducing the impact of traffic congestion on health equity. Measuring accessibility opportunity in terms of the travel times helps us understand how long people need to travel to access healthcare and can be used to identify areas with limited accessibility to healthcare and point at solutions.^2^^,^^3^ United Nations agencies have emphasised the importance of integrating health into urban policies through participatory processes and ensuring health equity. The overarching goals involve facilitating access to essential services and ensuring no one is left behind.^4^^,^^5^

Colombia aims to offer universal health coverage through a two-tier health services system and special regimes that cover insured and uninsured populations.^6^^,^^7^ Nonetheless, travel times and their associated direct and indirect costs represent access barriers to care. In 2019, the Colombian Constitutional Court ruled that the national health insurance program must cover transportation costs, but Colombian insurers still need to comply fully with this order.^8^

Haemodialysis is an expensive, life-saving treatment usually administered three times a week for about 4 h. Regular use and adherence usually improve the quality of life of people with end-stage renal disease (ESRD).^9^, ^10^, ^11^ Roughly three of every four people requiring kidney replacement therapy in Colombia are in haemodialysis. There are 516 patients per million population (PMP) in haemodialysis and 103 new PMPs yearly.^12^ The direct and indirect costs of frequent commutes to haemodialysis can impoverish patients and their families. Home haemodialysis is not widely available in Colombia,^13^ and travel time to haemodialysis might be associated with higher mortality rates and lower health-related quality of life.^14^^,^^15^ People in outlying areas of Cali have described the challenges they face seeking haemodialysis in video testimonies.^16^

A family member usually accompanies the patient to haemodialysis sessions, often missing work or the chance to seek a new job or a promotion.^17^^,^^18^ Thus the financial burden of haemodialysis on patients and family finances becomes a significant public health issue given the inextricable links between income and population health.^16^ Haemodialysis causes changes that can impair physical capacity and mobility, and patients need endurance training to counter these changes, but that training is yet to become a standard practice.^19^^,^^20^ Such mobility issues add to the challenges of long journeys.

DSTAM account for traffic congestion delays. Its increasing availability allows researchers to measure the impact of traffic congestion on accessibility and health equity.^2^^,^^21^, ^22^, ^23^, ^24^, ^25^, ^26^, ^27^ DSTAM can inform health services and related land-use planning from an equity perspective. In this project, DSTAM can rely on simple metrics like time-to-destination to monitor and address accessibility, health equity, and social justice factors.^2^ Unlike most accessibility studies, which use sophisticated methods requiring specialised expertise, the AMORE Project follows knowledge translation good practices and simplified metrics and methods, allowing for a broader engagement.^2^^,^^3^^,^^28^^,^^29^ It takes a holistic approach to haemodialysis services planning by engaging a broad range of stakeholders with a person-centred strategy. This approach aims to translate integrated knowledge and deliver information that stakeholders can communicate to their peers and the public.^3^^,^^4^^,^^30^, ^31^, ^32^, ^33^, ^34^ Those stakeholders represent different levels of governance and bring insights relevant to haemodialysis demand (public health, demography, patients and advocates), offer (providers, patients, health systems and services planners) and the mobility to complete the journey between the place where the demand is initiated (residence) and the destination (haemodialysis service), adding insights about land-use, territorial analyses, smart cities, mobility, infrastructure. This brings to the process of haemodialysis services planning the actors and decision-makers representing challenges, tensions and opportunities.

This study is part of the AMORE Project, a proof-of-concept evaluating new approaches to place-based dynamic spatial–temporal accessibility measurements (DSTAM) for health services. It integrates publicly available data to deliver fast and affordable granular assessments of DSTAM for an entire city with an equity perspective.^2^^,^^3^^,^^35^^,^^36^ An earlier report assessed accessibility in urban Cali to tertiary care emergency services, which are essential to all residents.^37^ This study assesses accessibility to haemodialysis services. It identifies the approximate 1–2 location(s) where new services would place most of the urban population of Cali within 20 min of a haemodialysis service by car, and the population in the catchment area of those locations. This study thus provides a proxy measurement and new data to inform health services planning and a strategy for reducing inequities in accessibility.^3^

We sought to explore: 1) What potential accessibility to haemodialysis did urban Cali dwellers have in 2020, depending on their sociodemographic characteristics? 2) Did accessibility vary according to socioeconomic status in urban Cali? 3) What improvements would result from strategically adding new haemodialysis services in 1–2 new neighbourhoods in Cali? 4) Changes in accessibility, by comparing two weeks of 2020.

This research is aligned with urban health research priorities.^38^

## Methods

### Study population and setting

**Fig. 1:**
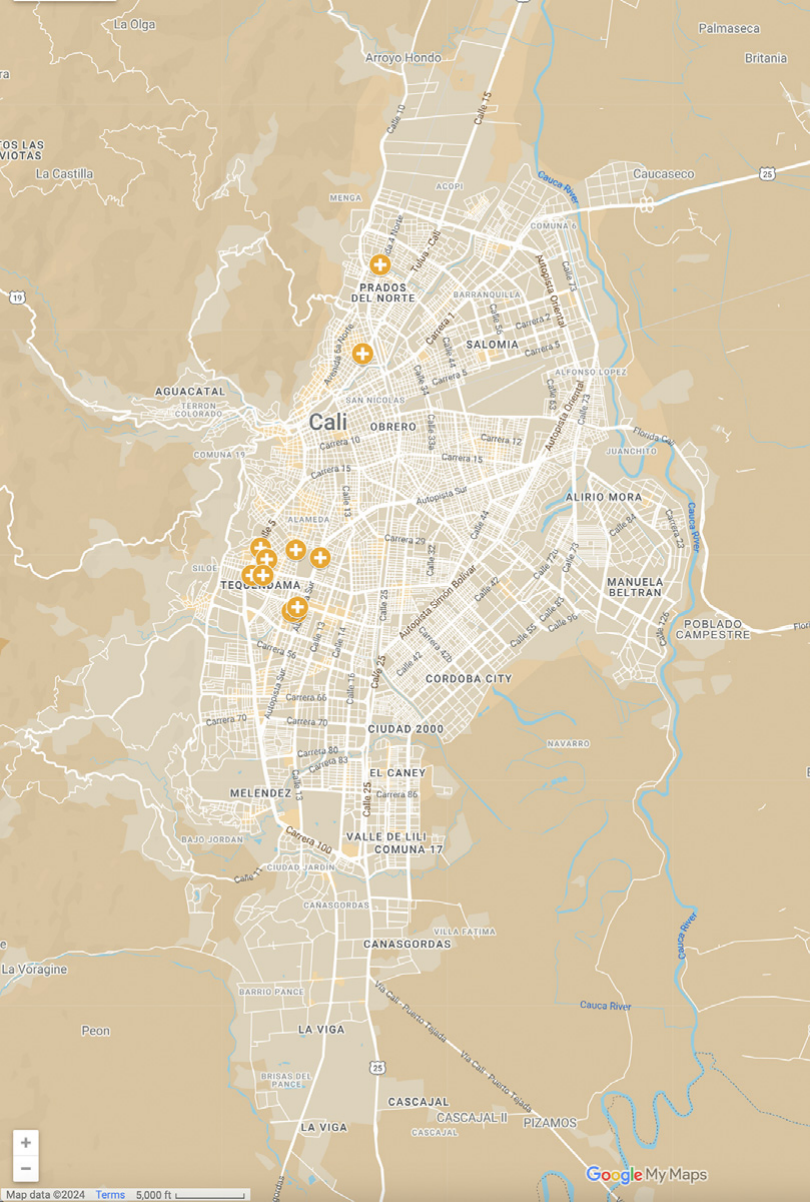
Location of haemodialysis units in Cali, 2020.^39^

The rationale and methods for this study follow previously published studies from our group.^2^^,^^3^^,^^37^ This study assesses accessibility by car to the service with the shortest travel time among the 11 haemodialysis services, which together had 370 haemodialysis chairs in 2020 (Fig. 1). Cali is the third-largest city in Colombia by population and the largest in the Colombian Pacific region.

According to an international traffic scorecard, Cali was the fifth-most congested city in South America and 76th globally.^40^ It has been a magnet for migration and an epicentre for social unrest in 2021 during the COVID-19 pandemic.^41^, ^42^, ^43^ Traffic congestion fluctuated widely in 2020 due to COVID-19 pandemic-related quarantines, stay-at-home orders, and traffic restrictions, such as those implemented in November.^37^^,^^44^, ^45^, ^46^

ESRD has increased in Cali recently, affecting 128 per 100,000 people in 2020. Rates increase with age, especially after age of 30. In 2020, 1545 people underwent haemodialysis in Cali, with the vast majority being adults.^47^^,^^48^ Patients typically travel to haemodialysis appointments by car (private or for hire), with a family member. In Colombia, insurers have contracts with health-providing institutions (IPS in Spanish), and these contracts define which health services patients can access, leading to fragmented health services provision. We did not match patients with insurance and provider contracts.

About 75% of Cali households rely on the informal economy, mainly the most vulnerable. Unemployment rates in 2020 were 17.3% for men, 24% for women, 24.2% for young men (15–28 years), and 34.3% for young women. About 11.7% of the population lives in multidimensional poverty, and 82.2% of urban Cali residents have some health insurance.^49^^,^^50^

To fulfil its constitutional and legal mandates, Cali's Territorial Health Plan has among its strategic objectives to manage and supervise the provision of health services for all its population.^51^

### Targeted sites/participants

We identified all 11 haemodialysis services in Cali using the Ministry of Health Special Registry of Health Services Providers (REPS in Spanish), which remained unchanged between July 2020 and January 2021. These services are concentrated along a corridor in the north–central part of Cali, following the major street, Calle Quinta, as indicated in Fig. 1.^39^ Typically, haemodialysis patients in Cali need to visit haemodialysis centres as home-based haemodialysis options are limited.

### Study design

We conducted two cross-sectional analyses by integrating public data sources in the web-based AMORE Platform, which displays DSTAM for each Traffic Analysis Zone (TAZ) to the TAZ hosting the haemodialysis services with the shortest travel time. TAZs are geographic units commonly used in transportation planning and are usually built on census blocks.^52^ Travel times are typically short within a TAZ, but populations are not evenly distributed in TAZs. Therefore, travel times from each of 507 TAZs in Cali were measured from the population-adjusted geometric centroids.^53^ The 11 haemodialysis services in Cali are concentrated in six TAZs.

**Fig. 2:**
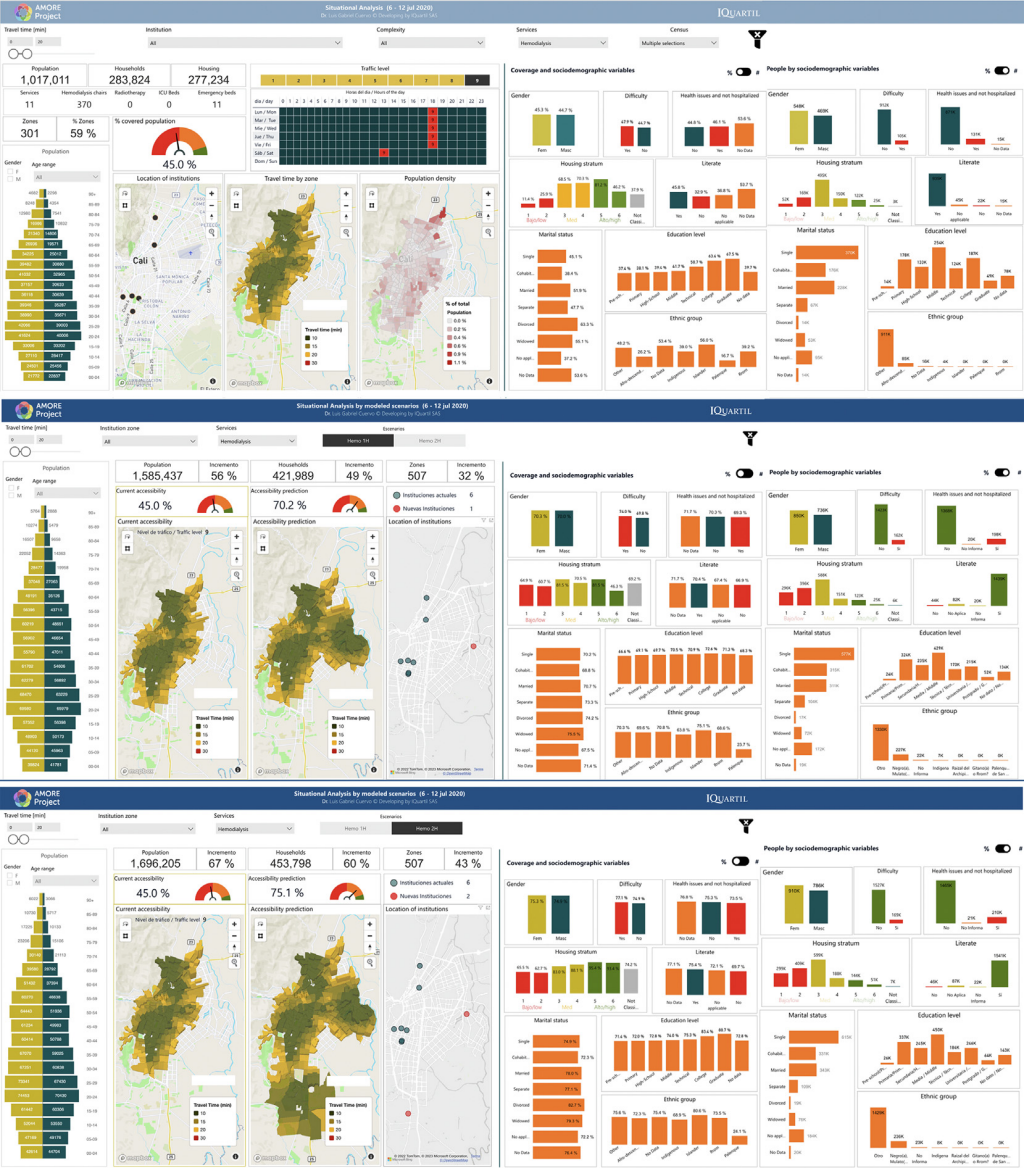
AMORE Platform interfaces with situational and predictive analyses, 6–12 July 2020.^39^^,^^51^

**Fig. 3:**
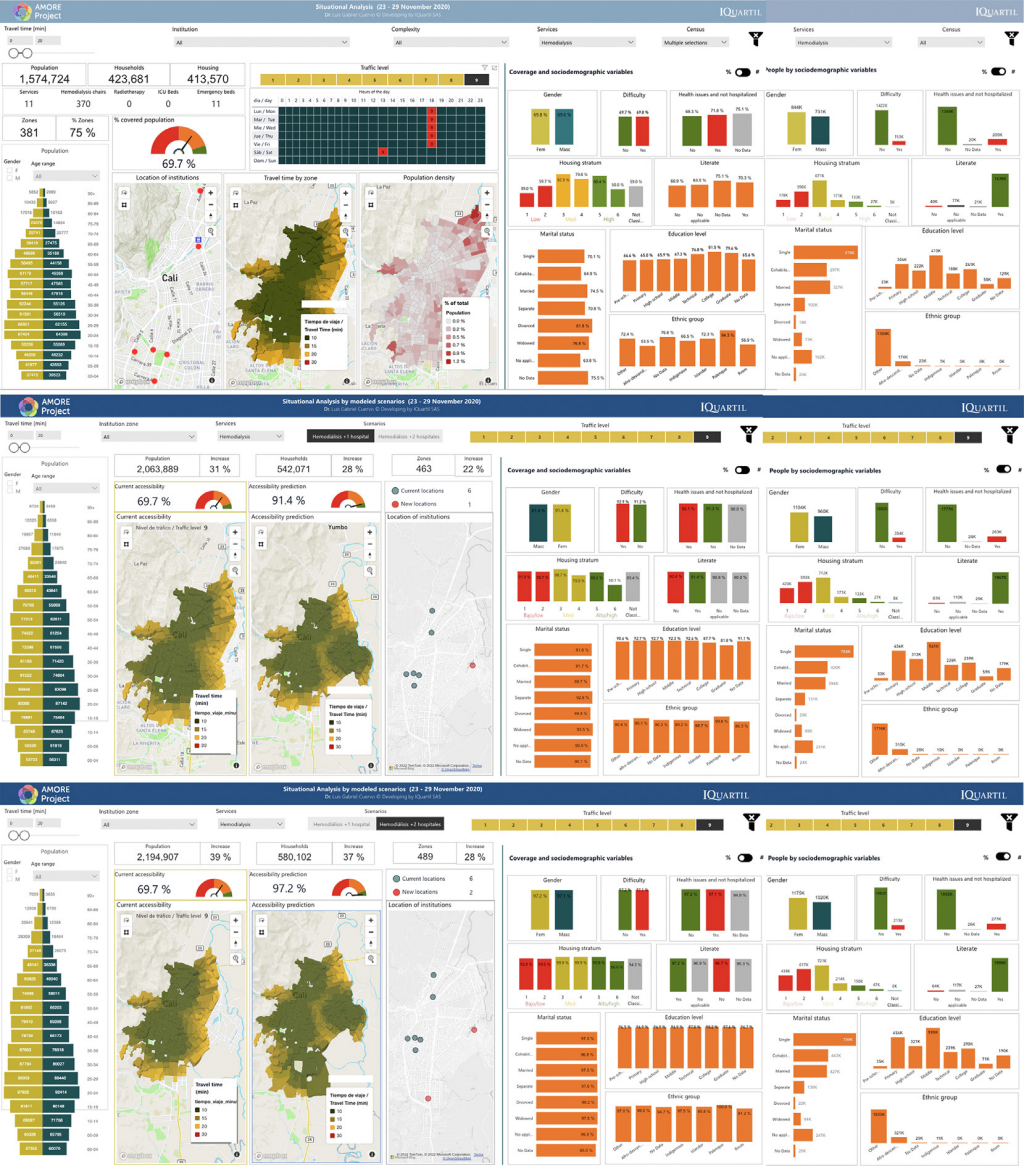
AMORE Platform interfaces with situational and predictive analyses, 23–29 Nov 2020.^39^^,^^51^

Data for the cross-sectional analyses was obtained from the web-based AMORE Platform (Figs. 2 and 3), hosted by iQuartil SAS and developed under the leadership of the principal investigator (LGC) and the lead data scientist (DC)^q^.^54^^,^^55^

The AMORE Platform is constructed by integrating the following data sources:

• 2018 National Census Data for Cali, obtained from the official public databases of the Colombian National Department of Statistics (DANE in Spanish), and adjusted to 2020.^3^^,^^56^^,^^57^
• The administrative divisions of Cali were obtained from government sources: the IDESC Geoportal, Traffic Analysis Zones (TAZs), and census block sectors.^58^^,^^59^
• Google's Distance Matrix API. For this test case assessment of urban Cali, data were downloaded on July 3, for the week of 6–12 July 2021. Predicted times were downloaded on 27 October for the week of 23–29 November 2020.^60^
• The 11 haemodialysis services in Cali were identified using the REPS registry.^39^ These services were in six TAZs, with one TAZ hosting four services and another TAZ hosting three.

Data for this case study were a subset of the data used by the AMORE Project to measure accessibility to 30 health services: 14 tertiary care emergency departments, 11 haemodialysis services, and five radiotherapy services. These 30 services were in 15 TAZs, with one TAZ hosting seven services, another TAZ four, six TAZ hosting two, and seven hosting a single service. Had the project focused on haemodialysis only, the eleven services would have been studied by assessing travel times to six TAZs.^3^

Measuring DSTAM for the three types of services (tertiary care emergencies, haemodialysis, and radiotherapy), using traffic congestion clusters and TAZs brought efficiencies and reduced travel time data download cost and complexity. Measurements were reduced from 43.1 million data points (507 origins to 506 destinations for 168 h weekly) to a sample of 68,445 (507 origins to 15 destinations for 9 traffic congestion clusters) for each week analysed.^3^^,^^37^ Using predictive analytics based on the sample, an estimated 2.3 million travel times (507 origins to 506 destinations in 9 traffic congestion clusters) was assessed for each week analysed. The efficiencies introduced by clustering travel times and using TAZ and predictive modelling reduced data downloads an estimated 630-fold. Measurement weeks were chosen out of interest in exploring accessibility behaviour during the COVID-19 pandemic and opportunity; we had everything in place to complete the data downloads, update the platform, and validate the data. Both assessments were completed in weeks with no holidays.

### Variables for study outcomes

The main study outcome was the proportion of urban dwellers likely to reach a haemodialysis service within 20 min by car. We set a 20-min travel-time threshold to predict the potential impact of adding new services in one or two strategic locations to optimise accessibility.^3^ Data were disaggregated by age, self-reported sex, education level attained, literacy, marital status, and household economic stratum based on electricity bill stratification. Other study outcomes included accessibility measured at 10-min intervals, by household economic stratification. Additional information can be obtained through the web-based AMORE Platform, where readers can explore scenarios by adjusting parameters.^30^^,^^55^^,^^61^

Travel-time thresholds were based on expert advice from AMORE Project collaborative group contributors, including patient advocates. We found no international standards and only assessed travel time by car (private and for hire), which will likely be unevenly distributed across socioeconomic groups.

**Fig. 4:**
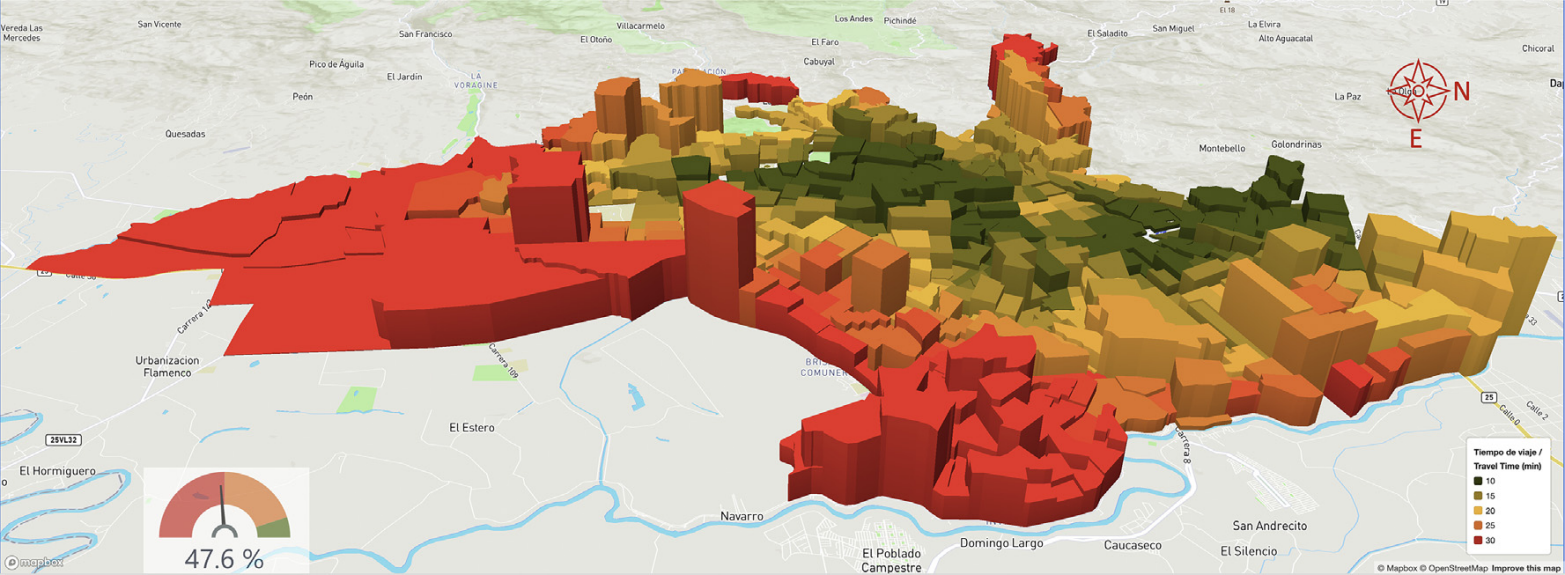
Characteristic accessibility during day hours, traffic congestion level.

**Fig. 5:**
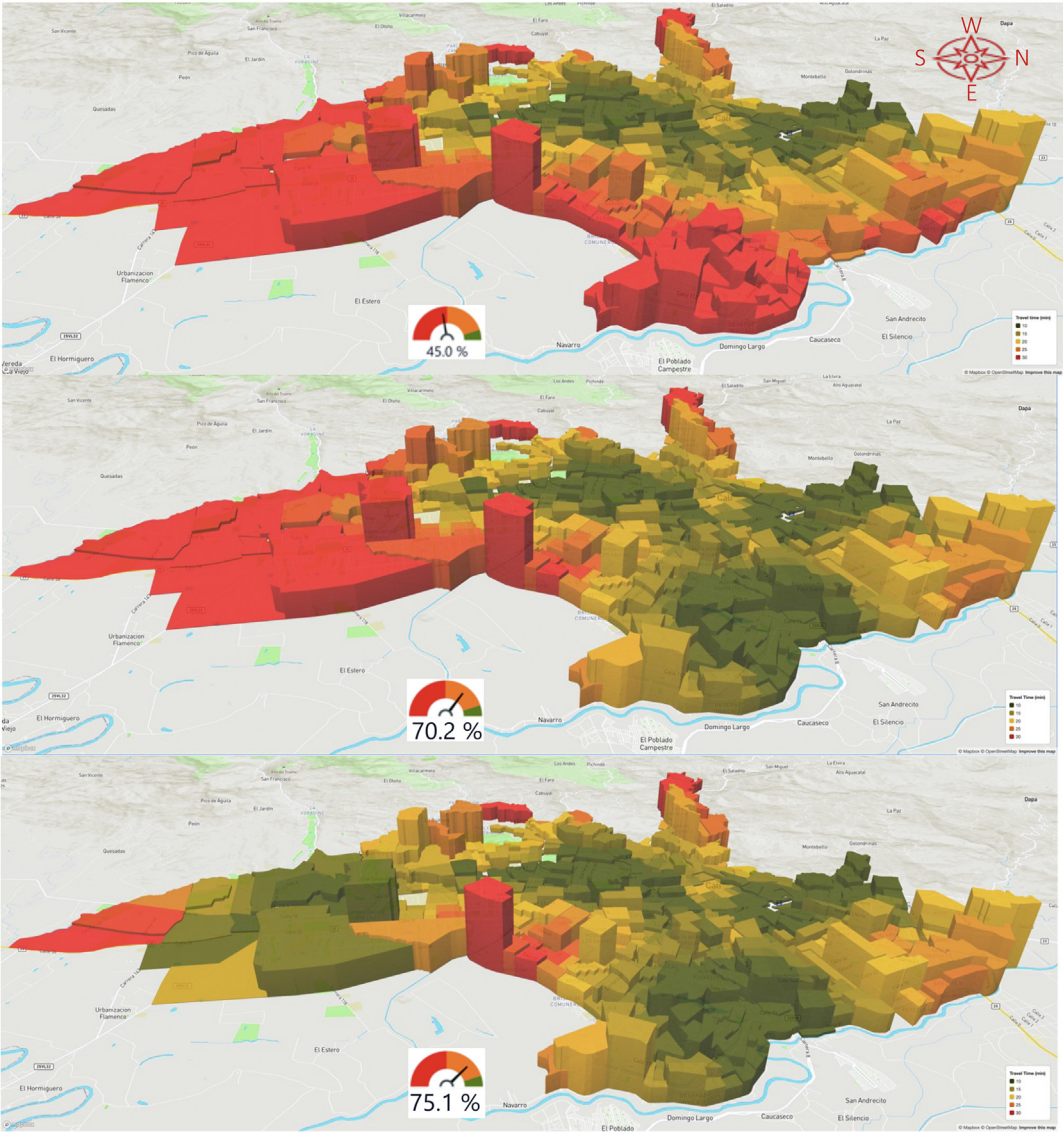
Predicted accessibility with new services in selected TAZs, 6–12 July 2020.

**Fig. 6:**
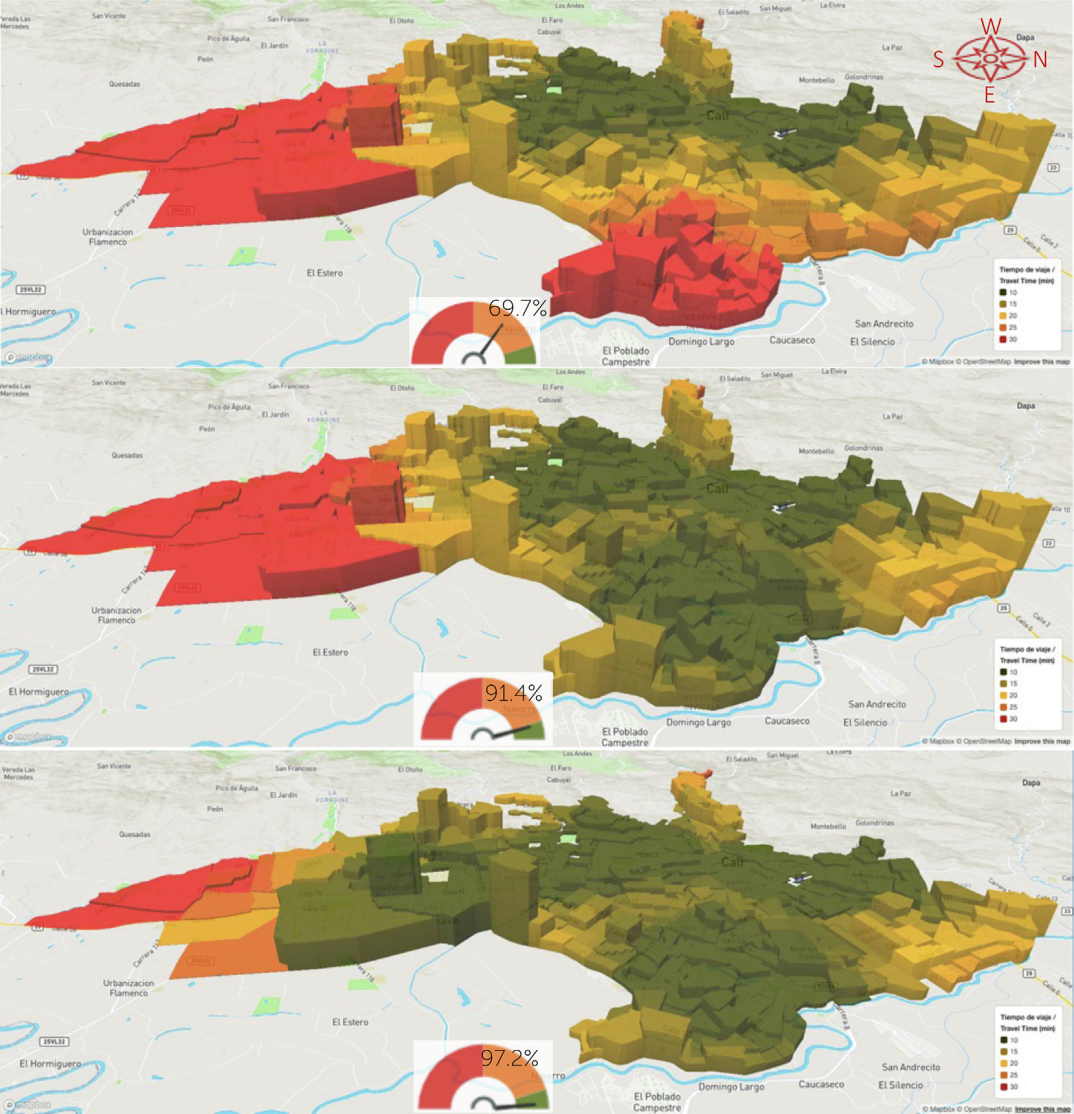
Predicted accessibility with new services in selected TAZs, 23–29 November 2020.

Data representing primary outcomes are displayed using graphs, tables, and colour-coded choropleth maps with the isochrones of the cumulative opportunities for accessibility, presenting travel times and population density for each TAZ.^35^^,^^36^ For the predictive and prescriptive analyses, the one or two TAZs where new services would optimise accessibility were identified, and results were disaggregated by sociodemographic characteristics. The web-based platform also displays this data as colour-coded choropleth maps that can be rotated to provide a 3D perspective (Fig. 4, Fig. 5, Fig. 6), with each choropleth representing a TAZ and their height representing its population density. These maps reveal the relationship between population density and geographic location with travel times. The colour code should help stakeholders and sector leaders agree on a common goal, such as “painting the city green” by putting all people within a 20-min travel-time threshold from a service.^62^

Traffic clusters range from free-flow (level 1) to peak traffic (level 9). Household socioeconomic stratification in Colombia has an incremental range from 1 to 6, with six representing the highest-income household group. Household socioeconomic stratum was categorised into Low (levels 1 and 2), middle (3 and 4), and high (5 and 6). Earlier publications have described the definition of traffic levels and subgroup variation measurement.^3^^,^^37^^,^^53^ The platform disaggregates data by education, ethnicity, self-reported sex, age, and other sociodemographic variables. Sociodemographic characteristics, TAZs, travel times, traffic levels, and institutions can be filtered and combined for analysis.

### Role of the funding source

The authors have not declared a specific grant for this research from any funding agency in public, commercial or not-for-profit sectors.

## Results

Cali has substantial traffic congestion and remains congested through most of the day and evening, even on weekends. Traffic level 8, the second highest, accounts for 40/168 (24%) hours of the week (Fig. 4). Traffic clusters are skewed, with most activity hours having high-traffic congestion levels.^37^

Figs. 2 and 3 are screenshots from AMORE Platform interfaces that display the variables and disaggregated data for the situational and predictive analyses for the July and November assessments. These analyses include baseline data and estimate accessibility after adding services in one or two additional TAZs.

Figs. 5 and 6, illustrate the impact of traffic congestion on accessibility under assessed and predicted scenarios. These results point to the locations where new services would maximise urban accessibility and provide overall and disaggregated estimates and variations for the July and November assessments.

**Fig. 7:**
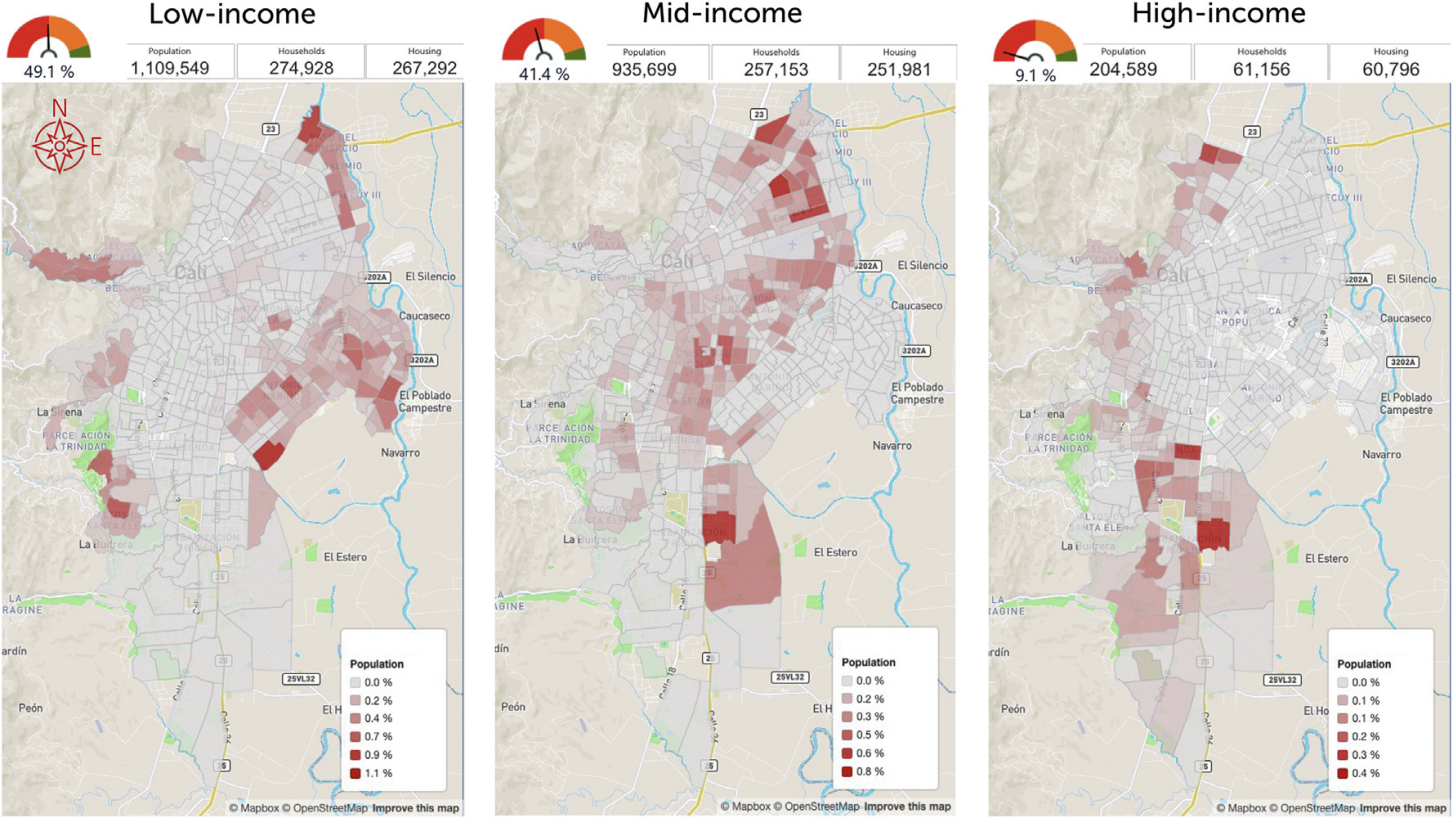
Housing distribution by socioeconomic stratification, Cali.

Population characteristics are presented in Table 1. About half of Cali residents live in low-income housing, 40% live in middle-income households, and about 9% live in high-income housing, including live-in domestic workers. The southern end of Cali is inhabited mainly by high-income households living in villas. The choropleth map shows that most of the population and the most densely populated areas are towards the periphery of Cali and far from haemodialysis services (Fig. 7). Travel-time comparisons between 6 and 12 July and 23–29 November 2020 show that traffic was notably lighter in November, when COVID-19 traffic restrictions were in place. The effect of these measures varied throughout city sectors (Figs. 5 and 6).

**Table 1:** 20-minute accessibility to haemodialysis in Cali by socio-demographic group.

Situational analysis 20 min accessibility to the nearest hemodialysis service (Population)	July 2020	November 2020	Variation	Total population	%	Accessibility july	Accessibility november	Subgroup variation
	1,017,011	1,574,724	*557,713*	2,258,823		45.0%	69.7%	
**Socio-economic stratum**								
Low	221,296	568,115	*346,819*	1,109,549	49.1%	19.9%	51.2%	*31.3%*
Middle	645,076	841,224	*196,148*	935,699	41.4%	68.9%	89.9%	*21.0%*
High	147,229	160,082	*12,853*	204,589	9.1%	72.0%	78.2%	*6.3%*
N.D.	3410	5303	*1893*	8986	0.4%	37.9%	59.0%	*21.1%*
**Ethnicity**								
Afrodescendent	85,481	174,224	*88,743*	325,865	14.4%	26.2%	53.5%	*27.2%*
Rrom (nomadic)	40	58	*18*	102	0.0%	39.2%	56.9%	*17.6%*
Indigenous	4339	7394	*3055*	11,112	0.5%	39.0%	66.5%	*27.5%*
Islander/Raizal	214	276	*62*	382	0.0%	56.0%	72.3%	*16.2%*
Other (Caucasian, Mestizo)	910,535	1,369,069	*458,534*	1,890,491	83.7%	48.2%	72.4%	*24.3%*
Palenque	41	231	*190*	245	0.0%	16.7%	94.3%	*77.6%*
N.D.	16,361	23,472	*7111*	30,626	1.4%	53.4%	76.6%	*23.2%*
**Educational level**								
Graduate degree	48,919	57,641	*8722*	72,441	3.2%	67.5%	79.6%	*12.0%*
Bachelor's Degree	187,250	240,652	*53,402*	295,319	13.1%	63.4%	81.5%	*18.1%*
Technical	123,846	187,591	*63,745*	244,160	10.8%	50.7%	76.8%	*26.1%*
Middle	254,015	409,594	*155,579*	608,429	26.9%	41.7%	67.3%	*25.6%*
High School	132,875	222,293	*89,418*	337,065	14.9%	39.4%	65.9%	*26.5%*
Primary	178,303	304,450	*126,147*	468,206	20.7%	38.1%	65.0%	*26.9%*
Pre-school	13,559	23,360	*9801*	36,294	1.6%	37.4%	64.4%	*27.0%*
No data	78,244	129,143	*50,899*	196,909	8.7%	39.7%	65.6%	*25.8%*
**Literacy**								
Literate	935,369	1,436,134	*500,765*	2,043,041	90.4%	45.8%	70.3%	*24.5%*
No literacy	21,864	40,417	*18,553*	66,383	2.9%	32.9%	60.9%	*27.9%*
N.A.	44,609	76,938	*32,329*	121,140	5.4%	36.8%	63.5%	*26.7%*
N.D.	15,169	21,235	*6066*	28,259	1.3%	53.7%	75.1%	*21.5%*
**Gender/Sex**								
Fem	547,601	844,077	*296,476*	1,208,617	53.5%	45.3%	69.8%	*24.5%*
Masc	469,410	730,647	*261,237*	1,050,206	46.5%	44.7%	69.6%	*24.9%*
**Civil status**								
Single	370,444	575,999	*205,555*	821,536	36.4%	45.1%	70.1%	*25.0%*
Married or cohabitation	403,517	624,355	*220,838*	896,958	39.7%	45.0%	69.6%	*24.6%*
Divorced or separated	81,711	118,628	*36,917*	163,980	7.3%	49.8%	72.3%	*22.5%*
Widow	52,705	73,457	*20,752*	95,611	4.2%	55.1%	76.8%	*21.7%*
N.A.	94,566	162,468	*67,902*	254,492	11.3%	37.2%	63.8%	*26.7%*
N.D.	14,068	19,817	*5749*	26,246	1.2%	53.6%	75.5%	*21.9%*
**Age**								
0–4	44,609	76,938	*32,329*	121,140	5.4%	36.8%	63.5%	*26.7%*
0–14	150,093	256,909	*106,816*	400,527	17.7%	37.5%	64.1%	*26.7%*
5–14	105,484	179,971	*74,487*	279,387	12.4%	37.8%	64.4%	*26.7%*
15–24	147,838	242,147	*94,309*	363,311	16.1%	40.7%	66.7%	*26.0%*
15–59	657,107	1,027,559	*370,452*	1,482,069	65.6%	44.3%	69.3%	*25.0%*
15–64	716,344	1,111,425	*395,081*	1,595,016	70.6%	44.9%	69.7%	*24.8%*
60+	209,811	290,256	*80,445*	376,227	16.7%	55.8%	77.1%	*21.4%*
65+	150,574	206,390	*55,816*	263,280	11.7%	57.2%	78.4%	*21.2%*
80+	40,103	52,080	*11,977*	64,100	2.8%	62.6%	81.2%	*18.7%*

Italics are used to show the difference/variation between figures on columns compared (located to the left).

The images and data revealed that services are concentrated, and most of the population incurs long journeys, particularly the poorest and those from minority ethnic groups (Supplemental File 1, Table 1).

### Placement of new services to optimise equitable accessibility

**Fig. 8:**
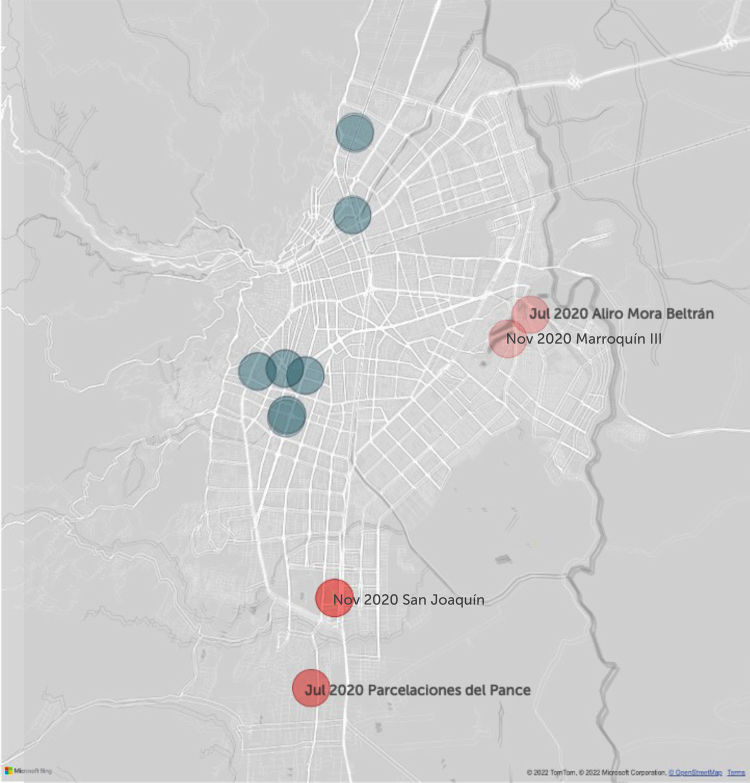
The predicted 1–2 TAZs where new services optimise accessibility.

The optimal location for maximising accessibility changed between neighbouring TAZs between July and November but remained close and within minutes. For July, the best TAZ was in the Alirio Mora Beltrán neighbourhood; for November, it was in the neighbouring Marroquin III TAZ. Both TAZs are in the eastern Agua Blanca district (Fig. 8). If services were added in two TAZs, the TAZ initially identified would remain the same in the independent predictions. The second recommended TAZ was for Parcelaciones del Pance in July and San Joaquín in November; these southern neighbourhoods are adjacent (Fig. 8).

**Fig. 9:**
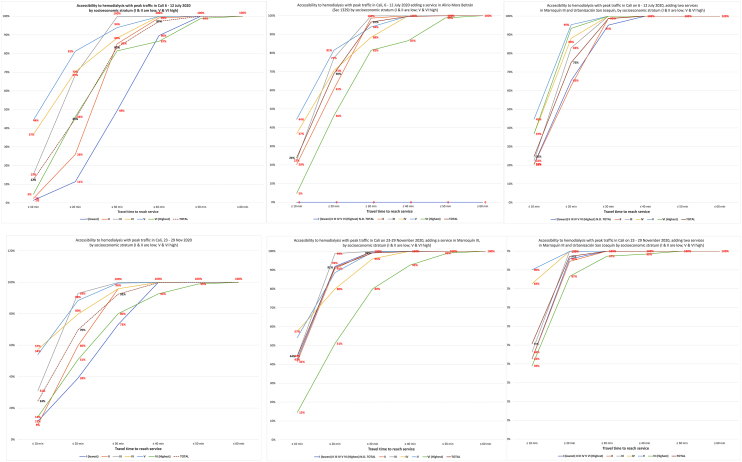
Traffic variations and their effect (July vs November 2020) 6–12 July vs 23–29 November 2020.

Adding new haemodialysis service(s) to the recommended locations — in Alirio Mora Beltran or Marroquin III TAZ in the eastern district of Agua Blanca, with access to all patients — would greatly improve accessibility for the entire city (Tables 2 and 3; Figs. 5 and 6). In this scenario, we estimated that accessibility rose from 1,017,011 (45.0%) to 1,585,437 (70.2%) for July and from 1,574,724 (69.7%) to 2,063,889 (91.4%) for November 2020. These results levelled accessibility specifically by benefiting the lowest-income residents of Cali. For example, in the July estimates, accessibility for the lowest-income group (i.e., stratum 1) rose from 51,866 (11.4%) to 296,298 (64.9%) (Fig. 9). Populations living in the southernmost high-income suburban areas benefited the least from the new service.

**Table 2:** Predicted accessibility to the haemodialysis service with the shortest travel times (absolute figures).

Predicted 20 min accessibility to the nearest hemodialysis service, by subgroup	Population July 2020	Add 1 service	Variation	Add 2 services	Variation	Benefit of the second service	Population November 2020	Add 1 service	Variation +1 service November	Add 2 services	Variation +2 serv. november vs baseline	Benefit of the second service
	1,017,011	1,585,437	*568,426*	1,696,205	*679,194*	*110,768*	1,574,724	2,063,889	*489,165*	2,194,907	*620,183*	*131,018*
**Socio-economic stratum**												
Low	221,296	692,535	*471,239*	708,285	*486,989*	*15,750*	568,115	1,012,179	*444,064*	1,054,841	*486,726*	*42,662*
Middle	645,076	738,967	*93,891*	787,189	*142,113*	*48,222*	841,224	883,232	*42,008*	934,581	*93,357*	*51,349*
High	147,229	147,714	*485*	194,064	*46,835*	*46,350*	160,082	160,448	*366*	197,011	*36,929*	*36,563*
N.D.	3410	6221	*2811*	6667	*3257*	*446*	5303	8030	*2727*	8474	*3171*	*444*
**Ethnicity**												
Afrodescendent	85,481	226,665	*141,184*	235,681	*150,200*	*9016*	174,224	309,773	*135,549*	320,945	*146,721*	*11,172*
Rrom (nomadic)	40	70	*30*	75	*35*	*5*	58	88	*30*	93	*35*	*5*
Indigenous	4339	7093	*2754*	7661	*3322*	*568*	7394	10,023	*2629*	10,830	*3436*	*807*
Islander/Raizal	214	287	*73*	308	*94*	*21*	276	339	*63*	366	*90*	*27*
Other (Caucasian, Mestizo)	910,535	1,329,587	*419,052*	1,429,331	*518,796*	*99,744*	1,369,069	1,715,776	*346,707*	1,833,419	*464,350*	*117,643*
Palenque	41	58	*17*	59	*18*	*1*	231	244	*13*	245	*14*	*1*
N.D.	16,361	21,677	*5316*	23,090	*6729*	*1413*	23,472	27,646	*4174*	29,009	*5537*	*1363*
**Educational level**												
Graduate degree	48,919	51,673	*2754*	64,274	*15,355*	*12,601*	57,641	59,250	*1609*	70,683	*13,042*	*11,433*
Bachelor's degree	187,250	214,500	*27,250*	246,213	*58,963*	*31,713*	240,652	259,026	*18,374*	290,113	*49,461*	*31,087*
Technical	123,846	173,029	*49,183*	183,949	*60,103*	*10,920*	187,591	225,703	*38,112*	238,756	*51,165*	*13,053*
Middle	254,015	429,219	*175,204*	449,999	*195,984*	*20,780*	409,594	561,317	*151,723*	589,778	*180,184*	*28,461*
High School	132,875	234,812	*101,937*	245,256	*112,381*	*10,444*	222,293	312,505	*90,212*	326,680	*104,387*	*14,175*
Primary	178,303	323,554	*145,251*	337,279	*158,976*	*13,725*	304,450	433,804	*129,354*	453,532	*149,082*	*19,728*
Pre-school	13,559	24,185	*10,626*	25,912	*12,353*	*1727*	23,360	32,893	*9533*	35,018	*11,658*	*2125*
No data	78,244	134,465	*56,221*	143,323	*65,079*	*8858*	129,143	179,391	*50,248*	190,347	*61,204*	*10,956*
**Literacy**												
Literate	935,369	1,439,178	*503,809*	1,540,840	*605,471*	*101,662*	1,436,134	1,867,076	*430,942*	1,986,355	*550,221*	*119,279*
No literacy	21,864	44,390	*22,526*	46,272	*24,408*	*1882*	40,417	61,347	*20,930*	64,213	*23,796*	*2866*
N.A.	44,609	81,605	*36,996*	87,318	*42,709*	*5713*	76,938	110,033	*33,095*	117,422	*40,484*	*7389*
N.D.	15,169	20,264	*5095*	21,775	*6606*	*1511*	21,235	25,433	*4198*	26,917	*5682*	*1484*
**Gender/Sex**												
Fem	547,601	849,850	*302,249*	910,080	*362,479*	*60,230*	844,077	1,104,162	*260,085*	1,175,110	*331,033*	*70,948*
Masc	469,410	735,587	*266,177*	786,125	*316,715*	*50,538*	730,647	959,727	*229,080*	1,019,797	*289,150*	*60,070*
**Civil status**						*–*					*–*	*–*
Single	370,444	576,988	*206,544*	615,450	*245,006*	*38,462*	575,999	754,020	*178,021*	799,355	*223,356*	*45,335*
Married or cohabitation	403,517	625,534	*222,017*	673,554	*270,037*	*48,020*	624,355	813,993	*189,638*	870,343	*245,988*	*56,350*
Divorced or separated	81,711	120,329	*38,618*	127,626	*45,915*	*7297*	118,628	151,450	*32,822*	160,193	*41,565*	*8743*
Widow	52,705	2171	*−50,534*	75,848	*23,143*	*73,677*	73,457	89,402	*15,945*	93,550	*20,093*	*4148*
N.A.	94,566	171,688	*77,122*	183,663	*89,097*	*11,975*	162,468	231,388	*68,920*	246,543	*84,075*	*15,155*
N.D.	14,068	18,727	*4659*	20,064	*5996*	*1337*	19,817	23,636	*3819*	24,923	*5106*	*1287*
**Age**			*–*									*–*
0–4	44,609	81,605	*36,996*	87,318	*42,709*	*5713*	76,938	110,033	*33,095*	117,422	*40,484*	*7389*
0–14	150,093	270,764	*120,671*	289,257	*139,164*	*18,493*	256,909	364,761	*107,852*	387,896	*130,987*	*23,135*
5–14	105,484	189,159	*83,675*	201,939	*96,455*	*12,780*	179,971	254,728	*74,757*	270,474	*90,503*	*15,746*
15–24	147,838	249,309	*101,471*	266,631	*118,793*	*17,322*	242,147	331,487	*89,340*	352,301	*110,154*	*20,814*
15–59	657,107	1,031,825	*374,718*	1,696,205	*1,039,098*	*664,380*	1,027,559	1,349,753	*322,194*	1,439,686	*412,127*	*89,933*
15–64	716,344	1,115,142	*398,798*	1,196,118	*479,774*	*80,976*	1,111,425	1,453,704	*342,279*	1,549,551	*438,126*	*95,847*
60+	209,811	282,848	*73,037*	299,656	*89,845*	*16,808*	290,256	349,375	*59,119*	367,325	*77,069*	*17,950*
65+	150,574	199,531	*48,957*	210,830	*60,256*	*11,299*	206,390	245,424	*39,034*	257,460	*51,070*	*12,036*
80+	40,103	50,570	*10,467*	52,893	*12,790*	*2323*	52,080	60,472	*8392*	62,987	*10,907*	*2515*

Italics are for the calculations made on differences when adding one or two services. Columns in non-italic represent the raw data from the platform.

**Table 3:** Predicted accessibility to the haemodialysis service with the shortest travel times (relative figures).

Predicted 20 min accessibility to the nearest hemodialysis service, by subgroup (%)	July 2020 (% subgroup)	Add 1 service	Variation	Add 2 services	Variation adding 2 services	Benefit of the second service	Nov 2020 (%)	Add 1 service	Variation +1 service november	Add 2 services	Variation +2 serv. november vs baseline	Benefit of the second service	Subgroup's population
**Socio-economic stratum**													
Low	19.9%	62.4%	*42.5%*	63.8%	*43.9%*	1.4%	51.2%	91.2%	*40.0%*	95.1%	*43.9%*	3.8%	1,109,549
Middle	68.9%	79.0%	*10.0%*	84.1%	*15.2%*	5.2%	89.9%	94.4%	*4.5%*	99.9%	*10.0%*	5.5%	935,699
High	72.0%	72.2%	*0.2%*	94.9%	*22.9%*	22.7%	78.2%	78.4%	*0.2%*	96.3%	*18.1%*	17.9%	204,589
N.D.	37.9%	69.2%	*31.3%*	74.2%	*36.2%*	5.0%	59.0%	89.4%	*30.3%*	94.3%	*35.3%*	4.9%	8986
**Ethnicity**													
Afrodescendent	26.2%	69.6%	*43.3%*	72.3%	*46.1%*	2.8%	53.5%	95.1%	*41.6%*	98.5%	*45.0%*	3.4%	325,865
Rrom (nomadic)	39.2%	68.6%	*29.4%*	73.5%	*34.3%*	4.9%	56.9%	86.3%	*29.4%*	91.2%	*34.3%*	4.9%	102
Indigenous	39.0%	63.8%	*24.8%*	68.9%	*29.9%*	5.1%	66.5%	90.2%	*23.7%*	97.5%	*30.9%*	7.3%	11,112
Islander/Raizal	56.0%	75.1%	*19.1%*	80.6%	*24.6%*	5.5%	72.3%	88.7%	*16.5%*	95.8%	*23.6%*	7.1%	382
Other (Caucasian, Mestizo)	48.2%	70.3%	*22.2%*	75.6%	*27.4%*	5.3%	72.4%	90.8%	*18.3%*	97.0%	*24.6%*	6.2%	1,890,491
Palenque	16.7%	23.7%	*6.9%*	24.1%	*7.3%*	0.4%	94.3%	99.6%	*5.3%*	100.0%	*5.7%*	0.4%	245
N.D.	53.4%	70.8%	*17.4%*	75.4%	*22.0%*	4.6%	76.6%	90.3%	*13.6%*	94.7%	*18.1%*	4.5%	30,626
**Educational level**						0.0%							
Graduate degree	67.5%	71.3%	*3.8%*	88.7%	*21.2%*	17.4%	79.6%	81.8%	*2.2%*	97.6%	*18.0%*	15.8%	72,441
Bachelor Degree	63.4%	72.6%	*9.2%*	83.4%	*20.0%*	10.7%	81.5%	87.7%	*6.2%*	98.2%	*16.7%*	10.5%	295,319
Technical	50.7%	70.9%	*20.1%*	75.3%	*24.6%*	4.5%	76.8%	92.4%	*15.6%*	97.8%	*21.0%*	5.3%	244,160
Middle	41.7%	70.5%	*28.8%*	74.0%	*32.2%*	3.4%	67.3%	92.3%	*24.9%*	96.9%	*29.6%*	4.7%	608,429
High School	39.4%	69.7%	*30.2%*	72.8%	*33.3%*	3.1%	65.9%	92.7%	*26.8%*	96.9%	*31.0%*	4.2%	337,065
Primary	38.1%	69.1%	*31.0%*	72.0%	*34.0%*	2.9%	65.0%	92.7%	*27.6%*	96.9%	*31.8%*	4.2%	468,206
Pre-school	37.4%	66.6%	*29.3%*	71.4%	*34.0%*	4.8%	64.4%	90.6%	*26.3%*	96.5%	*32.1%*	5.9%	36,294
No data	39.7%	68.3%	*28.6%*	72.8%	*33.1%*	4.5%	65.6%	91.1%	*25.5%*	96.7%	*31.1%*	5.6%	196,909
**Literacy**													
Literate	45.8%	70.4%	*24.7%*	75.4%	*29.6%*	5.0%	70.3%	91.4%	*21.1%*	97.2%	*26.9%*	5.8%	2,043,041
No literacy	32.9%	66.9%	*33.9%*	69.7%	*36.8%*	2.8%	60.9%	92.4%	*31.5%*	96.7%	*35.8%*	4.3%	66,383
N.A.	36.8%	67.4%	*30.5%*	72.1%	*35.3%*	4.7%	63.5%	90.8%	*27.3%*	96.9%	*33.4%*	6.1%	121,140
N.D.	53.7%	71.7%	*18.0%*	77.1%	*23.4%*	5.3%	75.1%	90.0%	*14.9%*	95.3%	*20.1%*	5.3%	28,259
**Gender/Sex**													
Fem	45.3%	70.3%	*25.0%*	75.3%	*30.0%*	5.0%	69.8%	91.4%	*21.5%*	97.2%	*27.4%*	5.9%	1,208,617
Masc	44.7%	70.0%	*25.3%*	74.9%	*30.2%*	4.8%	69.6%	91.4%	*21.8%*	97.1%	*27.5%*	5.7%	1,050,206
**Civil status**						0.0%							
Single	45.1%	70.2%	*25.1%*	74.9%	*29.8%*	4.7%	70.1%	91.8%	*21.7%*	97.3%	*27.2%*	5.5%	821,536
Married or cohabitation	45.0%	69.7%	*24.8%*	75.1%	*30.1%*	5.4%	69.6%	90.8%	*21.1%*	97.0%	*27.4%*	6.3%	896,958
Divorced or separated	49.8%	73.4%	*23.6%*	77.8%	*28.0%*	4.4%	72.3%	92.4%	*20.0%*	97.7%	*25.3%*	5.3%	163,980
Widow	55.1%	2.3%	*−52.9%*	79.3%	*24.2%*	77.1%	76.8%	93.5%	*16.7%*	97.8%	*21.0%*	4.3%	95,611
N.A.	37.2%	67.5%	*30.3%*	72.2%	*35.0%*	4.7%	63.8%	90.9%	*27.1%*	96.9%	*33.0%*	6.0%	254,492
N.D.	53.6%	71.4%	*17.8%*	76.4%	*22.8%*	5.1%	75.5%	90.1%	*14.6%*	95.0%	*19.5%*	4.9%	26,246
**Age**													
0–4	36.8%	67.4%	*30.5%*	72.1%	*35.3%*	4.7%	63.5%	90.8%	*27.3%*	96.9%	*33.4%*	6.1%	121,140
0–14	37.5%	67.6%	*30.1%*	72.2%	*34.7%*	4.6%	64.1%	91.1%	*26.9%*	96.8%	*32.7%*	5.8%	400,527
5–14	37.8%	67.7%	*29.9%*	72.3%	*34.5%*	4.6%	64.4%	91.2%	*26.8%*	96.8%	*32.4%*	5.6%	279,387
15–24	40.7%	68.6%	*27.9%*	73.4%	*32.7%*	4.8%	66.7%	91.2%	*24.6%*	97.0%	*30.3%*	5.7%	363,311
15–59	44.3%	69.6%	*25.3%*	114.4%	*70.1%*	44.8%	69.3%	91.1%	*21.7%*	97.1%	*27.8%*	6.1%	1,482,069
15–64	44.9%	69.9%	*25.0%*	75.0%	*30.1%*	5.1%	69.7%	91.1%	*21.5%*	97.1%	*27.5%*	6.0%	1,595,016
60+	55.8%	75.2%	*19.4%*	79.6%	*23.9%*	4.5%	77.1%	92.9%	*15.7%*	97.6%	*20.5%*	4.8%	376,227
65+	57.2%	75.8%	*18.6%*	80.1%	*22.9%*	4.3%	78.4%	93.2%	*14.8%*	97.8%	*19.4%*	4.6%	263,280
80+	62.6%	78.9%	*16.3%*	82.5%	*20.0%*	3.6%	81.2%	94.3%	*13.1%*	98.3%	*17.0%*	3.9%	64,100

Italics are used to show the difference/variation between figures on columns compared (located to the left).

Additionally adding services in the southern Pance neighbourhood would reduce differences for most subgroups, raising urban accessibility to 1,696,205 (75.1%) in July and 2,194,907 (97.2%) in November. Both results represent dramatic improvements over baseline assessments (Tables 2 and 3; Figs. 5 and 6).

Accessibility improvements seen between July and November added 557,713 people to the 20-min threshold in November, primarily benefitting low- and middle-income populations. Accessibility during peak traffic times rose 31.3% for low-income households (19.9%–51.2%) and 21.0% for middle-income households (68.9%–89.9%). Accessibility for high-income households rose just 6.2% (from 72.0% to 78.2%), perhaps because most families live close to services or in south-end villas that will still face long trips.

Subgroup improvements by other sociodemographic characteristics varied between 18% and 29%, with two exceptions. First, accessibility for people with graduate degrees rose 13.2%, reflecting November traffic restrictions and overlap in geographic distribution with high-income households. Second, accessibility rose 79.6% for the small Palenque people population concentrating around a cluster of TAZs.^37^

Choropleth maps show that offering haemodialysis in the eastern district of Agua Blanca would make haemodialysis accessible to densely populated areas of the city. These additions would nearly double overall urban accessibility and level accessibility rates in sociodemographic groups, reducing most inequities (Figs. 2 and 3).

The 10-min interval assessments by housing socioeconomic stratum also revealed links between income and accessibility, except for the highest-income households living in south-end villas. For these households, accessibility would only improve if services were added in the thinly populated south end of the city. Differences between income groups were noticeable throughout and worsened with traffic congestion. Adding services in the proposed TAZs reduced inequities even though these persisted for the shorter 10-min journeys (Fig. 9).

The analysis found that during peak traffic congestion and at most hours of the day (traffic levels 8 and 9), most people in the lowest-income stratum could not reach a haemodialysis service within 20 min. This was true in both the July and November assessments (11.4% and 39.6%, respectively) (Figs. 2, 3 and 9).

Data indicates that accessibility is an access barrier for low-income households, specifically in areas with high population density and outlying urban sectors (Supplemental File 1).

Table 2 (absolute figures) and Table 3 (relative subgroup proportions) provide data for the predicted accessibility to the haemodialysis service with the shortest travel times.

The 20-min threshold used in this study was deemed sound and appropriate for context by collaborative group members, including patient representatives. We found no benchmarks for travel times to haemodialysis. Time is a continuous variable; thus, an arbitrary threshold was chosen. An interactive interface enables stakeholders, including decision-makers, to test different assumptions and variables, assess the resulting data, draw conclusions and challenge current thinking.^30^

The haemodialysis sections of the AMORE Platform will be available at https://www.iquartil.net/proyectoAMORE/ for at least two years from the date of this publication.

## Discussion

The collaborative efforts within the AMORE Project are directed towards addressing the imperative for comprehensive methodologies that integrate health considerations into urban and territorial planning, particularly in urban settings characterised by heightened health inequities exacerbated by traffic congestion. The primary focus is on rectifying two notable deficiencies identified in existing studies: (1) the failure to systematically measure the impact of traffic congestion levels on both health equity and accessibility,^23^^,^^28^^,^^63^, ^64^, ^65^ and (2) the prevalent issue of insufficiently comprehending the intricate dynamics across interest groups that play a pivotal role in shaping health services planning and policies, resulting in a lack of a holistic approach.^2^^,^^4^^,^^5^^,^^30^^,^^31^^,^^66^ These identified limitations impede the practical application of research findings, hindering their utility in supporting effective governance, monitoring, and fostering public engagement and a shared narrative to achieve equitable accessibility to health services.^5^^,^^30^^,^^66^, ^67^, ^68^, ^69^, ^70^, ^71^

Our focus lies in urban and metropolitan areas facing challenges with traffic congestion. Our methodology introduces a practical framework for local advocates, governments, and service providers to enhance and monitor health equity and accessibility.^4^^,^^72^^,^^73^ Measurements are updatable for monitoring and can be extended to various health services, transportation modes, or locations. Furthermore, the approach is adaptable for refinement, development, and integration with other data. Notably, being straightforward and intuitive to residents of congested cities, travel times exhibit a stronger association with costs compared to distance measurements.^74^, ^75^, ^76^

In our preparatory phase, we surveyed studies that presented viewpoints from specific sectors such as mobility, health, or data sciences. However, we observed a gap in the literature where studies needed to establish connections between diverse stakeholders and relevant sectors, necessitating the role of evidence intermediaries or cultural brokers. While these studies often presented rich technical details and employed sophisticated methods, they generally needed the perspectives and contextual information essential for meaningful discussions with diverse stakeholders or for facilitating policy implementation.^5^^,^^30^^,^^66^^,^^73^^,^^77^, ^78^, ^79^ Dynamic geographical accessibility assessments are gaining traction^80^; however, our key informants expressed unfamiliarity with them, indicating that this remains a niche topic yet to be widely adopted in public policy and intersectoral actions.^4^^,^^30^^,^^81^^,^^82^

Key informants from the government, patient representatives, service providers, urban observatories, and civic leaders contributed to the design of the AMORE web-based platform. Prototypes were developed in the second semester of 2020, aligning with principles of knowledge translation, social appropriation, and participatory planning^5^^,^^30^^,^^82^, ^83^, ^84^, ^85^

DSTAM between the origin and destination TAZs allowed for accurate, granular and accessible assessments. Haemodialysis services are concentrated in areas distant from most of the population. Even during the early morning (5 AM) or late evening (11 PM) on weekdays, almost one-fifth of the population is beyond the 20-min travel threshold, including some of the poorest people and those living in outlying areas (Fig. 7). Our findings reveal haemodialysis accessibility inequalities, exposing vulnerable residents in outlying areas to longer journeys.^28^^,^^86^ Traffic congestion disproportionately affected people in the city's peripheries, particularly among low-income housing residents, certain ethnic groups like Afro-descendants, and individuals with lower education attainment.^87^, ^88^, ^89^ This study highlights an “inverse care law,” illustrating that vulnerable populations bear a disproportionate burden to access health services.^63^, ^64^, ^65^^,^^90^^,^^91^

The most vulnerable populations are likely to require haemodialysis and face higher costs, even if national insurance covers the treatment).^92^^,^^93^ Despite holistic approaches to address violence and social injustice in Cali, socioeconomic segregation has been pervasive, with land use and health services planning perpetuating the concentration of haemodialysis services in affluent areas of the city (Fig. 1).^51^^,^^94^, ^95^, ^96^, ^97^

Patient stories show that getting to services is challenging and physically demanding for patients living in outlying city areas. One haemodialysis patient explained, “*I spent 4 h in haemodialysis. Many other patients would spend hours commuting to and from haemodialysis, whether on their car or shared rides arranged by health providers. Long travel times added to treatment duration, taking most of the time patients and companions had for other activities.*” (Personal communication, Felipe Piquero, November 2022).^9^^,^^16^ The situation is even worse and riskier for those relying on public transportation. The scenarios studied here considered transportation by car, commonly used and deemed convenient by those seeking haemodialysis services.^9^^,^^14^^,^^87^^,^^90^^,^^98^ However, future studies could explore other means of transportation, link services with insurance, and integrate prevalence and incidence data or appointment availability to provide more refined information. They could also establish accessibility to services by being aware of appointments available or expanding to cover the entire metropolitan area and other communities accessing these services.

Applying the city's prevalence of haemodialysis (128 per 100,000 people) to July's estimations shows that adding services in Alirio Mora Beltrán would put 700,472 people within the 20-min threshold of those services, with an estimated 897 requiring haemodialysis; the figure could be higher considering that ESRD is more prevalent in low-income communities (Tables 2 and 3).

Adding services in Parcelaciones del Pance would put an additional 122,665 people within the 20-min threshold, which could result in an estimated 157 requiring haemodialysis. The two services would bring a population of 823,137 within the 20-min threshold, and about 1054 require haemodialysis (Tables 2 and 3).

While these estimations are simplistic and a proper one would have more nuances, they give an insight into the opportunities and possibilities of refining these test cases to guide decision-making.

The strategic location of new services will especially benefit populations in outlying areas and vulnerable groups such as low-income households, some ethnic groups, and people whose education attainment isn't high. It will also likely ease the demand, reduce the workload, and minimise the need for night schedules for existing services.

We found no DSTAM for haemodialysis services published before the commencement of our project in the first half of 2020 that considered traffic congestion levels.^29^ Despite calls for such studies, few assessments cover other treatments, and recently, these studies are emerging.^2^^,^^23^^,^^27^^,^^69^^,^^99^ None of the studies we identified provided a pathway to facilitate integration with urban and territorial planning or stakeholder engagement. A 2022 systematic review on geographic accessibility to radiotherapy found ten studies assessing travel time, and none considered traffic congestion gradients.^100^ In the field of obstetrics, a survey conducted in 2023 highlighted two studies/dashboards that assessed the geographical accessibility of services in low or middle-income countries, including our project.^101^ Recognizing the proposal as innovative and receiving positive feedback from key informants who deemed it worthwhile, we tested the approach. The intention is to explore its potential for replication in other cities experiencing traffic congestion in Colombia (using the same data sources), Latin America, and various regions globally.^3^^,^^53^^,^^80^

An immediate application of this data could be prioritising transportation or subsidising travel costs for people with longer travel times and vulnerable populations; data could be used to determine subsidy rates and to advance and monitor strategic objectives established in territorial health plans.^51^^,^^102^

Accessibility assessments revealed the problem and predictions point at solutions. The AMORE web-based platform used heuristic genetic algorithms to identify the 1 or 2 locations where new services would maximise urban accessibility. We compared peak traffic congestion accessibility for July and November. We predict that adding services to the proposed TAZ would greatly enhance urban accessibility and health equity to levels that resemble current low traffic congestion (Interactive with the AMORE web-based platform).^54^^,^^55^ This indicates that accessibility-related inequalities can be addressed through political and civic will and land use arrangements if new services, at or near the one or two TAZ where haemodialysis accessibility is maximised, can absorb the demand.

Despite accessibility figures varying substantially in our two measurements, the optimal locations for new haemodialysis services did not. Our assessments suggest adding haemodialysis services in the eastern Agua Blanca district that all patients can access. The location remained consistent whether the prediction was made for adding services in one or two TAZs at a time (Fig. 8).

Different stakeholders are likely to understand and peruse the reported data and graphs with the descriptive statistics of the populations able to reach the haemodialysis service with the shortest travel time within a time threshold (Table 1, Table 2, Table 3). Results were also presented in choropleth maps (heatmaps) to link the data to the territory, and in charts illustrating cumulative accessibility opportunities. These reports, multimedia, and the published web-based platform should be suitable for multistakeholder dialogues.^30^

Distinct elements of this innovation include an integral approach to health services planning providing: (1) an equity perspective using disaggregated sociodemographic information; (2) baseline and predicted assessments of traffic congestion's impact on geographical accessibility, established using DSTAM (3) simple charts, statistics, and cartography that integrate the territory, the population, haemodialysis services, and travel times that can be used to monitor or inform multistakeholder dialogues and participatory decision making. (4) a path to act on improving health equity and social justice.^2^^,^^3^^,^^28^^,^^30^^,^^103^^,^^104^ (5) options to test assumptions in the interactive platform, and^30^^,^^37^ (6) graphics that could unite stakeholders and sectors around common goals, such as “painting the city green”, placing services close to patients, and integrating health equity with urban and territorial planning.^2^^,^^5^^,^^28^^,^^105^

While all people requiring haemodialysis should have a service within a short travel time, this alone does not guarantee accessibility. Currently, coverage depends on agreements with service providers, and transportation is only sometimes provided. It would be ideal in Cali that in addition to services being added in strategic locations, patients are allowed to access services in any of the TAZs hosting them, and these services need to absorb the demand.

We suggest data updates to assess the post-COVID-19 situation, new demographics, infrastructure, and mobility.

If planning authorities must decide, we recommend adding sufficient services to cope with demand, first in the most impactful recommended location, using the July estimates, which seem to reflect typical conditions in Cali. However, a better option is to update the assessments as the COVID-19 pandemic recedes and new population and services data is available. The recommendation to add service(s) accessible to all patients in eastern Cali, near the Alirio Mora Beltrán neighbourhood, might not change. Implementing that recommendation would probably double accessibility and reduce inequities.

### Generalisability

Our findings show that data on accessibility to haemodialysis can be obtained and delivered in user-accessible formats developed with stakeholders' input; this can be done with other services for which georeferenced information is available. This can likely be achieved in other countries offering open disaggregated data of services and population data (e.g., census, service registries) and travel time big data, usually sold by private providers. This is likely the case for many cities facing significant traffic congestion in Latin America and beyond. DSTAM might be less valuable in uncongested areas, such as most rural settings, where travel-time variations are slight.

The study involved a multistakeholder, multidisciplinary group that included local data scientists, authorities and academics.^54^ It can likely be replicated with local stakeholder engagement and city resources. For services such as tertiary care emergencies accessible to the entire population in Colombia and other countries, findings can be easily generalised. More precise measurements will require added layers of information for services where access is restricted, as was the case of haemodialysis in Cali. Nevertheless, the bigger picture may remain valid, as services are needed in densely populated neglected areas.

There is room for refinement by adding layers such as the incidence of end-stage kidney disease and the availability of haemodialysis chairs. Or integrating data about provider agreements or insurance coverage distribution among populations. Still, the recommendation of our study to add services that all citizens can access in the recommended areas might hold. Repeating these studies regularly as infrastructure, traffic conditions, and population change should be part of integrating health with urban territorial planning and monitoring of health equity.^50^^,^^106^

Our findings indicate that strategically adding new services can dramatically improve accessibility and synergically advance SDG 3, SDG 10, and SDG 11 in Cali.

Studies used to rely on samples, fixed estimates, or analyses that missed the effects of the substantial impact of traffic congestion on health equity and accessibility.^23^^,^^65^^,^^107^^,^^108^ We have now tested an approach that addresses these issues. We suggest new metrics based on travel times to monitor accessibility for specific indicators, such as coverage for essential health services (3.8.1, the percentage of the population with access to essential health services) in urban areas with traffic congestion.

It also highlights that these data could promote civil society participation in urban and health services planning (SDG indicator 11.3.2). Urban planners and decision-makers can make sound decisions about the placement for healthcare facilities and discuss patient's needs and concerns about the distribution of resources.^68^^,^^109^, ^110^, ^111^ Cali's Territorial Health Plan 2020–2023 placed equitable access to health services among its strategic objectives to fulfil legal and constitutional mandates, and we present an approach to measure the fulfilment of this objective. DSTAM and predictions could inform stakeholders in shaping the 2024–2027 Territorial Health Plan implementation and monitoring.^51^ DSTAM could be used to put health equity higher on the agenda of local governments in cities with traffic congestion and advance the New Urban Agenda.^4^^,^^5^^,^^112^^,^^113^

### Limitations of the study

Variations between assessments are likely influenced by changes in travel restrictions and stay-at-home orders during the COVID-19 pandemic. It is still unknown how this influenced Google Distance Matrix algorithms.^60^^,^^114^ Empirical and anecdotal reports suggest they remained accurate.^99^

The AMORE Project quantifies the impact of traffic congestion on accessibility to haemodialysis services. It reveals the populations affected, stressing the impact of inaction over a problem that can be addressed. It does not explain the contextual issues that have perpetuated Cali's segregation of sociodemographic and ethnic groups or haemodialysis and other health services concentration along the north-south corridor (Calle 5, 1st and 6th Avenues, Fig. 1) where many of the city's best-known landmarks and higher education institutions are located.^94^^,^^95^^,^^115^

While we can broadly estimate demand for new services based on the prevalence of end-stage kidney disease and haemodialysis services, a proper analysis of demand for specific populations and sectors is unavailable. The geographic, ethnic, and socioeconomic distribution of haemodialysis patients in Cali is unknown to the research team.^48^

For predictions to be met, patients should be given access to the service and integral treatments within the shortest travel time. Our estimates do not account for health services fragmentation present in Colombia's health system.

Accessibility to services might depend on insurance affiliation, and despite the eleven haemodialysis units being concentrated in six TAZ, it might well be that service provision fragmentation (i.e., insurance restrictions) or quality issues drive patients to services further beyond those with the shortest travel times, making our findings optimistic.

This report does not include an economic analysis, but low-income families might rely on just one or two minimum wages. Frequent long trips can hurt these families by consuming most of the minimum wage in travel costs.^116^, ^117^, ^118^ Low-income families are likely to live with three or fewer minimum wages and can easily incur catastrophic health costs well above 15% of their family income.^119^^,^^120^ This study does not adjust the socioeconomic stratum for low-paid, live-in domestic workers in middle or high-income households.

The integral treatment of terminal renal failure includes other ambulatory support education for patients and carers, therapies, and training, such as exercise and physical rehabilitation or nutrition.^20^^,^^121^, ^122^, ^123^ This can be provided in the same setting as haemodialysis, but we did not assess this.

Travel time between TAZs follows diverse daily patterns and directions with commuting impacting TAZs differently. Traffic clusters were sorted in incremental order for each TAZ, and it was found that the sorting switched the order of contiguous clusters in fifteen cases. This has a minimal impact as variations between adjacent clusters are small. Poor road conditions can add to travel times and are not evenly distributed throughout the city.^124^ Infrastructure changes, for example, road enhancements and new traffic light corridors controlled by artificial intelligence, might justify reassessing travel times.

This study did not assess appointment availability in services, a refinement that could be developed for the AMORE Platform.

Future assessments could include predictions for adding services to existing infrastructure, such as public hospitals.^3^^,^^125^ We tested this for tertiary care emergency services but not for haemodialysis; no public hospitals are near the recommended locations.

This report focuses on data generation and does not measure whether stakeholders, including those contributing to the study, peruse the new data this study provides to inform the city's territorial plans, which the incumbent administration will develop in 2024. As DSTAM develops research will likely enrich knowledge about its use.^4^, ^27^, ^30^, ^31^, ^34^, ^53^, ^69^, ^78^, ^79^, ^81^, ^83^, ^84^, ^88^, ^99^, ^100^, ^101^, ^105^, ^118^, ^120^, ^125^, ^126^, ^127^ While our study raises awareness about the pervasive lack of services within a short journey of the most heavily populated areas, it does not delve into the causes behind urban segregation, migration, and other historical challenges behind the disconnect between health equity aspirations and the realities seen in the city's development. The study exposes the situation, a vision of what concerted data-driven action could achieve for Cali, and an approach that could be used for monitoring.

## Contributors

These were the author's contributions per Contributor Roles Taxonomy (CRediT)^128^: **Luis Gabriel Cuervo (LGC):** conceptualisation, data curation, formal analysis, funding acquisition, investigation, methodology, project administration, resources, software, supervision, validation, visualisation, led the writing of the original draft, reviewed and edited the draft and is the corresponding author; **Carmen Juliana Villamizar (CJV):** data curation, project administration, supervision, contributed to the original draft, reviewed and edited the draft; **Lyda Osorio (LO):** conceptualisation, methodology, reviewed and edited the draft; **María Beatriz Ospina (MBO):** reviewed and edited the draft; **Diana E. Cuervo (DECD):** reviewed and edited the draft; **Daniel Cuervo (DC):** conceptualisation, formal analysis, methodology, resources, software, validation, visualisation, reviewed and edited the draft; **María O. Bula (MB):** investigation, reviewed and edited the draft; **Pablo Zapata (PZ):** software, validation, visualisation, reviewed and edited the draft; **Nancy J. Owens (NJO):** reviewed and edited the draft; **Janet Hatcher-Roberts (JHR):** reviewed and edited the draft; **Edith Alejandra Martín (EAM):** reviewed and edited the draft; **Felipe Piquero (FP):** reviewed and edited the draft; **Luis Fernando Pinilla (LFP):** conceptualisation, data curation, software; **Eliana Martínez-Herrera (EMH):** investigation, supervision, visualisation, reviewed and edited the draft; **Ciro Jaramillo (CJ):** conceptualisation, data curation, formal analysis, investigation, methodology, supervision, validation, reviewed and edited the draft. All authors approved the final draft for publication and agreed to be accountable for all aspects of this report. LGC, CJV, and CJM directly accessed and verified the data; all authors were given access to the web-based AMORE platform when reviewing the draft manuscript. DCA, LFP, LGC and PZ verified the AMORE Platform data validity.

**Dedication:** This manuscript is dedicated to the late Dr. Arnoldo Bromet Schumm, a pioneer of family medicine in Cali, Colombia. In October 2021, Dr. Bromet Schumm agreed to join the AMORE Project, offering his insights as a haemodialysis patient, a healthcare provider, and an organiser of health services. He died in January 2022.

## Data sharing statement

The travel-time datasets used by the AMORE web-based platform are available from OpenScience [July DOI 10.17605/OSF.IO/XDA87 and November DOI 10.17605/OSF.IO/FMJ2X], and the AMORE Platform's interactive interface used for this report is publicly available from the project's websites. Open data sources used in this study are listed in the Study Design and the cited protocol. Readers can interact with changing parameters and testing assumptions. These references include links to the website, tutorials, databases, blogs, and presentations about the AMORE Project.^54^^,^^55^

The draft submitted for publication was also approved by the following members of the AMORE Project Collaboration who agreed to be accountable for all aspects of this report: Freddy Enrique Agredo Lemos, Germán Ávila Rodríguez, Alberto Concha-Eastman, Oscar H. Franco, Crhistian Camilo García Altamirano, María Fernanda Merino Juárez, Gynna Millán, Jackeline Murillo-Hoyos, Gabriel D. Paredes-Zapata, Oscar Rojas R, and María Fernanda Tobar-Blandón. Additional details are provided as a supplemental file.

## Editor note

The Lancet Group takes a neutral position with respect to territorial claims in published maps and institutional affiliations.

## Declaration of interests

All authors and collaborators completed the ICMJE uniform disclosure form and declared no financial support from any organisation for the submitted work. IQuartil SAS provided technical support to develop the AMORE Platform and was subsidised for consulting services by LGC. For the AMORE Platform development, PZ and LFP received consulting fees and time from IQuartil SAS. DC is a partner at IQuartil SAS and a sibling to LGC. LGC contributed to this work in his personal capacity and time. CJ and EMH are LGC's thesis directors. EAM disclosed a fiduciary role with the Consul World Transplant Games Federation. Universidad del Valle supports LO's academic contribution. LGC and CJV contributed personal time to Driving for Equity, a proposal aligned with the AMORE Project and a finalist at WHO's 2023 LEAD Innovation Challenge. All other authors have nothing to declare.

## Figures and Tables

**Supplemental File 1:**
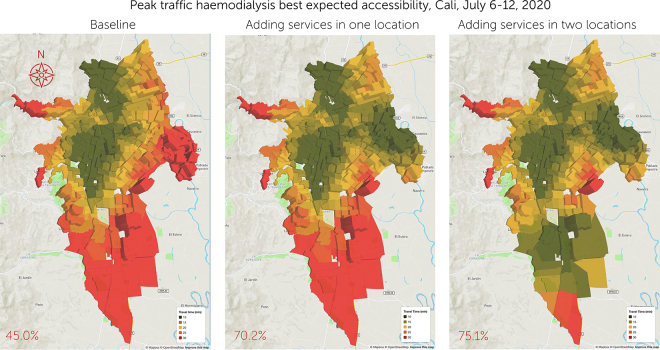


## Appendix A Supplementary data

Translated Abstract

20230509 Infografi_a 2 y mapas haemodialisis Julio 2020

20230509 Infographic 2 and maps haemodialysis July 2020

Cuervo-2022-Dynamic-geographical-accessibility-Protocol

Authors and Contributors

xda87 July data CSV.json

xda87 Nov data CSV.json

## Footnotes

q The AMORE Platform can be accessed from www.iquartil.net/project-amore-ingles
