# Addendum to “*Dynamic measurements of geographical accessibility considering traffic congestion using open data: a cross-sectional assessment for haemodialysis services in Cali, Colombia*” [The Lancet Regional Health – Americas 2024; VOLUME 34, 100722, Published: May 3, 2024]

**DOI:** 10.1016/j.lana.2024.100797

**Published:** 2024-06-03

**Authors:** Luis Gabriel Cuervo, Carmen Juliana Villamizar, Lyda Osorio, María Beatriz Ospina, Diana E. Cuervo, Daniel Cuervo, María O. Bula, Pablo Zapata, Nancy J. Owens, Janet Hatcher-Roberts, Edith Alejandra Martín, Felipe Piquero, Luis Fernando Pinilla, Eliana Martínez-Herrera, Ciro Jaramillo, AMORE Project Collaboration

**Affiliations:** a Universitat Autònoma de Barcelona, Barcelona, Spain; b Johns Hopkins Bloomberg School of Public Health, Baltimore, MD, USA; c School of Public Health, Universidad del Valle, Cali, Colombia; d Faculty of Health Sciences, Queen's University, Kingston, ON, Canada; e National Disability Board of Colombia, Bogotá, Colombia; f IQuartil SAS, Bogotá, Colombia; g Independent Researcher, Bogotá, Colombia; h Independent Content and Communications Consultant, Fairfax, VA, USA; i School of Epidemiology and Public Health in the Faculty of Medicine, and Bruyère Research Institute, University of Ottawa, Ottawa, ON, Canada; j Colombian Association of Transplanted Athletes, Bogotá, Colombia; k Patient Advocate and Author of an Autopathography, Bogotá, Colombia; l Universidad de la Sabana, Campus del Puente del Común, Chía, Cundinamarca, Colombia; m National Faculty of Public Health, Universidad de Antioquia, Medellín, Colombia; n School of Civil and Geomatic Engineering of the Universidad del Valle, Cali, Colombia; o JHU-UPF Public Policy Center, Departament de Ciències Polítiques i Socials, Universitat Pompeu Fabra, Barcelona School of Management, Barcelona, Spain

The authors wish to include the following description and link as a supplementary file:

The Laboratorio de Barrios Populares PopuLab at the Universidad del Valle in Cali, Colombia, is an innovative space dedicated to studying and applying ideas surrounding self-built neighbourhoods and urban development. Its primary goal is to bridge the gap between the aspirations of communities in popular neighbourhoods and the urban planning proposals from local governments. PopuLab has authorized the use of its video, ‘Video participativo: movilidad comuna 18 Cali, Colombia’^(16)^ to enhance this article with testimonies from users of health services. The video has close captioning with automatic multilingual translation.

https://youtu.be/4fUgx7osKfw?si=KA_t7wQrpK1Qpiv6.

https://www.youtube.com/embed/4fUgx7osKfw?si=KA_t7wQrpK1Qpiv6.

