# Supplementary material for: Dynamic measurements of geographical accessibility considering traffic congestion using open data: a cross-sectional assessment for haemodialysis services in Cali, Colombia
Source: Lancet Reg Health Am. 2024 May 3;34:100752. doi: 10.1016/j.lana.2024.100752 (PMC11087994; doi:10.1016/j.lana.2024.100752)
Supplement: Translated Abstract [file mmc1.docx]

# **SUMMARY**

**Background:** Many cities with traffic congestion lack accessibility assessments accounting for traffic congestion and equity considerations but have disaggregated georeferenced municipal-level open data on health services, populations, and travel times big data. We convened a multistakeholder intersectoral collaborative group that developed a digital, web-based platform integrating open and big data to derive dynamic spatial-temporal accessibility measurements (DSTAM) for haemodialysis services. We worked with stakeholders and data scientists and considered people’s places of residence, service locations, and travel time to the service with the shortest travel time. Additionally, we predicted the impacts of strategically introducing haemodialysis services where they optimise accessibility.

**Methods:** Cross-sectional analyses of DSTAM, accounting for traffic congestion, were conducted using a web-based platform. This platform integrated traffic analysis zones, public census and health services datasets, and Google Distance Matrix API travel-time data. Predictive and prescriptive analytics identified optimal locations for new haemodialysis services and estimated improvements. Primary outcomes included the percentage of residents within a 20-minute car drive of a haemodialysis service during peak and free-flow traffic congestion. Secondary outcomes focused on optimal locations to maximise accessibility with new services and potential improvements. Findings were disaggregated by sociodemographic characteristics, providing an equity perspective. The study in Cali, Colombia, used geographic and disaggregated sociodemographic data from the adjusted 2018 Colombian census. Predicted travel times were obtained for two weeks in 2020.

**Findings:** There were substantial traffic variations. Congestion reduced accessibility, especially among marginalised groups. For 6-12 July, free-flow and peak-traffic accessibility rates were 95.2% and 45.0%, respectively. For 23-29 November, free-flow and peak traffic accessibility rates were 89.1% and 69.7%. The locations where new services would optimise accessibility had slight variations and would notably enhance accessibility and health equity.

**Interpretation:** Establishing haemodialysis services in targeted areas has significant potential benefits. By increasing accessibility, it would enhance urban health and equity.

**ESPAÑOL**

**Antecedentes:** Muchas ciudades congestionas carecen de evaluaciones de accesibilidad que contemplen el tráfico y la equidad, pero cuentan con datos abiertos georreferenciados de los servicios de salud municipales y la población, y macrodatos de los tiempos de traslado. Convocamos a un grupo colaborativo intersectorial con diversos actores interesados que desarrollaron una plataforma digital basada en la web, que integró datos abiertos y macrodatos para derivar mediciones dinámicas de accesibilidad espaciotemporal (DSTAM) para los servicios de hemodiálisis. Trabajamos con las partes interesadas y científicos de datos, considerando el lugar de residencia de las personas, las ubicaciones de los servicios y el tiempo de traslado hasta el servicio con el menor tiempo de viaje. También predijimos impactos resultantes de ubicar nuevos servicios de hemodiálisis estratégicamente para que optimicen la accesibilidad.

**Métodos:** Se realizaron análisis transversales de DSTAM, teniendo en cuenta la congestión del tráfico, utilizando una plataforma basada en la web. Esta plataforma integró zonas de análisis de tráfico, conjuntos de datos de censos públicos y servicios de salud, y datos de tiempo de viaje de la API de Google Distance Matrix. Los análisis predictivos y prescriptivos identificaron las ubicaciones óptimas para los nuevos servicios de hemodiálisis y estimaron las mejoras. Los resultados primarios incluyeron el porcentaje de residentes que se encontraban a menos de 20 minutos en automóvil de un servicio de hemodiálisis durante las horas pico y la congestión del tráfico de flujo libre. Los resultados secundarios se centraron en ubicaciones óptimas para maximizar la accesibilidad con nuevos servicios y posibles mejoras. Los resultados se desagregaron por características sociodemográficas, lo que proporciona una perspectiva de equidad. El estudio en Cali, Colombia, utilizó datos geográficos y sociodemográficos desagregados del censo colombiano de 2018 ajustado. Los tiempos de viaje previstos se obtuvieron para dos semanas en 2020.

**Resultados:** Hubo variaciones sustanciales en el tráfico. La congestión redujo la accesibilidad, especialmente entre los grupos marginados. Del 6 al 12 de julio, las tasas de accesibilidad de flujo libre y de tráfico pico fueron del 95,2% y del 45,0%, respectivamente. Del 23 al 29 de noviembre, las tasas de accesibilidad fueron del 89,1% y el 69,7% para flujo libre y tráfico pico, respectivamente. La variación de las ubicaciones que optimizan la accesibilidad agregando nuevos servicios serían menores y habría mejoras notables en la accesibilidad y la equidad en salud.

**Interpretación:** El establecimiento de servicios de hemodiálisis en áreas específicas tiene importantes beneficios potenciales. Al aumentar la accesibilidad, mejoraría la salud y la equidad urbanas.

**PORTUGUÊS (Por favor, revise)**

**Contexto:** Muitas cidades congestionadas carecem de avaliações de acessibilidade que comsiderem o trânsito e a equidade, mas têm dados abertos georreferenciados sobre os serviços municipais de saúde e a população, e grandes dados sobre os tempos de viagem. Reunimos um grupo colaborativo intersetorial com diversas partes interessadas que desenvolveram uma plataforma digital baseada na web que integrou dados abertos e big data para obtener medições dinâmicas de acessibilidade espaço-temporal (DSTAM) para serviços de hemodiálise. Trabalhamos com partes interessadas e cientistas de dados, considerando onde as pessoas vivem, locais de serviço e tempo de deslocamento para o serviço com o menor tempo de viagem. Também foram previstos impactos decorrentes da localização estratégica de novos serviços de hemodiálise para otimizar a acessibilidade.

**Métodos:** Análises transversais do DSTAM, levando em consideração o congestionamento do tráfego, foram realizadas em uma plataforma web. Essa plataforma integrou zonas de análise de tráfego, conjuntos de dados de censos públicos e serviços de saúde e dados de tempo de viagem da API Google Distance Matrix. Análises preditivas e prescritivas identificaram locais ideais para novos serviços de hemodiálise e estimaram melhorias. Os desfechos primários incluíram a porcentagem de residentes que estavam a menos de 20 minutos de carro de um serviço de hemodiálise durante os horários de congestionamento de tráfego de pico e de trânsito livre. Os resultados secundários centraram-se em localizações ideais para maximizar a acessibilidade com novos serviços e potenciais melhorias. Os resultados foram desagregados por características sociodemográficas, proporcionando uma perspectiva de equidade. O estudo em Cali, Colômbia, usou dados geográficos e sociodemográficos desagregados do censo colombiano ajustado de 2018. Os tempos de viagem previstos foram obtidos para duas semanas em 2020.

**Resultados:** Houve variações substanciais no tráfego. O congestionamento reduziu a acessibilidade, especialmente entre grupos marginalizados. De 6 a 12 de julho, as taxas de fluxo livre e de acessibilidade ao tráfego de pico foram de 95,2% e 45,0%, respectivamente. De 23 a 29 de novembro, as taxas de acessibilidade foram de 89,1% e 69,7% para fluxo livre e tráfego de pico, respectivamente. A variação de locais que otimizam a acessibilidade pela adição de novos serviços seria menor, e haveria melhorias notáveis na acessibilidade e equidade em saúde.

**Interpretação:** O estabelecimento de serviços de hemodiálise em áreas específicas traz benefícios potenciais significativos. Ao aumentar a acessibilidade, melhoraria a saúde urbana e a equidade.
