# Supplementary material for: Dynamic measurements of geographical accessibility considering traffic congestion using open data: a cross-sectional assessment for haemodialysis services in Cali, Colombia
Source: Lancet Reg Health Am. 2024 May 3;34:100752. doi: 10.1016/j.lana.2024.100752 (PMC11087994; doi:10.1016/j.lana.2024.100752)
Supplement: 20230509 Infografi_a 2 y mapas haemodialisis Julio 2020 [file mmc2.pdf]

Proyecto AMORE

POSIBILITANDO LA PLANIFICACIÓN URBANA Y DE SERVICIOS DE SALUD BASADA EN DATOS

Uso de datos abiertos para revelar los vínculos entre la congestión del tráfico, la accesibilidad y la equidad

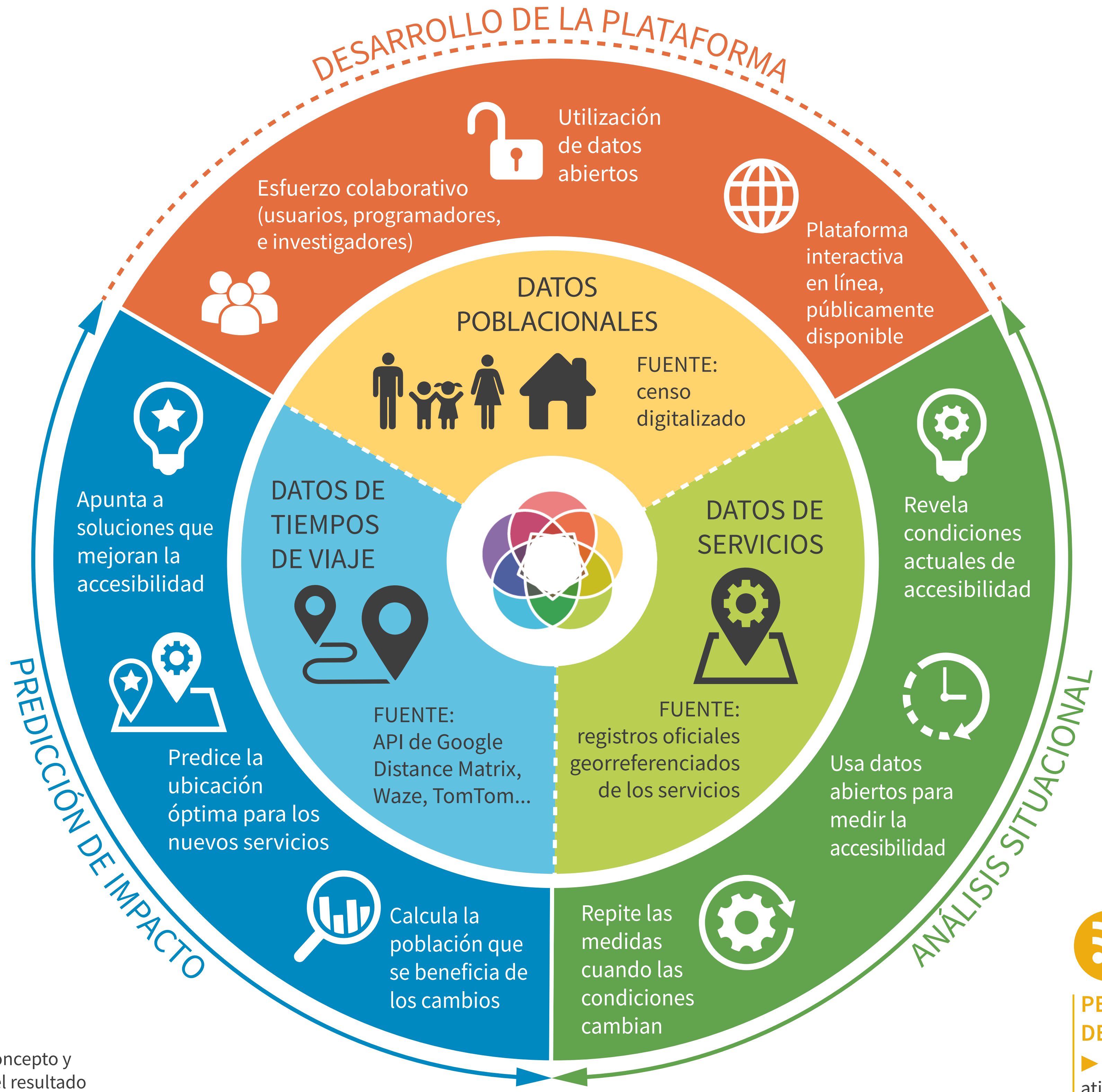

PROCESO INCLUSIVO

- Este nuevo concepto y plataforma son el resultado de una co-creación que involucró a partes interesadas y científico de datos.
- Las partes interesadas participantes incluyeron usuarios y proveedores de servicios de salud, funcionarios guberna-mentales, comunidades y la academia.
- El proceso y la plataforma buscan la apropiación social del conocimiento.
- Los datos se presentan en mapas y estadísticas descriptivas para que las partes interesadas no especializadas puedan interpretarlos y comunicarlos.
- El enfoque fue probado en un proyecto de investigación colaborativo.

EVALUACIÓN DINÁMICA

- La plataforma ofrece medidas espacio-temporales dinámicas de accesibilidad, indicando los tiempos de viaje al servicio y el viaje más corto para los niveles de congestión del tráfico.
- Muestra las poblaciones y sectores dentro de un umbral de tiempo de viaje.
- Predice cambios de accesibilidad al agregar servicios en uno o dos sectores óptimos.

PROYECCIONES REALISTAS

- La plataforma identifica ubicaciones para nuevos servicios para optimizar la accesibilidad.
- El concepto ha sido probado en Cali, Colombia (2.25M) usando tres servicios de salud que salvan vidas: hemodiálisis, radiotera-pia y emergencias de alta complejidad.

MEDIDAS PRECISAS

- La plataforma toma muestras de millones de mediciones de tiempos de viaje entre las zonas residenciales y las de los servicios de salud.
- Utiliza datos de población y vivienda anonimizados y georreferenciados del censo con granularidad que permite evaluaciones precisas.

ENFOQUE PRÁCTICO

- La plataforma aprovecha datos abiertos fácilmente disponibles (censo, ubicación del servicio) y macrodatos (tiempos de viaje).
- Puede actualizarse a medida que cambian las condiciones (por ejemplo, demografía, infraestructura, tráfico).
- Apoya la planificación y el monitoreo continuo de la accesibilidad.
- Puede informar análisis de accesibilidad, priorización, seguimiento y evaluaciones de impacto.

PERSPECTIVA DE EQUIDAD

- El concepto atiende las lagunas de evidencia y revela desigualdades ocultas a simple vista.
- Los datos y análisis se desglosan por características sociodemográficas, proporcionando una perspectiva de equidad. Por ejemplo, mostrando diferencias por edad, lugar de residencia, identifica-ción sexual, etnia, nivel más alto de educación alcanzado, estado civil o estrato económico de la unidad de vivienda.
- Revela en qué medida la congestión del tráfico reduce la accesibilidad.

CAPACIDAD EXPANSIVA

- El proceso participativo y la plataforma se pueden adaptar y probar para otros servicios, sectores y partes interesadas.
- Potencial para la integración de capas de datos adicionales (v.g., aseguradores, disponibilidad de servicios).
- El proyecto desarrolló un concepto emergente utilizando un enfoque participativo.
- Señala soluciones específicas que predicen el impacto y que ofrecen medios para la rendición de cuentas.

Proyecto AMORE

Proyecto AMORE

Accesibilidad geográfica en automóvil a hemodiálisis
Horas de tráfico pico
6-12 de julio de 2020, Cali, Colombia

Fuente: doi 10.2139/ssrn.4299562

@Proyecto_AMORE

LinkedIn: **@AMORE Project**

1. Línea de base

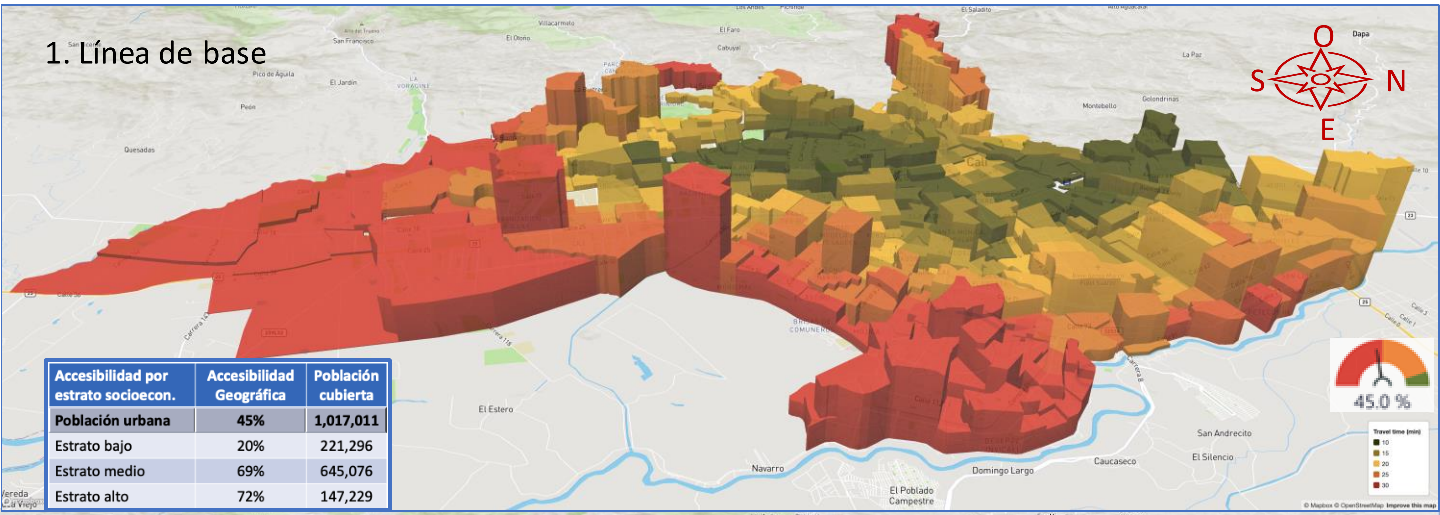

Accesibilidad por estrato socioecon.	Accesibilidad Geográfica	Población cubierta
**Población urbana**	**45%**	**1,017,011**
Estrato bajo	20%	221,296
Estrato medio	69%	645,076
Estrato alto	72%	147,229

2. Predicción añadiendo servicios en el sector oriental Alirio Mora Beltrán

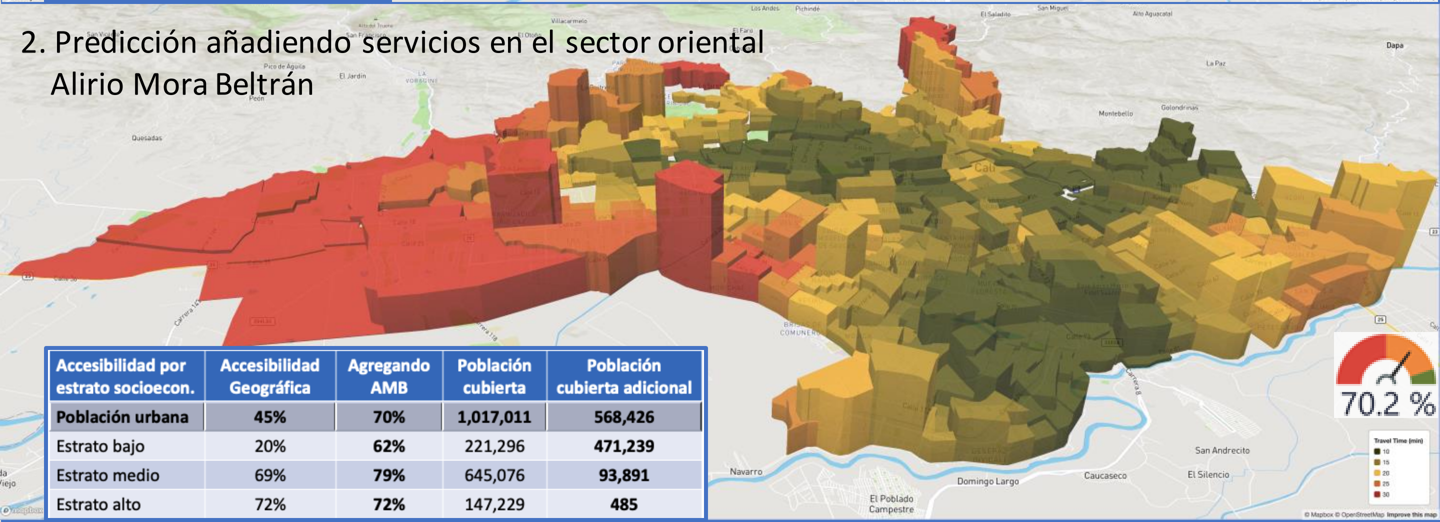

Accesibilidad por estrato socioecon.	Accesibilidad Geográfica	Agregando AMB	Población cubierta	Población cubierta adicional
**Población urbana**	**45%**	**70%**	**1,017,011**	**568,426**
Estrato bajo	20%	**62%**	221,296	**471,239**
Estrato medio	69%	**79%**	645,076	**93,891**
Estrato alto	72%	**72%**	147,229	**485**

3. Predicción añadiendo servicios en Alirio Mora Beltrán y en Parcelaciones del Pance

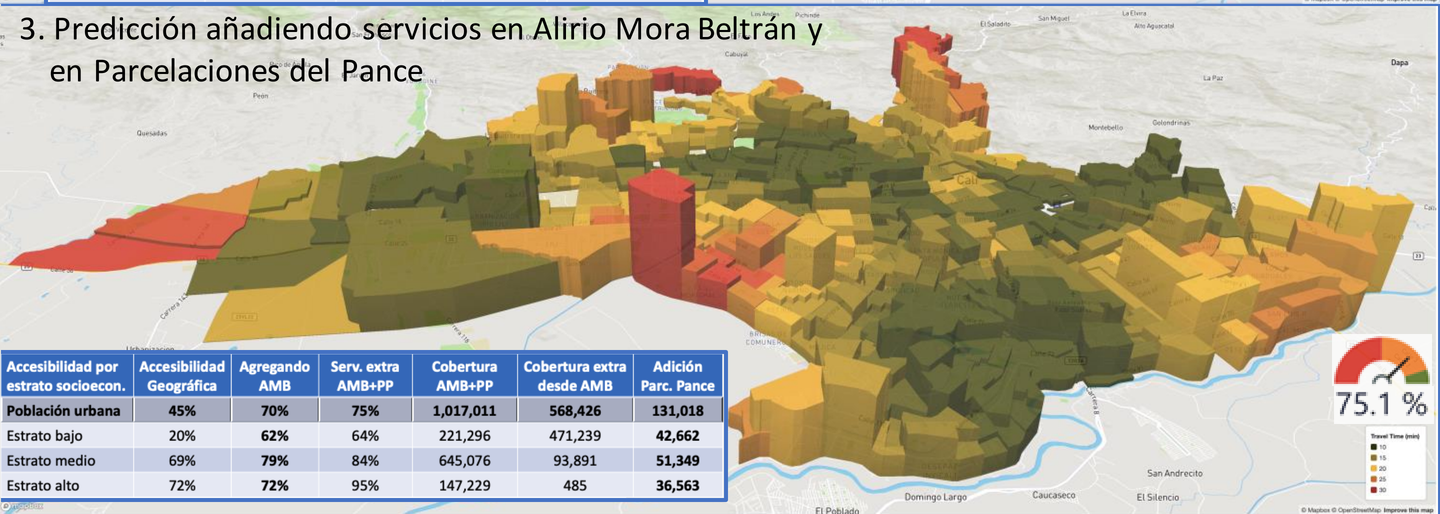

Accesibilidad por estrato socioecon.	Accesibilidad Geográfica	Agregando AMB	Serv. extra AMB+PP	Cobertura AMB+PP	Cobertura extra desde AMB	Adición Parc. Pance
**Población urbana**	**45%**	**70%**	**75%**	**1,017,011**	**568,426**	**131,018**
Estrato bajo	20%	**62%**	64%	221,296	471,239	**42,662**
Estrato medio	69%	**79%**	84%	645,076	93,891	**51,349**
Estrato alto	72%	**72%**	95%	147,229	485	**36,563**
