# Supplementary material for: Dynamic measurements of geographical accessibility considering traffic congestion using open data: a cross-sectional assessment for haemodialysis services in Cali, Colombia
Source: Lancet Reg Health Am. 2024 May 3;34:100752. doi: 10.1016/j.lana.2024.100752 (PMC11087994; doi:10.1016/j.lana.2024.100752)
Supplement: 20230509 Infographic 2 and maps haemodialysis July 2020 [file mmc3.pdf]

AMORE Project

ENABLING DATA-DRIVEN URBAN AND HEALTH SERVICES PLANNING

Use of open data to reveal the links between traffic congestion, accessibility, and equity

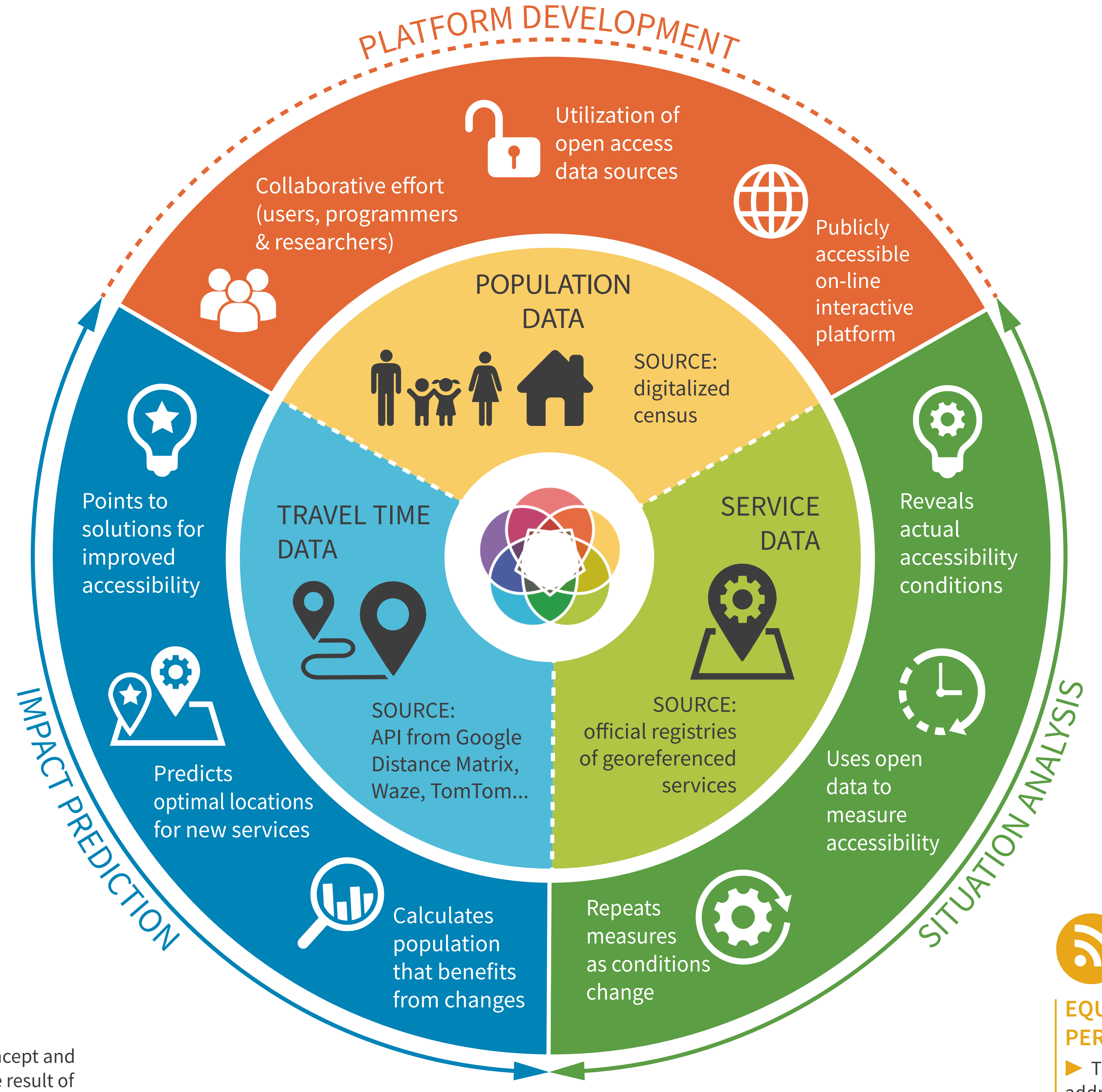

INCLUSIVE PROCESS

- This new concept and platform are the result of a cocreation involving stakeholders and data scientist.
- Stakeholders included health services users, providers, government officials, communities, and academia.
- The concept and platform seek the social appropriation of knowledge.
- Data is displayed in maps and descriptive statistics for non-specialized stakeholders to interpret and communicate.
- The approach was tested in a collaborative research project.

DYNAMIC ASSESSMENT

- The platform delivers dynamic spatial-temporal measurements of accessibility, indicating travel-times to the service and the shortest journey for the traffic congestion levels.
- Shows the populations and sectors within a travel time threshold.
- Predicts accessibility changes for adding services in one or two optimal sectors.

REALISTIC PROJECTIONS

- The platform identifies locations for new services to optimize accessibility.
- The concept was tested in Cali, Colombia (2.25m) using three life-saving health services: haemodialysis, radiotherapy, and tertiary-care emergencies.

ACCURATE MEASURES

- The platform samples millions of travel time measurements between residential zones and those of health services.
- Uses anonymized and georeferenced population and housing data from the census with granularity that allows for accurate assessments.

PRACTICAL APPROACH

- The platform leverages readily available open data (census, service location) and big data (travel times).
- Can be updated as conditions change (e.g., demographics, infrastructure, traffic).
- Supports continuous planning and monitoring of accessibility.
- It can be used to inform accessibility analyses, prioritization, follow-up, and impact assessments.

EQUITY PERSPECTIVE

- The concept addresses evidence gaps and reveals inequities hidden in plain sight.
- Data and analyses are disaggregated by sociodemographic characteristics, providing an equity perspective. For example, showing differences by age, place of residence, sexual identification, ethnicity, highest level of educa-tion attained, marital status, or economic stratum of the housing unit.
- Reveals the extent by which traffic congestion reduces accessibility.

EXPANSIVE CAPACITY

- The participatory process and the platform can be adapted and tested for other services, sectors, and stakeholders.
- Potential for integration of additional data layers (e.g., insurance provider, availability of services).
- The project developed an emerging concept using a participatory approach.
- Points at specific solutions predicting their impact and offering means for accountability.

AMORE Project

Geographical Accessibility by Car to Haemodialysis
Peak Traffic Hours
6-12 July 2020, Cali, Colombia

AMORE Project

Source: doi 10.2139/ssrn.4299562

@Proyecto_AMORE
LinkedIn: @AMORE Project

1. Baseline

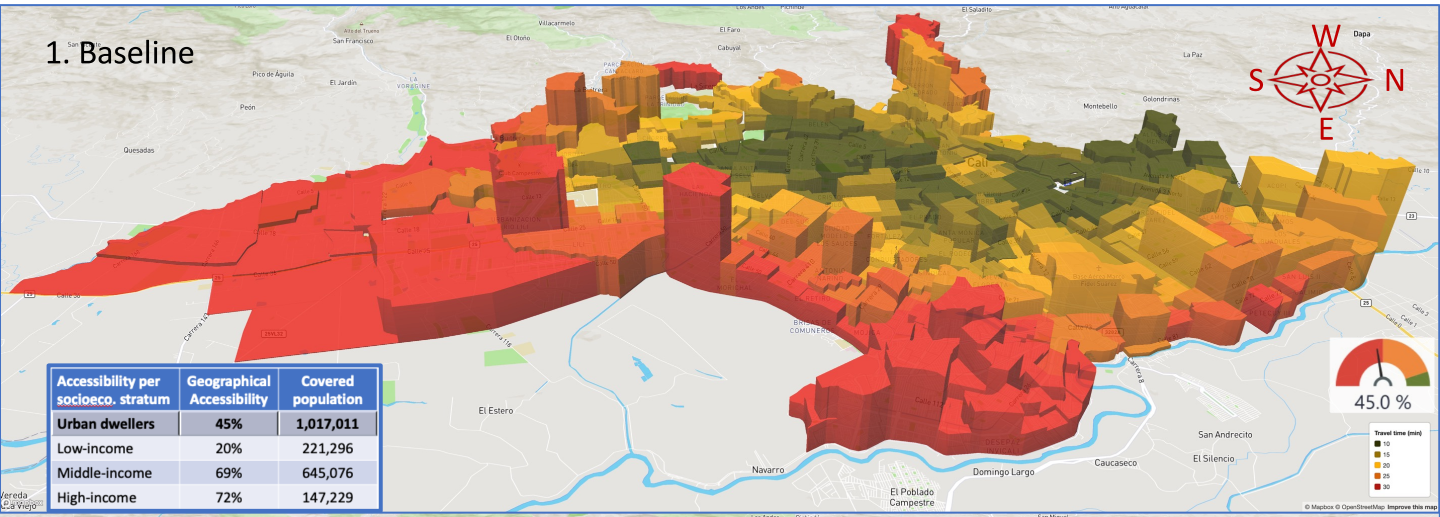

Accessibility per socioeco. stratum	Geographical Accessibility	Covered population
**Urban dwellers**	**45%**	**1,017,011**
Low-income	20%	221,296
Middle-income	69%	645,076
High-income	72%	147,229

2. Prediction if adding services in the eastern sector of Alirio Mora Beltrán

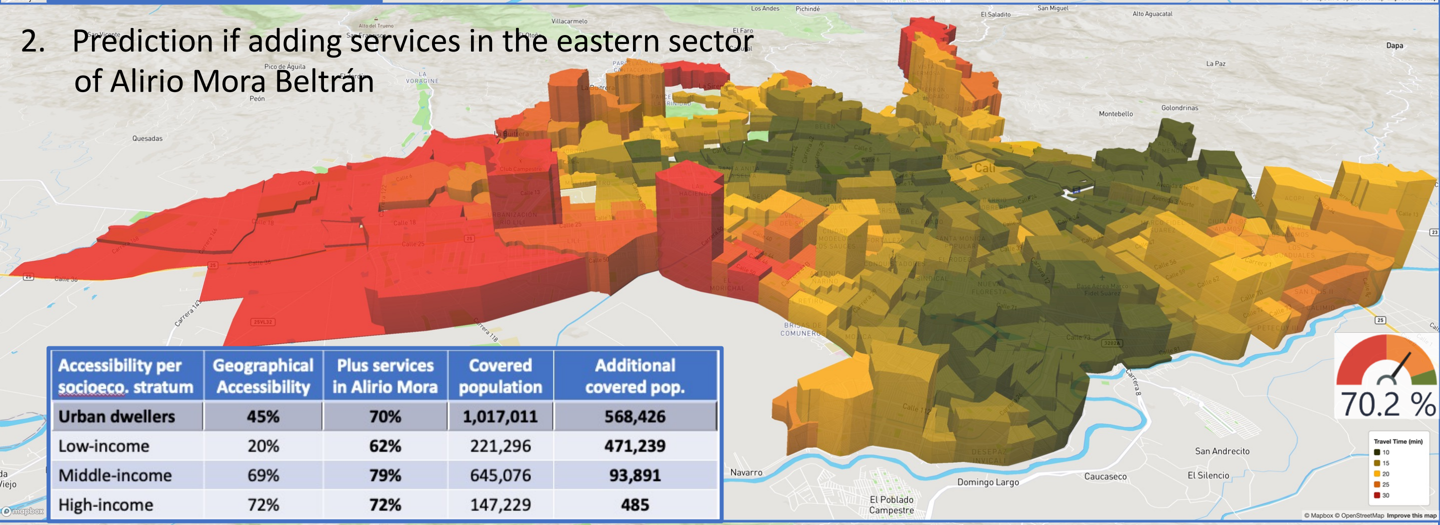

Accessibility per socioeco. stratum	Geographical Accessibility	Plus services in Alirio Mora	Covered population	Additional covered pop.
**Urban dwellers**	**45%**	**70%**	**1,017,011**	**568,426**
Low-income	20%	**62%**	221,296	**471,239**
Middle-income	69%	**79%**	645,076	**93,891**
High-income	72%	**72%**	147,229	**485**

3. Prediction if adding services in Alirio Mora Beltrán and Parcelaciones del Pance

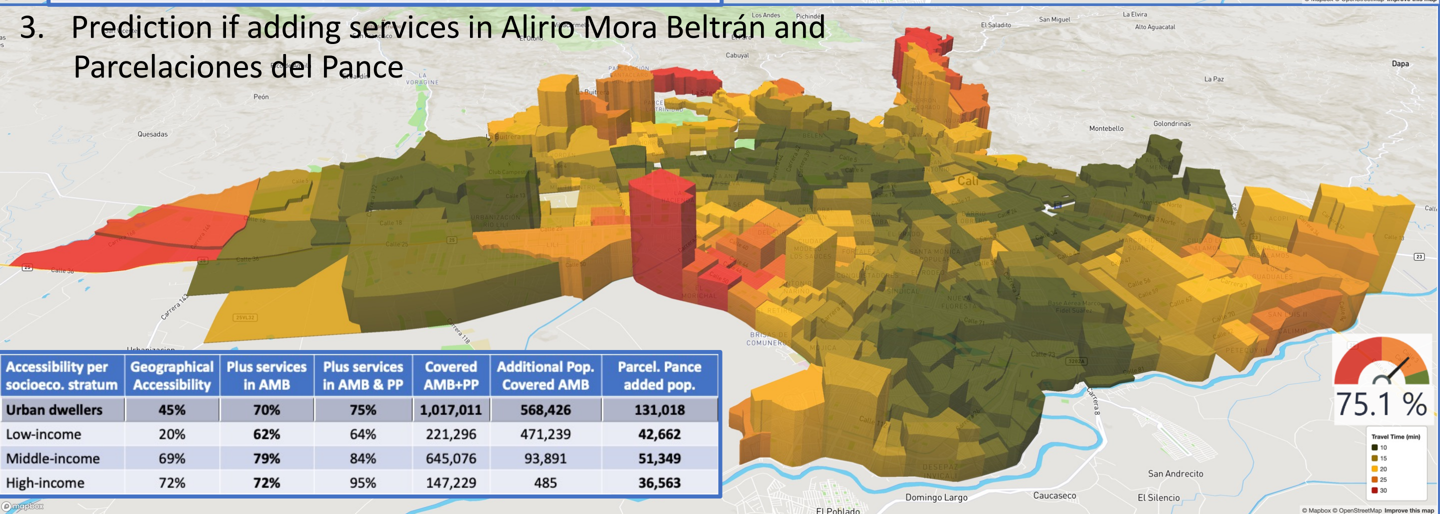

Accessibility per socioeco. stratum	Geographical Accessibility	Plus services in AMB	Plus services in AMB & PP	Covered AMB+PP	Additional Pop. Covered AMB	Parcel. Pance added pop.
**Urban dwellers**	**45%**	**70%**	**75%**	**1,017,011**	**568,426**	**131,018**
Low-income	20%	**62%**	64%	221,296	471,239	**42,662**
Middle-income	69%	**79%**	84%	645,076	93,891	**51,349**
High-income	72%	**72%**	95%	147,229	485	**36,563**
