# Supplementary material for: Dynamic measurements of geographical accessibility considering traffic congestion using open data: a cross-sectional assessment for haemodialysis services in Cali, Colombia
Source: Lancet Reg Health Am. 2024 May 3;34:100752. doi: 10.1016/j.lana.2024.100752 (PMC11087994; doi:10.1016/j.lana.2024.100752)
Supplement: Cuervo-2022-Dynamic-geographical-accessibility-Protocol [file mmc4.pdf]

Check for updates

STUDY PROTOCOL

# Dynamic geographical accessibility assessments to improve health equity: protocol for a test case in Cali, Colombia [version 1; peer review: 2 approved]

Luis Gabriel Cuervo [1], Ciro Jaramillo [2], Daniel Cuervo[3], Eliana Martínez-Herrera[4], Janet Hatcher-Roberts[5], Luis Fernando Pinilla[6], María Olga Bula[7], Lyda Osorio[8], Pablo Zapata[3], Felipe Piquero Villegas[9], Maria Beatriz Ospina[10], Carmen Juliana Villamizar[11]

[1]Department of Paediatrics, Obstetrics & Gynaecology and Preventative Medicine, Universitat Autònoma de Barcelona, Barcelona, Catalonia, Spain
[2]School of Civil and Geomatic Engineering, Universidad del Valle, Cali, Valle del Cauca, Colombia
[3]IQuartil SAS, Bogotá D.C., 110111-300, Colombia
[4]Facultad Nacional de Salud Pública, Universidad de Antioquia, Medellín, Antioquia, Colombia
[5]WHO Collaborating Centre for Knowledge Translation and Health Technology Assessment for Health Equity, Bruyère Research Institute, University of Ottawa, Ottawa, Ontario, K1R6M1, Canada
[6]Universidad de la Sabana, Bogotá D.C., Colombia
[7]Egis Consulting, Bogotá D.C., Colombia
[8]School of Public Health, Universidad del Valle, Cali, Valle del Cauca, Colombia
[9]Author of patobiography, Independent Patient Representative, Bogotá, D.C., Colombia
[10]Department of Public Health Sciences, Faculty of Health Sciences, Queen's University, Kingston, ON, K7L 3N6, Canada
[11]Johns Hopkins Bloomberg School of Public Health, Baltimore, Maryland, USA

**v1** **First published:** 28 Nov 2022, **11**:1394
https://doi.org/10.12688/f1000research.127294.1

**Latest published:** 28 Nov 2022, **11**:1394
https://doi.org/10.12688/f1000research.127294.1

## Abstract

This protocol proposes an approach to assessing the place of residence as a spatial determinant of health in cities where traffic congestion might impact health services accessibility. The study provides dynamic travel times presenting data in ways that help shape decisions and spur action by diverse stakeholders and sectors.

Equity assessments in geographical accessibility to health services typically rely on static metrics, such as distance or average travel times. This new approach uses dynamic spatial accessibility measures providing travel times from the place of residence to the health service with the shortest journey time. It will show the interplay between traffic congestion, accessibility, and health equity and should be used to inform urban and health services monitoring and planning.

Available digitised data enable efficient and accurate accessibility measurements for urban areas using publicly available sources and provide disaggregated sociodemographic information and an equity

**Open Peer Review**

**Approval Status** ✔ ✔

| | 1 | 2 |
|---|---|---|
| **version 1** 28 Nov 2022 | ✔ view | ✔ view |

1. **Airton Tetelbom Stein**, Federal University of Health Science of Porto Alegre, Porto Alegre, Brazil
Grupo Hospitalar Conceição, Porto Alegre, Brazil
**Rita Mattiello**, Universidade Federal do Rio Grande do Sul, Porto Alegre, Brazil

2. **Luiz Galvao**, Oswaldo Cruz Foundation, Rio de Janeiro, Brazil

perspective.

Test cases are done for urgent and frequent care (i.e., repeated ambulatory care). Situational analyses will be done with cross-sectional urban assessments; estimated potential improvements will be made for one or two new services, and findings will inform recommendations and future studies.

This study will use visualisations and descriptive statistics to allow non-specialized stakeholders to understand the effects of accessibility on populations and health equity. This includes “time-to-destination” metrics or the proportion of the people that can reach a service by car within a given travel time threshold from the place of residence.

The study is part of the AMORE Collaborative Project, in which a diverse group of stakeholders seeks to address equity for accessibility to essential health services, including health service users and providers, authorities, and community members, including academia.

**Keywords**
Health services accessibility, City planning, Urban health, Health inequality monitoring, Spatial Analysis, Residence characteristics

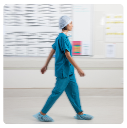

This article is included in the Health Services gateway.

Any reports and responses or comments on the article can be found at the end of the article.

**Corresponding author:** Luis Gabriel Cuervo (LuisGabriel.Cuervo@autonoma.cat)

**Author roles: Cuervo LG**: Conceptualization, Data Curation, Formal Analysis, Funding Acquisition, Investigation, Methodology, Project Administration, Resources, Supervision, Validation, Visualization, Writing – Original Draft Preparation; **Jaramillo C**: Conceptualization, Data Curation, Formal Analysis, Investigation, Methodology, Supervision, Validation, Writing – Review & Editing; **Cuervo D**: Conceptualization, Data Curation, Formal Analysis, Investigation, Methodology, Resources, Software, Supervision, Validation, Visualization, Writing – Review & Editing; **Martínez-Herrera E**: Formal Analysis, Investigation, Methodology, Supervision, Validation, Writing – Review & Editing; **Hatcher-Roberts J**: Formal Analysis, Resources, Validation, Writing – Review & Editing; **Pinilla LF**: Formal Analysis, Investigation, Resources, Software, Validation, Visualization, Writing – Review & Editing; **Bula MO**: Formal Analysis, Validation, Writing – Review & Editing; **Osorio L**: Formal Analysis, Validation, Writing – Review & Editing; **Zapata P**: Software, Visualization, Writing – Review & Editing; **Piquero Villegas F**: Resources, Writing – Review & Editing; **Ospina MB**: Conceptualization, Resources, Writing – Review & Editing; **Villamizar CJ**: Conceptualization, Project Administration, Validation, Visualization, Writing – Review & Editing

**Competing interests:** LGC is contributing his time in his personal capacity and as part of his part-time doctoral studies. His contributions and reports do not necessarily reflect the policies or decisions of his employer, the Pan American Health Organization (PAHO/WHO). IQuartil SAS was commissioned to develop the backend and front end of the AMORE Platform and to host the platform. DCA was part of Team 33, where he led the team in developing the prototype of the AMORE Platform in coordination with the principal investigator. DCA is also a partner in IQuartil SAS and guided the company's technical support and coordination with the principal investigator. LFP worked with IQuartil SAS until March 2021.

**Grant information:** IQuartil SAS received consulting fees to support the development of the advanced prototype of the AMORE Platform. The prototype of the AMORE Platform was developed as part of a collaboration between the principal investigator and Team33 during the Data Science for All (DS4A) training. No grants have been secured to support this project that the principal investigator has financed. Subsequent developments of the AMORE platform have not been considered part of the objectives or reach of the AMORE project.

**Copyright:** © 2022 Cuervo LG *et al*. This is an open access article distributed under the terms of the Creative Commons Attribution License, which permits unrestricted use, distribution, and reproduction in any medium, provided the original work is properly cited.

**How to cite this article:** Cuervo LG, Jaramillo C, Cuervo D *et al.* **Dynamic geographical accessibility assessments to improve health equity: protocol for a test case in Cali, Colombia [version 1; peer review: 2 approved]** F1000Research 2022, **11**:1394 https://doi.org/10.12688/f1000research.127294.1

**First published:** 28 Nov 2022, **11**:1394 https://doi.org/10.12688/f1000research.127294.1

## Introduction

Equitable accessibility is central to the United Nations Sustainable Development Goals and targets like universal health coverage and quality of care.[1–5] A common definition of accessibility is the relative ease (travel time by car) by which a destination (health service) can be reached from a given location (residence).[6–8] Equity assessments in geographical accessibility to health services typically rely on static metrics, such as distance or average travel times.[9–11]

Measuring equitable accessibility has several challenges. Accessibility studies assess distance or the shortest average travel time to the nearest facility; they seldom assess equity and are typically geared towards field experts. [12–14] These studies usually explore broad service categories without focusing on specific services people might need. They are lengthy, costly, and rarely address the dynamic temporospatial nature of accessibility, such as its links to traffic congestion. [6,15,16]

Reliable data on equity of accessibility to urban health services has been elusive for most cities due to limitations in sampling techniques, extrapolations, and the use of complex methods to capture temporospatial variations associated with traffic congestion. Stakeholders, including urban and health service planners, have relied on indirect fixed assessments that fail to address the impact of traffic congestion on equity.[6,9,15,17]

These challenges were understandable because assessments were cumbersome and required detailed origin-destination studies with small samples from home surveys, traffic corridor speed cameras, or extrapolations from average traffic in selected corridors, with limited intersectoral and multistakeholder participation.[6,16,18–26] Results would turn irrelevant given the rapid changes in conditions, including traffic congestion, populations, or infrastructure.[27]

Travel times affect geographic accessibility and the quality of care.[1,28,29] Poor accessibility can lead the most socially disadvantaged populations to pay the highest share to reach health services, an aberration of social justice known as the "inverse care law." Lengthy travel times hurt people; they are detrimental to health, well-being, and family finances.[28,30–35] Measuring travel times might reveal problems hiding in plain sight. Addressing accessibility might help people unable to choose a better place of residence overcome structural barriers to health.[36]

Travel time assessments have been widely available for commodities and commerce and powered consumer apps. These developments have yet to translate into a systematic integration of dynamic equity assessments into urban and health services planning or public sector debates about land use and how to put health services within reach of the broadest population possible.

Measurements have proven difficult, and this project explores a new approach to making measurements feasible, scalable, and adaptable to urban sprawl and lengthening journeys. This new line of research explores if market forces and land use plans achieve service accessibility and if this holds for populations in situations of vulnerability.[9,12,29]

## The need for this study

This proposal spurs the scaling up and replicating of accessibility analyses to health services in urban centers while promoting accessibility indicators based on dynamic travel times. The research demystifies the use of big data and analytics that reveal the needs of citizens, including the most vulnerable.[37–42] The project will lay the basis for subsequent studies that assess the value and use of dynamic accessibility and equity assessments in urban and health services planning.

This project explores a new approach to making such measurements feasible, scalable, and adaptable to urban sprawl and long journeys. This new line of research examines whether market forces and land use planning achieve services' accessibility and if this holds for populations in situations of vulnerability.[29,43]

Using reliable data that is systematically updated and publicly available could be a game-changer.[6,37,44,45] This study tests a new approach for assessing dynamic accessibility to health services, providing an equity perspective and using digital data sources. [9,44,46]

Using data readily available in the public domain allows these assessments to be completed in a shorter time and with a lower budget. When combined, the growing millions of measurements of travel times (big data) passively collected by mobile apps, the digitalized georeferenced sociodemographic data from the census, and the geolocation of health services, provide a dynamic assessment of accessibility that accounts for temporospatial variations related to traffic congestion.[6,46,47] Big data provides millions of measurements that allow identifying unexpected correlations with a level of detail and accuracy that surveys and inferences cannot match.[45]

This study aims to overcome the limitations of regular accessibility assessments by prioritizing dynamic travel times and adopting recommended knowledge production and use practices. The following section details some key features and good practices that contribute to addressing present challenges:

- **Multistakeholder engagement:** a diverse intersectoral team of stakeholders contributes to the AMORE Project throughout the research process, and their inputs also informed the AMORE Platform conceptualization and development.[48–51] Contributors to the AMORE Project Collaborative Group represent the government, community, health service providers, and end users (consumers) who may directly or indirectly shape decisions, policies, plans, and programs.[48,52–54,55]

- **Measurement** of dynamic travel times using "time to destination" is a universal and comparable metric used by urban dwellers and users of navigation and travel apps.[44]

- **Digitization and datafication** by using anonymized publicly available georeferenced data of housing, people, and services, including disaggregated sociodemographic characteristics.[6,27,37]

- **Using analytics and modelling** to obtain reasonable estimates and maintain efficiencies and affordability while still delivering valid and reliable forecasts.

- **Disaggregating sociodemographic data** to deliver an equity analysis of accessibility.[56–58]

- **Scalability and replicability** using sources increasingly available to low- medium, and high-income settings. The approach can be scaled, adapted, and replicated to other locations, transportation means, services, or sectors.

Subsequent research will explore if revealing territorial inequities in urban and health services planning could catalyze intersectoral responses.[30,59–63] Intersectoral collaborations rarely occur naturally and are challenged by the lack of consensus on issues and metrics. Using metrics and methods that all parties understand and facilitate direct communication could contribute to intersectoral action; assessing this will require additional research and is the subject of a separate proposal.[9,60,64–68]

## Objectives

### General objective

To assess dynamic accessibility assessments for selected healthcare services in urban Cali, Colombia, and predict the maximum improvements possible if new services were added.

### Specific objectives

- To assess the temporospatial characteristics of equity and accessibility to hemodialysis, radiation therapy (radiotherapy), and tertiary care emergency services when traveling by car in urban Cali, Colombia.

- To provide dynamic assessments based on selected (arbitrary) travel time thresholds.

- To assess if populations in a situation of vulnerability needing hemodialysis, radiotherapy, and tertiary care emergency services will likely incur longer journeys when traveling by car in urban Cali, Colombia.

- To identify common variations of dynamic accessibility at two moments of the COVID-19 pandemic from an equity perspective.

- Assess the magnitude of absolute and relative accessibility variations attributed to traffic congestion.

- To estimate potential improvement for accessibility gained by expanding services.

Box 1 provides a plain language summary of the project. The *Extended data* contains the goals of the AMORE project and previous versions of the protocol.[69,70,71]

**Box 1. Overview of the study.**

**What is already known on this topic** – dynamic travel times are not available for most cities, including Cali, and are not integrated into urban and health service planning; dynamic geospatial analyses reveal the effects of traffic congestion on health equity and accessibility to health services, a determinant of health.

**What this study adds** – it will provide estimates of accessibility with an equity perspective using simple methods and metrics that concerned stakeholders might find familiar. This will test if dynamic accessibility assessments can be done with existing data. The study adds an approach to analyzing dynamic geographic accessibility by tapping into hundreds of thousands or millions of observations to analyze, predict, improve geographic accessibility to health services, and identify new correlations.

**How this study might affect research, practice, or policy** – This study will provide a new metric and data source to address inequities aggravated by poor accessibility offering new perspectives on land use and the expansion of health services. The study prioritizes travel times over distance, accounting for the temporospatial variations caused by traffic congestion. Subsequent examinations can explore stakeholders' valuing of the data and their communication of the methods and findings with peers and counterparts.

## Protocol

### Ethics

This health services quality improvement protocol will use anonymized coded secondary data sources from publicly available open records and will not include human subjects' research.

This cross-sectional study will use a research design of GIS modelling applied to case studies based on analyses of publicly available secondary data. This study will conduct paired cross-sectional assessments comparing equity in health services accessibility from the 6$^{th}$ to the 12$^{th}$ of July 2020 and from the 23$^{rd}$ to the 29$^{th}$ of November 2020.

Reporting of study results will follow the STROBE Guideline for cross-sectional observational studies and incorporate elements from other guidelines, such as those on equity assessments (CONSORT-E and PRISMA-E equity extensions), public health and policy interventions (TIDieR-PHP), reporting of analytical models (e.g., SPIRIT-AI extension), and multistakeholder engagement with patient and public participation in research (GRIPP2).[57,72–74]

### Context and study population

The study will be a proof-of-concept for implementation in Cali (estimated 2,258 million in 2020), the third largest and most populous city in Colombia and the dominant urban center of Colombia's southwest and pacific regions (approx. 564 km$^2$). The study includes the entire urban population. Nearly half of Cali's population lives in low-income housing, 41% in medium income, and 9% in high-income housing. About 84% of the population identifies as white descent, and 14% identify as afro-descendent, with a small proportion identifying as Indigenous or Rrom.[78–80]

The COVID-19 pandemic severely impacted the local economy. By January 2021, unemployment rates in Cali rose to 23.2% for women and 14.6% for men, a one-year increase of 8.1% and 3.1%, respectively. The situation was worse for the youth, with an estimated 52% of women and 47.2% of men dependent on the informal economy. One in five people was unemployed, and unemployment rates were substantially higher for those living in lower socioeconomic areas. Cali absorbed 139,000 migrants from Venezuela over the past five years, with more than 25,000 in 2020.[79,80]

For the reports, contextual data will be obtained to provide an overview of the use and demand of services. Sources include reports and platforms such as the "Cuentas de Alto Costo."[82–84]

In preparatory dialogues with contributors, we learned about plans to transform Cali into a Special District with its twenty-two communes converted into six to eight minor districts to be led by minor district mayors.[85] This new political and administrative layout might raise interest in this topic as new authorities might want to discuss accessibility and equity issues with their constituents and in power-brokering negotiations, noting the equity implications of the concentration of health services in a few sectors of the city.

### Data sources

The study will use anonymized, aggregated data from the following data sources:

- Microdata of Colombia's National Census for Cali 2018 will be downloaded from the official public website of the National Department of Statistics– DANE.[86] This will provide sociodemographic data of the populations at

the block level for the entire city. The census population had a 28.1% adjustment estimated for 2020 to account for intercensal growth, under-registration, and migration.[78,79,81,87]

- The city's transportation analysis areas (TAZ) and census administrative sectorization for urban Cali will be obtained from the IDESC portal.[88] This data allows linking TAZs with city blocks. TAZs are adequate to estimate travel times and less detailed than blocks, thus reducing the number of travel time measurements and adding anonymity to the population.

- Approved health services relevant to the chosen scenarios, will be obtained from the National Special Registry of health services providers – REPS from the Ministry of Health and Social Protection. The services geolocation will be verified with Google Maps. Approved services were checked in June and October 2020 and January 2021, finding they remained unchanged. This protocol will assess accessibility to the entire city's fourteen tertiary care hospitals with emergency services (REPS Code "Alta complejidad" + 501); eleven hemodialysis units totaling 370 chairs (REPS code 733); five radiotherapy services (REPS code 711).

- Google's Distance Matrix API provides big data measurements of travel times from the origin (TAZ for the residence) to the destination (TAZ of the health service). It allows the identification of travel time changes during the assessed weeks.

## Data integration: The AMORE Platform

Secondary data will be integrated into the AMORE Platform, a web-based digital platform developed with inputs and feedback from stakeholders and piloted by the AMORE Project.[89–91] The Platform is hosted by IQuartil SAS. See https://www.iquartil.net/proyectoAMORE.

The AMORE Platform was developed and tested following a design-thinking approach between June and August 2020.[49,92–95] The final version was completed in February 2022. The digital web-based platform was developed for this project by the principal investigator with input from experts in data science, public health, logistics, and mobility and a wide range of stakeholders (A description of the development and piloting phases of the AMORE Platform can be provided).

Figure 1, Figure 2, and Figure 3 display examples of the AMORE Platform's interface or "front-end" panels (presentation layers) with its zoomable choropleth maps and graphics that integrate multiple layers of data. Filters activated by tapping on the graphs act on sociodemographic variables, travel times, and health services to offer a descriptive analysis. The front-end has been developed with Microsoft's Power BI™ (v. 2.102.683.0). The back end (data access layer) is written in Python (v. 3.9.10)™ open-source software from the Python Software Foundation – PSF and in the Konstanz Information Miner – KNIME (v 4.5.1), a free and open-source data analytics, reporting, and integration platform.

## Study variables

Reports will describe the people and percentages of the entire population able to reach services within a set threshold with peak and free-flow traffic conditions for each scenario. The variations in these accessibility figures will be disaggregated

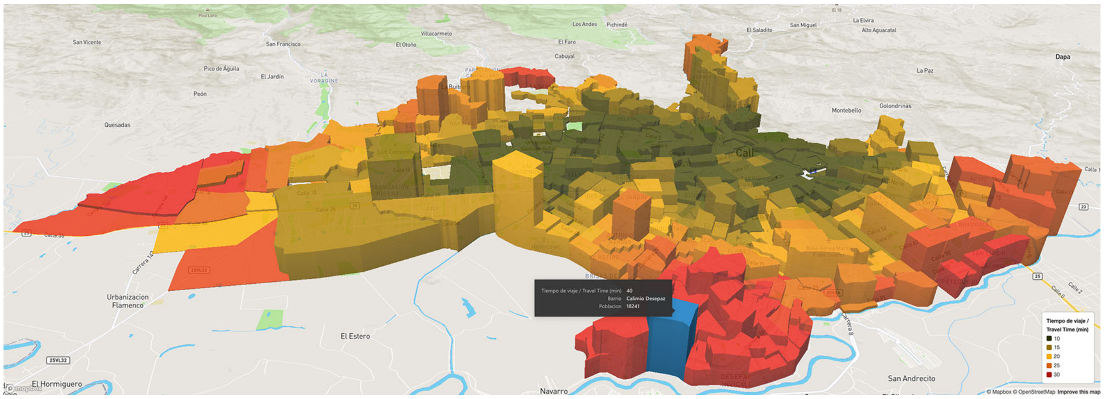


**Figure 1. Cali, accessibility to tertiary care emergency service late morning to early afternoon Mon-Sat.** North to the right and west at the top. Source: AMORE Platform. ©Mapbox © OpenStreetMap

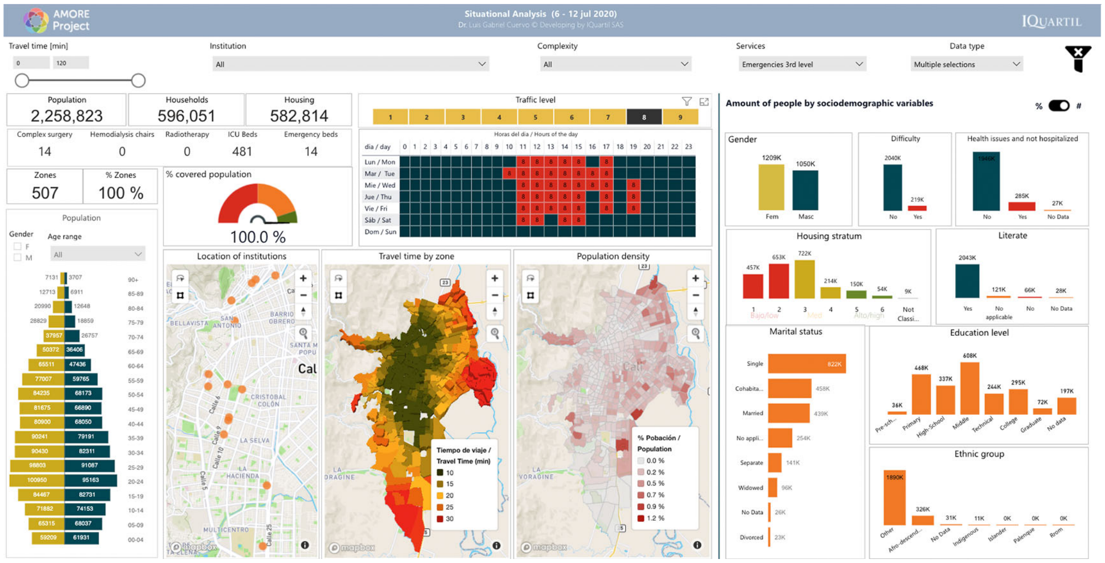


Figure 2. **AMORE Platform Interface for situational analysis for tertiary care emergencies.** Source: AMORE Platform. ©Mapbox

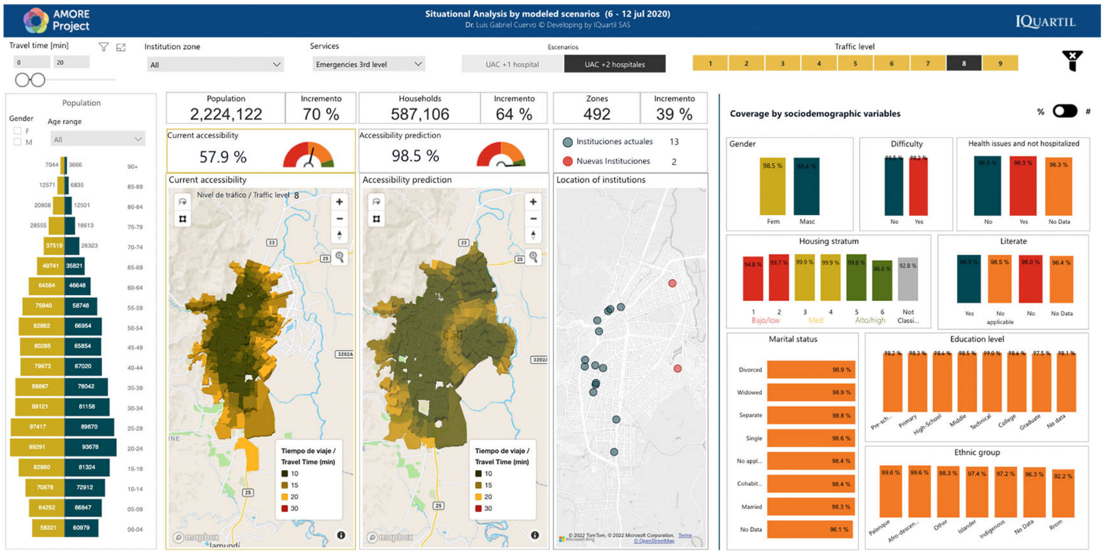


Figure 3. **AMORE Platform with predictive analyses of adding two new tertiary care emergency services.** Source: AMORE Platform. ©Mapbox

by sociodemographic characteristics such as sex, ethnicity, the socioeconomic stratum of housing, maximum education attainment, and marital status. These reports will contrast the statistics for peak and free-flow traffic conditions. These reports will use an arbitrary 15-minute threshold for accessibility by car to tertiary care emergencies and 20-minutes for hemodialysis and radiotherapy. Colombia's census provides a binary sex classification (male-female) based on self-reporting.[86,96]

Graphs will be used to present variations in accessibility as traffic congestion increases for different travel time intervals (e.g., 10-minute intervals vs. accessibility for each socioeconomic stratum), as shown in Figure 4. The contrast will be drawn between results obtained for July and November 2020. Reports will include the location(s) maximizing accessibility if one or two new services are added, contrasting predicted accessibility vs. measured accessibility for July and November 2020, and the recommended services locations. For an example, see Figure 3. All estimates in this test

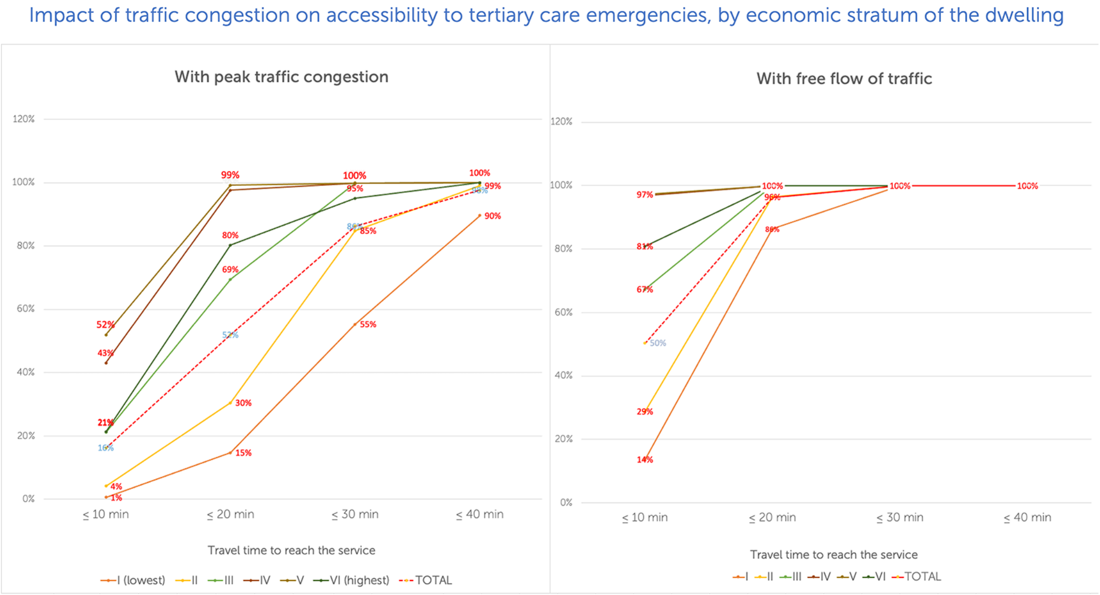


**Figure 4. Comparing accessibility by socioeconomic stratum, tertiary care emergencies.**[97]

case use travel by car. Reports will include visualizations from the AMORE Platform, tables, and simple graphs with descriptive statistics.

## Data analysis

Data and images for analyses will be obtained from the AMORE Platform and presented using descriptive statistics for absolute figures (people) and relative (percentage of population), as shown in Figure 2 and Figure 3.

The use of relatable and commonly used metrics (i.e., time to destination), descriptive statistics (percentage of a population that can reach the services within a travel time threshold), visualizations, and simple graphs (e.g., Figure 2, Figure 3, Figure 4) and maps (e.g., Figure 1) are chosen to allow non-specialized stakeholders to understand the effects of accessibility on populations and health equity.

The study will deliver (1) situational analyses of accessibility to a selection of urgent or frequent health care services scenarios in urban Cali, Colombia, and (2) predictions of potential improvements to the accessibility of adding services under changing assumptions.

Arbitrary travel-time points will be used (15 minutes for urgent care; 20 minutes for frequent care services); we found no standards for travel time thresholds.

The situational analyses of accessibility will consider the following study scenarios:

1. **Urgent care,** assessing travel times to the health emergency department with the shortest journey by car, among the 14 hospitals with tertiary care emergency departments.

2. **Frequent care,** assessing travel times to ambulatory services that require regular use. The analyses will investigate the shortest journey to five radiation therapy services and eleven hemodialysis units.

For these case studies, the project will deliver a:

- **Situational analysis** uses descriptive and diagnostic analytics to assess accessibility under traffic conditions. Choropleth maps mark the boundaries of travel times Figure 1. The blocks building the map represent Traffic Analysis Zones (TAZ), and their height in the 3D choropleth map represents population density. By pointing to a TAZ, identification, population, and travel time to the nearest facility are displayed, and the sociodemographic characteristics of the people are presented in a dashboard (Figure 2). The dashboard also includes a choropleth map with the density of the population being analyzed.

**Optimization analysis** using predictive and prescriptive analytics for modelling. Using heuristic techniques, the research will predict accessibility with different service schedules or when adding up to two new services in locations that maximize accessibility. Case studies will focus on hemodialysis and radiotherapy, which are used to treat high-cost conditions and must be accessed repeatedly for prolonged periods. For example, hemodialysis usually requires 3-5 weekly sessions, and radiotherapy may require daily sessions for weeks. Predicted accessibility with new services will be compared with measured accessibility (Figure 3).

## Preparation for the project

Beginning on 12th June 12 2020, interviews were conducted with key informants, local authorities, and experts to inform the project, assess feasibility, and develop a protocol. They provided verbal consent to participate. These interviews offered contextual insights and ideas for the AMORE Platform to address the needs of data users and stakeholders. Discussions helped identify contributors and provided valuable contextual information. They also revealed how stakeholders approached health equity and accessibility and the relationship to urban and health services planning and land use. Stakeholders offered insights into the elements and processes of urban and health services planning, public policy, and advocacy.

Interviewees included:

- Members of the SIGELO Project on vulnerability, accessibility, and logistics in the context of the COVID-19 pandemic, Universidad del Valle
- Health equity and public health experts at the Bruyère Institute, University of Ottawa
- Contributors to Cali's Administrative Department of Municipal Planning (DAPM)
- Advisors and staff working with Cali's local government, including the secretariats of Health, Mobility, Urban planning, and Emergency response and preparedness.
- Data Science for All Team33 and IQuartil SAS analysts.
- Former local government and education authorities
- Urban observatories, urbanists, and networks on urbanism, mobility, and public health.
- Innovators and advisors working with health services and systems
- Service providers, including managers and science advisors
- Health services and accessibility data users
- Doctoral and professional networks
- Designers, graphic and science communications professionals, and artists
- Researchers and research sponsors.

Overall, 28 meetings were held with key informants and stakeholders between July 2020 and March 2022, when the advanced prototype of the AMORE Platform was completed. Like every doctoral thesis, this project is subject of yearly follow-up reviews by the Commission of the Doctoral Program on Research Methodology for Biomedical Research and Public Health of the Universitat Autònoma de Barcelona, since October 2020.

During the preparatory phase, the project was also debated in international fora such as the Global Health Learning Network of the University of Ottawa, The 4th Urban Forum "Lima Cómo Vamos," the CEDEUS-REDEUSLAC II International Symposium of Doctoral Candidates on Urban Development and Sustainability for Latin America, and the Caribbean (Chile), and with urban observatories (Cali, Bucaramanga).[89,98,99]

These interviews shaped the AMORE project and platform. The objectives and platform were discussed from a theoretical perspective. Once the prototypes of the AMORE Platform became available, tests and demonstrations were made as part of the validation.

Data downloads and sources were stored in a dedicated repository using CSV formats to adhere to data sharing and reuse good practices. They will be made public with the publication of relevant research reports. Similarly, the AMORE Platform hosted by IQuartil SAS is made accessible with the completion of non-disclosure agreements. We expect to make platform sections publicly available as relevant results are published.[100]

## Fidelity/adaptation

The fidelity of the AMORE Platform is based on data validation and verification exercises, comparing the findings from the AMORE Platform using the two data downloads and the two development teams.

The results of the platform will likely be optimistic for several reasons. For example, people do not always travel to the service with the shortest journey for known (e.g., lack of coverage from the insurance in the institution) and unexpected reasons (familiarity, poor navigation aids, reputation). The potential for improved accessibility would be accurate if all people were entitled to access those services.

The census includes respondent-reported data that is subject to interpretation. For example, variables like disability and health status are self-reported, and the question is unspecific.

Respondents may not find a suitable response option. For example, ethnicity has no category for Caucasian or mestizo populations representing a substantial part of the population. People with mixed backgrounds may find no suitable option to represent them.

The census is still well suited for this study: the data has been digitized, and sociodemographic data are linked with the residential block. The place of residence is a common starting point for people undergoing hemodialysis or radiotherapy, children, the elderly, and those not engaged in formal employment.

Mobile phone data is impractical because it cannot be accurately tied to reliable sociodemographic data; coverage varies among the population and excludes those unregistered as users or without a phone. It also has technical limitations.[101]

Accessibility and spatial equity have been studied in Cali by the Research Group on Transport, Transit and Roads (GITTV) of the Faculty of Engineering of the Universidad del Valle. Members of this group contributed to the validation of the AMORE Platform, and the preliminary findings of the AMORE Platform are consistent with those of the GITTV and other authors.[102–107]

## Harms, risks, and ethical considerations

This observational study addresses the impact of mobility on health equity without researching human subjects and by integrating anonymized coded secondary data obtained from openly available records.

The study does not involve human subjects' research. The AMORE Platform and dynamic geospatial analyses expose social justice issues and potential solutions of benefit to society by enabling informed decisions relevant to policies, plans, and procedures for improving health equity. This data can also predict or monitor changes in urban accessibility. The data used is anonymized and publicly available. Under Colombian law, this component fits the definition of research without risk, as described in Resolution 008430-1993 of the Ministry of Health.[108] This was corroborated on 25th July 2022, by the Research Ethics Committee of the School of Engineering of the Universidad del Valle, which declared the project "without risk" per Colombian law (Ref: CEIFI 010-2022). The project was cleared on September 16, 2022 by the Commission on Ethics in Animal and Human Experimentation (CEEAH), and the Vice-Rector for Research, Universitat Autònoma de Barcelona (Ref: CEEAH-6100) on September 20, 2022.

The study can challenge current thinking with data and disrupt traditional approaches to land use and health services planning that may perpetuate pervasive inequalities that could fuel social strife and corruption.[102–105,109–115]

The ethical approach of this study follows the broader principles and considerations of public health ethics and health systems ethics; it generates population data valuable to address inequity and social injustice, is helpful for accountability and is relevant to intersectoral action.[116,117] The need for further guidance on these issues remains a challenge for cross-sectoral collaboration. It is part of the ongoing discussions on stakeholder engagement in global health.[73,117]

Accessibility is a determinant of health on the supply side of health equity.[119] Useful, valid equity assessments in accessibility matter to health systems and social justice. Having action-oriented data to challenge established thinking might contribute to various SDGs, such as improving good health and well-being (SDG 3), reducing inequalities (SDG10), having sustainable cities and communities (SDG11), improving infrastructure (SDG 9), and facilitating partnerships to achieve the SDGs (SDG17).[3,48,52,60,116]

These ideals are synergic with other urban development and planning initiatives on a human scale: Smart City, the 15-minute City, the Caring City, and the Committed City. Having data is the first step for technology to serve the needs of urban dwellers and inform public policy regularly or when facing health emergencies and pervasive inequities.[52,120–122]

The risks of this study are especially those associated with data science and artificial intelligence. Travel time data providers do not disclose the algorithms they use. These are empirically known to be accurate and are expected to be more accurate for the areas most travelled by people with network-engaged smartphones and sites where infrastructure and conditions remain stable; accuracy may vary across the city.[27,123,124]

There is a risk of errors in programming or labelling data; to control this risk, the validity of the data was tested, repeatedly reviewed, and found sensible by experts and local contributors. The chance that inaccuracies result from clustering traffic and times is low and is unlikely to change the overall picture the project analyzes.

The project reveals accessibility levels for populations and sectors of the city. It uses heuristic analysis to identify areas in which new services would significantly impact accessibility. These areas, like traffic conditions, may evolve. However, the data provided to inform decisions gives an overall idea of the locations that would optimize accessibility. It is unlikely that conditions and populations would change fast enough to make those broad estimations suddenly irrelevant. Regular updating of the AMORE Platform would allow for assessing these variations and would be the subject of further studies after this test case. Additional factors influence the use of a service, including insurance coverage entitlements. Exploring this would require different data layers and funding that exceeds the purpose and scope of the test case and would be a matter of subsequent implementation. There is an inherent risk of revealing social injustices or inequities that can lead to discomfort, alienation, or corrective action.

### Dissemination, promotion, and implementation of findings

As part of the project, communication tools such as animations, infographics, videos, summaries, and logos were developed.

The research team seeks to publish its reports in open-access impactful journals and present them to diverse audiences, including observatories, networks, and intersectoral groups.

The planned reports include:

- Accessibility of health services for the urban population of Cali, 2020: urgent care and frequent care

- Predicted accessibility of health services for the urban population of Cali with the addition of health services in areas that would maximize accessibility (urgent care and frequent care scenarios) or changes in service schedules (frequent care)

- Editorials and methodological articles.

### Protocol study status

Data extraction for research reports was initiated in January 2022 and is underway.

## Data availability

### Source data

The Data Sources section provides a list of publicly available data sources that will be used in this project. Relevant interfaces for each case study will be published with research results at https://www.iquartil.net/proyectoAMORE/.

### Extended data

Figshare: Theory of Change for AMORE Project Protocol 2022 https://doi.org/10.6084/m9.figshare.20485404.v2.[69]
This project contains the following extended data:

- The 2022 goals of the AMORE project and how they will be achieved

Open Science Framework: Dynamic geographical accessibility assessments to improve health equity: protocol for a test case in Cali, Colombia. https://doi.org/10.17605/OSF.IO/ETPMA.[70]

This project contains the following extended data:

- 20220726 Protocol 5.5 AMORE Project_Approved CEII-UdelV Assembled.pdf

- 20220726 Protocol 5.6.1 AMORE Project_Approved CEIIFI - CEEAH.pdf

Data are available under the terms of the Creative Commons Attribution 4.0 International license (CC-BY 4.0).

## Acknowledgments

The following people contributed to the brainstorming preceding the writing of the protocol and approved this acknowledgment: Peter Tugwell and Vivian Welch (Campbell Collaboration and Cochrane Equity Group); Myriam Rosero and María Fernanda Tobar Blandón (Universidad del Valle); Alberto Concha-Eastman (Senior Advisor to the Secretary of Public Health of Cali); David Paredes Zapata (Hospital Clinic, Transplant and Organ Donation Section, Barcelona, Spain); Fredy Enrique Ágredo Lemus (Ph.D. student in Health, Universidad del Valle and advisor to the Secretary of Health of Cali); Crhistian García (Grupo de Aseguramiento y Desarrollo de Servicios e la Secretaría de Salud de Cali); Fernando R. Martínez A. (Departamento Administrativo de Planeación Municipal); and María Fernanda Merino. As a tutor for the Doctoral program at the Universitat Autònoma de Barcelona, Dr Xavier Bonfill I Cosp has provided guidance with the academic program and reports. We are grateful to contributors working for the Escuela de Escuela de Salud Pública and the Departamento de Administración y Organizaciones of the Universidad del Valle, the Secretaría de Movilidad, and the Centro Regulador de Urgencias y Emergencias del Valle.

We thank artist Adriana Cabal Aulestia for authorizing Cali-themed paintings to illustrate the presentations and graphic designers Ingrid Faber and Carlos A. Faber for preparing the infographics. Ingrid Faber was commissioned to develop the logo and animation.

We acknowledge the contributions of the Team33/DS4A members to developing the prototype of the AMORE Platform: Catherine E. Cabrera, Daniel Cuervo, Darío Mogollón, Juan P. Morales, Santiago A. Tovar, Stephanie A. Rojas, Juan G. Betancourt, Rafael E. Ropero.

Stephen Volante and Cristina Cuervo provided editing assistance for early versions of the protocol.

An earlier version of this article can be found on SSRN (doi: http://dx.doi.org/10.2139/ssrn.4175407).

## References

1. Frenk J: **The concept and measurement of accessibility**. White K, editor. *Health Services Research: an anthology.* Washington DC: Pan American Health Organization/World Health Organization; 1992; p. 8–42855.
Reference Source

2. United Nations: *About the Sustainable Development Goals.* United Nations Sustainable Development; [cited 2020 Aug 22].
Reference Source

3. 53rd Directing Council: *66th Session of the Regional Committee of WHO for the Americas. Strategy for Universal Access to Health and Universal Health Coverage.*Pan American Health Organization/World Health Organization; 2014.
Reference Source

4. 57th Directing Council of the Pan American Health Organization: *71st Session of the Regional Committee of WHO for the Americas. Strategy and Plan of Action to Improve Quality of Care in Health Service Delivery 2020-2025/Estrategia y plan de acción para mejorar la calidad de la atención en la prestación de servicios de salud 2020-2025.* PAHO/WHO; 2019.
Reference Source

5. Commission on Social Determinants of Health: **Closing the gap in a generation: health equity through action on the social determinants of health: final report of the commission on social determinants of health. Combler Fossé En Une Génér Instaur Léquité En Santé En Agissant Sur Déterm Sociaux Santé Rapp Final Comm Déterm Sociaux Santé.** 2008 [cited 2022 Mar 19]; 247.
Reference Source

6. Moya-Gómez B, Salas-Olmedo MH, García-Palomares JC, *et al.*: **Dynamic Accessibility using Big Data: The Role of the Changing Conditions of Network Congestion and Destination Attractiveness.** *Netw. Spat. Econ.* 2018 Jun 1 [cited 2021 Jan 23]; **18**(2): 273–290.
Publisher Full Text

7. Luo W, Wang F: **Measures of Spatial Accessibility to Health Care in a GIS Environment: Synthesis and a Case Study in the Chicago Region.** *Environ Plan B Plan Des.* 2003 Dec [cited 2020 May 10]; **30**(6):

865–884.
Publisher Full Text

8. Bureau of Transportation Statistics: *Transportation Statistics Annual Report1997: mobility and access.*US Department of Transportation; 1997; 331.
Reference Source

9. Cuervo LG, Martínez-Herrera E, Cuervo D, *et al.*: **Improving equity using dynamic geographic accessibility data for urban health services planning.** *Gac. Sanit.* [cited 2022 Jun 11]; **36**: 497–499. (ePublication ahead of print).
PubMed Abstract | Publisher Full Text | Reference Source

10. Neutens T: **Accessibility, equity and health care: review and research directions for transport geographers.** *J. Transp. Geogr.* 2015 Feb 1 [cited 2022 Jan 20]; **43**: 14–27.
Publisher Full Text | Reference Source

11. Kong X, Liu Y, Wang Y, *et al.*: **Investigating Public Facility Characteristics from a Spatial Interaction Perspective: A Case Study of Beijing Hospitals Using Taxi Data.** *ISPRS Int J Geo-Inf.* 2017 Feb [cited 2022 Jan 20]; **6**(2): 38.
Publisher Full Text | Reference Source

12. Higgins C, Palm M, DeJohn A, *et al.*: **Calculating place-based transit accessibility: Methods, tools and algorithmic dependence.** *J. Transp. Land Use.* 2022 Feb 1 [cited 2022 Feb 22]; **15**(1): 95–116.
Publisher Full Text | Reference Source

13. Carrasco-Escobar G, Manrique E, Tello-Lizarraga K, *et al.*: **Travel Time to Health Facilities as a Marker of Geographical Accessibility Across Heterogeneous Land Coverage in Peru.** *Front. Public Health.* 2020 [cited 2022 Apr 4]; **8**.
PubMed Abstract | Publisher Full Text

14. Buzai GD, Santana Juárez MV: **Condicionantes Socioespaciales de la Salud (CSS): bases y alcance conceptual.** *Annu Div Geogr.* 2018; **2018**(12): 15.
Reference Source

15. Malhotra S, White HThe Campbell Collaboration, *et al.*: **Evidence and gap map-studies of the effectiveness of transport sector intervention in low and middle-income countries.** *Centre for Excellence and Development Impact and Learning (CEDIL).* 2022 Jun [cited 2022 Jul 11].
Reference Source

16. Al-Taiar A, Clark A, Longenecker JC, *et al.*: **Physical accessibility and utilization of health services in Yemen.** *Int. J. Health Geogr.* 2010 Jul 21 [cited 2022 Jul 11]; **9**(1): 38.
PubMed Abstract | Publisher Full Text

17. Kajalić J, Čelar N, Stanković S: **Travel Time Estimation on Urban Street Segment.** *Promet - TrafficTransportation.* 2018 Feb 23 [cited 2022 Apr 20]; **30**(1): 115–120.
Publisher Full Text | Reference Source

18. Hernández Gene FJ, Garnica BR: **Accesibilidad física de la población a servicios de salud pública en San Pelayo y Cereté, Córdoba, Colombia, año 2015.** *Perspect Geográfica.* 2017 Dec 19 [cited 2020 May 31]; **22**(2).
Publisher Full Text | Reference Source

19. Guagliardo MF: **Spatial accessibility of primary care: concepts, methods and challenges.** *Int. J. Health Geogr.* 2004 Feb 26 [cited 2020 May 7]; **3**: 3.
PubMed Abstract | Publisher Full Text | Reference Source

20. Páez A, Scott DM, Morency C: **Measuring accessibility: positive and normative implementations of various accessibility indicators.** *J. Transp. Geogr.* 2012 Nov [cited 2020 Jul 9]; **25**: 141–153.
Publisher Full Text | Reference Source

21. Currie G: **Quantifying spatial gaps in public transport supply based on social needs.** *J. Transp. Geogr.* 2010 Jan 1 [cited 2020 Jun 11]; **18**(1): 31–41.
Publisher Full Text | Reference Source

22. Lovett A, Haynes R, Sünnenberg G, *et al.*: **Car travel time and accessibility by bus to general practitioner services: a study using patient registers and GIS.** *Soc Sci Med.* 2002 Jul 1 [cited 2020 May 20]; **55**(1): 97–111.
PubMed Abstract | Publisher Full Text | Reference Source

23. Rosero-Bixby L: **Spatial access to health care in Costa Rica and its equity: a GIS-based study.** *Soc Sci Med.* 2004 Apr 1 [cited 2020 May 20]; **58**(7): 1271–1284.
Publisher Full Text | Reference Source

24. Teach SJ, Guagliardo MF, Crain EF, *et al.*: **Spatial Accessibility of Primary Care Pediatric Services in an Urban Environment: Association With Asthma Management and Outcome.** *Pediatrics.* 2006 Apr 1 [cited 2020 May 7]; **117**(Supplement 2): S78–S85.
Publisher Full Text | Reference Source

25. AccessMod 5|Modelling physical accessibility to health care: accessmod. [cited 2021 May 3].
Reference Source

26. Banke-Thomas A, Wong KLM, Collins L, *et al.*: **An assessment of geographical access and factors influencing travel time to emergency obstetric care in the urban state of Lagos, Nigeria.** *Health Policy Plan.* 2021 Nov 1 [cited 2022 Jun 17]; **36**(9): 1384–1396.
PubMed Abstract | Publisher Full Text

27. Mayer-Schönberger V, Cukier K: *Big data: a revolution that will transform how we live, work, and think.* Boston, Mass: Houghton Mifflin Harcourt; 2013; 242 p. (An Eamon Dolan book).

28. Alam K, Mahal A: **Economic impacts of health shocks on households in low and middle income countries: a review of the literature.** *Glob. Health.* 2014 Apr 3 [cited 2022 Jan 8]; **10**(1): 21.
PubMed Abstract | Publisher Full Text

29. Fullman N, Yearwood J, Abay SM, *et al.*: **Measuring performance on the Healthcare Access and Quality Index for 195 countries and territories and selected subnational locations: a systematic analysis from the Global Burden of Disease Study 2016.** *Lancet.* 2018 Jun 2 [cited 2020 Nov 11]; **391**(10136): 2236–2271.
PubMed Abstract | Publisher Full Text | Reference Source

30. Hart JT: **The inverse care law.** *Lancet.* 1971 Feb 27 [cited 2021 Mar 28]; **297**(7696): 405–412.
Publisher Full Text | Reference Source

31. Whitehead M: **The concepts and principles of equity and health.** *Health Promot. Int.* 1991 Sep 1 [cited 2021 Mar 28]; **6**(3): 217–228.
Publisher Full Text

32. Nambiar D, Mander H: **Inverse care and the role of the state: the health of the urban poor.** *Bull. World Health Organ.* 2017 Feb 1 [cited 2021 Mar 28]; **95**(2): 152–153.
PubMed Abstract | Publisher Full Text | Reference Source

33. Cookson R, Doran T, Asaria M, *et al.*: **The inverse care law re-examined: a global perspective.** *Lancet.* 2021 Feb [cited 2021 Mar 28]; **397**(10276): 828–838.
PubMed Abstract | Publisher Full Text | Reference Source

34. Fiscella K, Shin P: **The Inverse Care Law: Implications for Healthcare of Vulnerable Populations.** *J Ambulatory Care Manage.* 2005 Dec [cited 2021 Mar 28]; **28**(4): 304–312.
Publisher Full Text | Reference Source

35. OECD:*Health for Everyone?: Social Inequalities in Health and Health Systems.*OECD; 2019 [cited 2021 Feb 11]. (OECD Health Policy Studies).
Reference Source

36. Pineo H: *Healthy Urbanism: Designing and Planning Equitable, Sustainable and Inclusive Places.* London: Palgrave Macmillan; 2022.
Publisher Full Text

37. Ashford LS: **Demystifying Big Data for Demography and Global Health.** *Popul. Bull.* 2022; **76**(1): 34.
Reference Source

38. Acosta A: **Smart Cities & Inequidad: Forbes República Dominicana.** 2021 Jul 13 [cited 2021 Jul 17]; **82**(julio-agosto 2021): 12.
Reference Source

39. Boeing G, Higgs C, Liu S, *et al.*: **Using open data and open-source software to develop spatial indicators of urban design and transport features for achieving healthy and sustainable cities.** *Lancet Glob. Health.* 2022 Jun 1 [cited 2022 May 12]; **10**(6): e907–e918.
PubMed Abstract | Publisher Full Text | Reference Source

40. d'Obrenan H v d B, Huxley R: **Measuring what matters: supporting cities in tackling climate and health challenges.** *Lancet Glob. Health.* 2022 Jun 1 [cited 2022 May 12]; **10**(6): e788–e789.
PubMed Abstract | Publisher Full Text | Reference Source

41. Giles-Corti B, Moudon AV, Lowe M, *et al.*: **Creating healthy and sustainable cities: what gets measured, gets done.** *Lancet Glob. Health.* 2022 Jun 1 [cited 2022 May 12]; **10**(6): e782–e785.
PubMed Abstract | Publisher Full Text | Reference Source

42. Evidence Aid: **Factors affecting patients' ability to access healthcare: overview of systematic reviews.** *Evidence Aid. Evidence Aid.* 2022 [cited 2022 Jun 16].
Reference Source

43. Martin: *The Sustainable Development Agenda.* United Nations Sustainable Development; [cited 2020 Aug 22].
Reference Source

44. Śleszyń ski P, Olszewski P, Dybicz T, *et al.*: **The ideal isochrone: Assessing the efficiency of transport systems.** *Res. Transp. Bus. Manag.* 2022 Jan 19 [cited 2022 Jan 27]: 100779.
Publisher Full Text | Reference Source

45. Armstrong K: **Big data: a revolution that will transform how we live, work, and think.** *Inf. Commun. Soc.* 2014 Nov 26 [cited 2022 Apr 27]; **17**(10): 1300–1302.
Publisher Full Text

46. Jin T, Cheng L, Wang K, *et al.*: **Examining equity in accessibility to multi-tier healthcare services across different income households using estimated travel time.** *Transp. Policy.* 2022 Jun

1 [cited 2022 Apr 7]; **121**: 1–13.
Publisher Full Text | Reference Source

47. Bimpou K, Ferguson NS: **Dynamic accessibility: Incorporating day-to-day travel time reliability into accessibility measurement.** *J. Transp. Geogr.* 2020 Dec 1 [cited 2022 Apr 21]; **89**: 102892.
Publisher Full Text | Reference Source

48. Abimbola S: **Beyond positive a priori bias: reframing community engagement in LMICs.** *Health Promot. Int.* 2020 Jun 1 [cited 2021 Apr 3]; **35**(3): 598–609.
PubMed Abstract | Publisher Full Text

49. Abookire S, Plover C, Frasso R, *et al.*: **Health Design Thinking: An Innovative Approach in Public Health to Defining Problems and Finding Solutions.** *Front. Public Health.* 2020 [cited 2022 Jul 18]; **8**.
PubMed Abstract | Publisher Full Text

50. Bazzano AN, Martin J, Hicks E, *et al.*: **Human-centred design in global health: A scoping review of applications and contexts.** *PLoS One.* 2017 Nov 1 [cited 2022 Jul 18]; **12**(11): e0186744.
PubMed Abstract | Publisher Full Text

51. Brown T Design Thinking. 2008.

52. **Comprender para transformar, entrevista a Oriol Nel·lo.** 2018 [cited 2020 Aug 17].
Reference Source

53. Jull J, Giles A, Graham ID: **Community-based participatory research and integrated knowledge translation: advancing the co-creation of knowledge.** *Implement. Sci.* 2017 Dec 19 [cited 2021 Mar 25]; **12**(1): 150.
PubMed Abstract | Publisher Full Text

54. Papa E, Coppola P, Angiello G, *et al.*: **The learning process of accessibility instrument developers: Testing the tools in planning practice.** *Transp Res Part Policy Pract.* 2017 Oct [cited 2021 Jan 23]; **104**: 108–120.
Publisher Full Text | Reference Source

55. Cyril S, Smith BJ, Possamai-Inesedy A, *et al.*: **Exploring the role of community engagement in improving the health of disadvantaged populations: a systematic review.** *Glob. Health Action.* 2015 Dec 1 [cited 2021 Apr 2]; **8**(1): 29842.
PubMed Abstract | Publisher Full Text | Free Full Text

56. PROGRESS-Plus | Cochrane Equity: [cited 2020 Mar 23].
Reference Source

57. Jull J, Graham ID, Kristjansson E, *et al.*: **Taking an integrated knowledge translation approach in research to develop the CONSORT-Equity 2017 reporting guideline: an observational study.** *BMJ Open.* 2019 Jul 1; **9**(7): e026866.
PubMed Abstract | Publisher Full Text | Reference Source

58. Welch TF, Mishra S: **A measure of equity for public transit connectivity.** *J. Transp. Geogr.* 2013 Dec [cited 2021 Mar 4]; **33**: 29–41.
Publisher Full Text | Reference Source

59. Redman S, Greenhalgh T, Adedokun L, *et al.*: **Co-production of knowledge: the future.** *BMJ.* 2021 Feb 16 [cited 2021 Apr 23]; **372**: n434.
Publisher Full Text | Reference Source

60. Chircop A, Bassett R, Taylor E: **Evidence on how to practice intersectoral collaboration for health equity: a scoping review.** *Crit. Public Health.* 2015 Mar 15 [cited 2020 Oct 10]; **25**(2): 178–191.
Publisher Full Text

61. Organización Panamericana de la Salud, Organización Mundial de Salud:*Intersectorialidad y equidad en salud en América Latina: una aproximación analítica.* Washington, D.C.: 2015; 34.
Reference Source

62. Geurs KT, van Wee B: **Accessibility evaluation of land-use and transport strategies: review and research directions.** *J. Transp. Geogr.* 2004 Jun [cited 2020 May 10]; **12**(2): 127–140.
Publisher Full Text | Reference Source

63. United Nations: **Multi-stakeholder partnerships and voluntary commitments. Department of Economic and Social Affairs.** 2020 [cited 2020 Dec 12].
Reference Source

64. Whitty CJM: **What makes an academic paper useful for health policy?.** *BMC Med.* 2015 Dec 17 [cited 2022 Mar 24]; **13**(1): 301.
PubMed Abstract | Publisher Full Text

65. Hussain S, Javadi D, Andrey J, *et al.*: **Health intersectoralism in the Sustainable Development Goal era: from theory to practice.** *Glob. Health.* 2020 Feb 20 [cited 2021 Mar 28]; **16**(1): 15.
PubMed Abstract | Publisher Full Text

66. Organización Panamericana de la Salud: 2016 [cited 2020 Aug 11]; *Hoja de Ruta para el Plan de Acción sobre la Salud en Todas las Políticas.* OPS;
Reference Source

67. Public Health InstituteRudolph L, Caplan J, *et al.*: **Health in All Policies: Improving Health Through Intersectoral Collaboration.** *NAM Perspect.* 2013 Sep 18 [cited 2020 Sep 11]; **3**(9).
Reference Source

68. de Kadt E: **Making health policy management intersectoral: Issues of information analysis and use in less developed countries.** *Soc Sci Med.* 1989 Jan 1 [cited 2020 Oct 10]; **29**(4): 503–514.
PubMed Abstract | Publisher Full Text | Reference Source

69. Cuervo LG, Villamizar CJ, Jaramillo C, *et al.*: Theory of Change for AMORE Project Protocol 2022. figshare. figshare. [Dataset] 2022 [cited 2022 Aug 13].
Publisher Full Text

70. Cuervo L, Molina CJ, Cuervo D, *et al.*:Dynamic geographical accessibility assessments to improve health equity: protocol for a test case in Cali, Colombia. [Dataset]. 2022, November 11.
Publisher Full Text

71. Cuervo LG, Cuervo D, Hatcher-Roberts J, *et al.*: **AMORE Project: Integrated knowledge translation and geospatial analysis to improve travel times to health services (accessibility) and health equity in Cali, Colombia**: *a proof of concept using mixed-methods research* 2021.
Publisher Full Text

72. Petkovic J, Riddle A, Akl EA, *et al.*: **Protocol for the development of guidance for stakeholder engagement in health and healthcare guideline development and implementation.** *Syst. Rev.* 2020 Dec [cited 2021 Jan 3]; **9**(1): 21.
PubMed Abstract | Publisher Full Text

73. Pinnock H, Barwick M, Carpenter CR, *et al.*: **Standards for Reporting Implementation Studies (StaRI) Statement.** *BMJ.* 2017 Mar 6 [cited 2020 Jul 6]; **356**.
Reference Source

74. Campbell M, Katikireddi SV, Hoffmann T, *et al.*: **TIDieR-PHP: a reporting guideline for population health and policy interventions.** *BMJ.* 2018 May 16 [cited 2020 May 7]; **361**.
Publisher Full Text | Reference Source

75. Rivera SC, Liu X, Chan AW, *et al.*: **Guidelines for clinical trial protocols for interventions involving artificial intelligence: the SPIRIT-AI extension.** *Lancet Digit Health.* 2020 Oct 1 [cited 2020 Oct 30]; **2**(10): e549–e560.
PubMed Abstract | Publisher Full Text | Reference Source

76. Staniszewska S, Brett J, Simera I, *et al.*: **GRIPP2 reporting checklists: tools to improve reporting of patient and public involvement in research.** *BMJ.* 2017 Aug 2 [cited 2021 Mar 24]; **358**: j3453.
Reference Source

77. Kpokiri EE, Chen E, Li J, *et al.*: **Social Innovation For Health Research: Development of the SIFHR Checklist.** *PLoS Med.* 2021 Sep 13 [cited 2021 Sep 29]; **18**(9): e1003788.
PubMed Abstract | Publisher Full Text

78. DANE: **Información técnica y omisión censal 2018, aspectos conceptuales y metodológicos.** 2019 [cited 2020 Sep 10].
Reference Source

79. DANE: *Sistema Estadístico Nacional - SEN. Información del DANE para la toma de decisiones regionales: Cali - Valle del Cauca.* Colombia - Cali: DANE; 2021 Mar [cited 2021 Apr 24]; p. 223. (La información del DANE en la toma de decisiones de las ciudades capitales).
Reference Source

80. Dirección de Censos y Demografía - DCD:*Departamento Administrativo Nacional de Estadística - DANE. COLOMBIA - Censo Nacional de Población y Vivienda - CNPV - 2018.* Bogotá: Departamento Administrativo Nacional de Estadística - DANE; 2020 Feb; p. 124. (Microdatos). Report No.: DANE-DCD-CNPV-2018.
Reference Source

81. DANE: *Sistema Estadístico Nacional - SEN. Información del DANE para la toma de decisiones de las ciudades capitales: Cali - Valle del Cauca.* Colombia - Cali: DANE; 2020 Jan [cited 2021 Apr 24]; p. 79. (La información del DANE en la toma de decisiones de las ciudades capitales).
Reference Source

82. Gamboa O, Cotes M, Valdivieso J, *et al.*: **Estimation of the Need for Radiation Therapy Services According to the Incidence of Cancer in Colombia to 2035.** *Adv. Radiat. Oncol.* 2021 Nov 1 [cited 2022 Jul 14]; **6**(6): 100771.
Publisher Full Text | Reference Source

83. de Vries E, Buitrago G, Quitian H, *et al.*: **Access to cancer care in Colombia, a middle-income country with universal health coverage.** *J. Cancer Policy.* 2018 May 1 [cited 2022 Jul 14]; **15**: 104–112.
Publisher Full Text | Reference Source

84. Cuentas de Alto Costo - Fondo Colombiano de Enfermedades de Alto Costo:*HIGIA - Enfermedad Renal Crónica, Indicadores de Riesgo Demográfico. - Diálisis Adultos y Niños en Cali.*Cuenta de Alto Costo; 2022 [cited 2022 Jul 15].
Reference Source

85. Cali Distrito Especial tendría seis localidades: [cited 2021 Mar 31]. Reference Source

86. Censo Nacional de Población y Vivienda 2018: [cited 2020 Jul 23]. Reference Source

87. Grupo Interagencial sobre Flujos Migratorios Mixtos: **Página de GIFMM Colombia|R4V.** 2022 [cited 2022 Jul 20]. Reference Source

88. Unión Temporal UT SDG-CNC: **Encuesta de movilidad de hogares Cali 2015: Producto 3. Ámbito y zonificación. Steer Davies Gleave.** 2015 [cited 2020 May 30]. Reference Source

89. Proyecto AMORE: mejorando la equidad en la accesibilidad (tiempos de viaje) a los servicios de salud:*Prueba de concepto en Cali, Colombia. Sesión 01 Sala B: Ciudad Saludable, Smart Cities y Recursos Críticos.* Santiago, Chile: Centro de Desarrollo Urbano Sostenible - Chile; 2022 [cited 2022 May 27]. Reference Source

90. *AMORE Project - to improve health equity by reducing the travel time to essential health services.* Bethesda, MD, USA; 2021 [cited 2021 Sep 23]. Reference Source

91. DS4A Colombia 2020/Grand Finale: 2020 [cited 2020 Aug 16]. Reference Source

92. Hendricks S, Conrad N, Douglas TS, *et al.*: **A modified stakeholder participation assessment framework for design thinking in health innovation.** *Healthcare.* 2018 Sep 1 [cited 2021 Apr 2]; **6**(3): 191–196. PubMed Abstract | Publisher Full Text | Reference Source

93. Brown T, Wyatt J: **Design Thinking for Social Innovation.** *Dev Outreach.* 2012 Oct 3 [cited 2021 Apr 2]; **12**: 29–43. Publisher Full Text

94. Angotti T, Irazábal C: **Planning Latin American Cities: Dependencies and "Best Practices.".** *Lat. Am. Perspect.* 2017 Mar 1 [cited 2021 Apr 1]; **44**(2): 4–17. Publisher Full Text

95. Zurita I: **Revisiting Urban Planning in Latin America and the Caribbean.** 2009 [cited 2021 Apr 1]. Publisher Full Text

96. Heidari S, Babor TF, De Castro P, *et al.*: **Sex and Gender Equity in Research: rationale for the SAGER guidelines and recommended use.** *Res Integr Peer Rev.* 2016 May 3 [cited 2020 Mar 31]; **1**(1): 2. PubMed Abstract | Publisher Full Text

97. Cuervo LG, Martinez-Herrera E, Osorio L, *et al.*: **Dynamic accessibility by car to tertiary care emergency services in Cali, Colombia, in 2020: cross-sectional equity analyses using travel time big data from a Google API.** *BMJ Open.* 2022 Sep 1 [cited 2022 Sep 1]; **12**(9): e062178. Publisher Full Text | Reference Source

98. *Proyecto AMORE: Uso de Análisis Geoespacial para acceso a la Salud|4o Foro "Ciudades Cómo Vamos".* Lima: 2021 [cited 2022 Jul 16]. Reference Source

99. Global Health Learning Network- Seminar on Digital app technology to assess equity: Spatial analysis and equity for health services: Ottawa; 2022 [cited 2022 Jan 22]. vol. **4**. (Global Health Learning Network Seminar Series). Reference Source

100. Anderson JA, Eijkholt M, Illes J: **Ethical reproducibility: towards transparent reporting in biomedical research.** *Nat. Methods.* 2013 Sep [cited 2022 Jul 16]; **10**(9): 843–845. Publisher Full Text | Reference Source

101. Ayesha B, Jeewanthi B, Chitraranjan C, *et al.*: **User Localization Based on Call Detail Records.** *arXiv.* 2021 [cited 2022 Jul 20]. Reference Source

102. Jaramillo C, Lizárraga C, Grindlay AL: **Spatial disparity in transport social needs and public transport provision in Santiago de Cali (Colombia).** *J. Transp. Geogr.* 2012 Sep 1 [cited 2020 Jun 4]; **24**: 340–357. Publisher Full Text | Reference Source

103. Grindlay AL, Jaramillo C, Lizárraga C: *Spatial relationships between mobility opportunities and constraints of transport disadvantages: the case of Santiago de Cali, Colombia.* Rome, Italy: 2017 [cited 2021 Nov 1]; 119–129. Reference Source

104. Rodríguez Mariaca DA, Vivas Pachecho H, Pinzón MA, *et al.*: **Accessibility to the Employment Centers in Cali Through the Integrated System of Mass Transportation MIO.** *El Obs Reg.* 2017 [cited 2021 Nov 1]; (34): 1–7. Publisher Full Text

105. Wilches Astudillo CA, Jaramillo C, Murillo-Hoyos J: **Accesibilidad y equidad espacial al transporte público para pacientes con enfermedad neurodegenerativa en Santiago de Cali, Colombia.** *Investig Geográficas.* 2021 May 13 [cited 2021 Jun 4]. Publisher Full Text | Reference Source

106. Delmelle EC: **Casas I. Evaluating the spatial equity of bus rapid transit-based accessibility patterns in a developing country: The case of Cali, Colombia.** *Transp. Policy.* 2012 Mar 1 [cited 2020 May 19]; **20**: 36–46. Publisher Full Text | Reference Source

107. Scholl L: *Casos de estudio comparativos de tres proyectos de transporte urbano apoyados por el BID.*Banco Interamericano de Desarrollo; 2015 Jun [cited 2021 Nov 1]. Reference Source

108. Ministerio de Salud:*Resolución 8430 de 1993: Normas científicas, técnicas y administrativas para la investigación en salud.*República de Colombia; 1993. Reference Source

109. Whitty CJM: **What makes an academic paper useful for health policy?.** *BMC Med.* 2015 Dec 17 [cited 2022 Mar 24]; **13**(1): 301. PubMed Abstract | Publisher Full Text

110. Curtis C, Scheurer J: **Planning for sustainable accessibility: Developing tools to aid discussion and decision-making.** *Prog. Plan.* 2010 Aug 1 [cited 2020 Jul 6]; **74**(2): 53–106. Publisher Full Text | Reference Source

111. Observatorio de Sistemas de Ciudades: **Resultados Índice de Ciudades Modernas 2019.** 2021 [cited 2021 Jan 16]. Reference Source

112. **Cali emerges as epicentre of unrest in ongoing Colombia protests|Protests News|Al Jazeera.**[cited 2021 Oct 11]. Reference Source

113. *Colombia protests: UN "deeply alarmed" by bloodshed in Cali.*BBC News; 2021 May 4 [cited 2021 Oct 11]. Reference Source

114. Castro JES, González JDG: **The relationship between corruption and inequality in Colombia: empirical evidence using panel data for the period 2008-2017.** *Rev Iberoam Estud Desarro Iberoam J Dev Stud.* 2019 [cited 2021 Apr 26]; **8**(2): 28–43. Publisher Full Text | Reference Source

115. **Colombia Corruption Index|1995-2020 Data|2021-2023 Forecast|Historical|Chart.**[cited 2021 Apr 26]. Reference Source

116. Krubiner CB, Hyder AA: **A bioethical framework for health systems activity: a conceptual exploration applying 'systems thinking.'.** *Health Syst.* 2014 Jun 1 [cited 2021 Jan 1]; **3**(2): 124–135. Publisher Full Text

117. Cash R, Wikler D, Saxena A, *et al.*: *Casebook on ethical issues in international health research/edited by Richard Cash [… et al].*Estud Caso Sobre Ética Investig Int En Salud; 2009 [cited 2021 Oct 30]; vol. **209**. Reference Source

118. HEARD Project, USAID: **Establishing Principles of Stakeholder Engagement in Global Health Implementation Science and Research|HEARD.** [cited 2021 Jan 7]. Reference Source

119. McCollum R, Taegtmeyer M, Otiso L, *et al.*: **Healthcare equity analysis: applying the Tanahashi model of health service coverage to community health systems following devolution in Kenya.** *Int. J. Equity Health.* 2019 May 7 [cited 2021 Apr 23]; **18**(1): 65. PubMed Abstract | Publisher Full Text

120. Vida urbana y proximidad – Carlos Moreno: **Club de Lecturas.** [cited 2021 Jul 18]. Reference Source

121. BID I: **Ciudades inteligentes en América Latina.** *Conexión Intal.* 2018 [cited 2021 Jul 18]. Reference Source

122. **Observatorio de Sostenibilidad de Ciudades.**[cited 2020 Oct 12]. Reference Source

123. Luca M, Kleinberg J, Mullainathan S: **Algorithms Need Managers, Too.** *Harv. Bus. Rev.* 2016 Jan 1 [cited 2022 Jul 15]; Reference Source

124. Banke-Thomas, Aduragbemi, Kerry L. M. Wong, Francis Ifeanyi Ayomoh, Rokibat Olabisi Giwa-Ayedun, and Lenka Benova. "' **In Cities, It's NotFar, but It Takes Long': Comparing Estimated and Replicated Travel Times toReach Life-Saving Obstetric Care in Lagos, Nigeria.**" *BMJ Global Health* 6, no. 1 (January 1, 2021): e004318. Publisher Full Text

# Open Peer Review

## Current Peer Review Status: ✓ ✓

Version 1

Reviewer Report 17 July 2023

https://doi.org/10.5256/f1000research.139788.r175826

© **2023 Galvao L.** This is an open access peer review report distributed under the terms of the Creative Commons Attribution License, which permits unrestricted use, distribution, and reproduction in any medium, provided the original work is properly cited.

✓ **Luiz Galvao**
Oswaldo Cruz Foundation, Rio de Janeiro, Brazil

The paper proposes a new approach to assessing the accessibility of health services in urban areas. The approach uses dynamic travel times, which take into account traffic congestion, to assess the ease with which people can reach health services from their homes. The paper also takes an equity perspective, assessing how the accessibility of health services varies for different populations. It argues that the traditional approach to assessing accessibility, which uses static metrics such as distance or average travel time, is no longer adequate in the context of urban sprawl and traffic congestion. Dynamic travel times provide a more accurate measure of accessibility, as they take into account the real-world conditions that people face when trying to reach health services.

The paper uses data from Cali, Colombia, to show that people in vulnerable populations, such as those living in poverty or those with disabilities, often have less access to health services than people in more privileged populations.

The conclusions includes a new framework for assessing dynamic accessibility to health services. The framework takes into account the following factors:

- The location of health services
- The location of people's homes
- Traffic congestion
- The sociodemographic characteristics of the population

The paper argues that the framework can be used to identify areas where accessibility to health services is poor, and to target interventions to improve accessibility.

Overall, the paper is a valuable contribution to the literature on accessibility to health services. The new framework proposed by the authors has the potential to improve the way that accessibility to health services is assessed and improved.

**Is the rationale for, and objectives of, the study clearly described?**

Yes

**Is the study design appropriate for the research question?**
Yes

**Are sufficient details of the methods provided to allow replication by others?**
Yes

**Are the datasets clearly presented in a useable and accessible format?**
Yes

***Competing Interests:*** No competing interests were disclosed.

***Reviewer Expertise:*** environmental and global health

**I confirm that I have read this submission and believe that I have an appropriate level of expertise to confirm that it is of an acceptable scientific standard.**

Reviewer Report 30 March 2023

https://doi.org/10.5256/f1000research.139788.r164257

**© 2023 Stein A et al.** This is an open access peer review report distributed under the terms of the Creative Commons Attribution License, which permits unrestricted use, distribution, and reproduction in any medium, provided the original work is properly cited.

**Airton Tetelbom Stein**
[1] Graduate Program of Health Science, Public Health Department, Federal University of Health Science of Porto Alegre, Porto Alegre, Brazil
[2] Grupo Hospitalar Conceição, Porto Alegre, Brazil
**Rita Mattiello**
Post-Graduate Program in Epidemiology, Universidade Federal do Rio Grande do Sul, Porto Alegre, State of Rio Grande do Sul, Brazil

This protocol proposes an approach to assessing the place of residence as a spatial determinant of health in cities where traffic congestion might impact health services accessibility. The study provides dynamic travel times presenting data in ways that help shape decisions and spur action by diverse stakeholders and sectors.

This is a very adequate protocol to discuss a valid equity assessment in accessibility to health system and social justice.

In the Introduction section, it could have a broader view and suggest to include this article for rationale - Hosking J, Braubach M, Buss D, Khayesi M, Filho VP, de Sá TH. Towards a global framework for transport, health and health equity. Environ Int. 2022 Nov;169:107472. doi: 10.1016/j.envint.2022.107472. Epub 2022 Aug 17. PMID: 36116365.

There is a need to include in the introduction an article discussing the PROGRESS-Plus strategy, such as: O'Neill J, Tabish H, Welch V, Petticrew M, Pottie K, Clarke M, Evans T, Pardo Pardo J, Waters E, White H, Tugwell P. Applying an equity lens to interventions: using PROGRESS ensures consideration of socially stratifying factors to illuminate inequities in health. J Clin Epidemiol. 2014 Jan;67(1):56-64. doi: 10.1016/j.jclinepi.2013.08.005. Epub 2013 Nov 1. PMID: 24189091.

There is a need to discuss this report - "*Integrating health in urban and territorial planning: A sourcebook for urban leaders, health and planning professionals*" - https://unhabitat.org/integrating-health-in-urban-and-territorial-planning-a-sourcebook-for-urban-leaders-health-and, in which it describes the need to build habitable cities on a habitable planet: Processes to guide the development of human settlements – in this document referred to as “urban and territorial planning (UTP)”; and concern for human health, well-being and health equity at all levels – from local to global, and from human to planetary health. As there is a need for a closer relationships between public health and spatial planning.

Population ageing is one of the main demographic phenomena in Latin America and the Caribbean and in the world. This topic should also be addressed in this protocol, as this population has several disabilities due to chronic illness and inequity is something to manage based on this demographic transition.

**References**

1. Hosking J, Braubach M, Buss D, Khayesi M, et al.: Towards a global framework for transport, health and health equity.*Environ Int*. 2022; **169**: 107472 PubMed Abstract | Publisher Full Text
2. O'Neill J, Tabish H, Welch V, Petticrew M, et al.: Applying an equity lens to interventions: using PROGRESS ensures consideration of socially stratifying factors to illuminate inequities in health.*J Clin Epidemiol*. 2014; **67** (1): 56-64 PubMed Abstract | Publisher Full Text
3. Integrating health in urban and territorial planning: A sourcebook. *Geneva: UN-HABITAT and World Health Organization*. 2020. Reference Source


**Is the rationale for, and objectives of, the study clearly described?**

Yes

**Is the study design appropriate for the research question?**

Yes

**Are sufficient details of the methods provided to allow replication by others?**

Yes

**Are the datasets clearly presented in a useable and accessible format?**

Yes

***Competing Interests:*** No competing interests were disclosed.

***Reviewer Expertise:*** I am a family physician and epidemiologist. My main interest in research is health service research, primary health care and clinical guidelines.

**We confirm that we have read this submission and believe that we have an appropriate level of expertise to confirm that it is of an acceptable scientific standard.**

The benefits of publishing with F1000Research:

- Your article is published within days, with no editorial bias
- You can publish traditional articles, null/negative results, case reports, data notes and more
- The peer review process is transparent and collaborative
- Your article is indexed in PubMed after passing peer review
- Dedicated customer support at every stage

For pre-submission enquiries, contact research@f1000.com

F1000Research
