# Supplementary material for: Dynamic measurements of geographical accessibility considering traffic congestion using open data: a cross-sectional assessment for haemodialysis services in Cali, Colombia
Source: Lancet Reg Health Am. 2024 May 3;34:100752. doi: 10.1016/j.lana.2024.100752 (PMC11087994; doi:10.1016/j.lana.2024.100752)
Supplement: Authors and Contributors [file mmc5.pdf]

Dynamic measurements of geographical accessibility considering traffic congestion using open data: cross-sectional assessment for haemodialysis services in Cali, Colombia.

Authors

Abbreviation	AUTHOR'S SURNAME	AUTHOR'S Given name	ORCID	Conceptualization	Data curation	Formal Analysis	Funding acquisition	Investigation	Methodology	Project administration	Resources	Software	Supervision	Validation	Visualization	Wrote original draft	Draft review and editing	Approved the final draft for publication	Agrees on being accountable for all aspects	Signed
LGC	CUERVO	Luis Gabriel	0000-0003-2732-5019	Yes	Yes	Yes	Yes	Yes	Yes	Yes	Yes	Yes	Yes	Yes	Yes	Yes	Yes	Yes	Yes	2022-10-12
CJV	VILLAMIZAR JAIMES	Carmen Juliana	0000-0002-7031-8132		Yes					Yes			Yes			Yes	Yes	Yes	Yes	2022-12-12
LO	OSORIO	Lyda	0000-0002-5121-4741	Yes					Yes								Yes	Yes	Yes	2022-12-13
MBO	OSPINA	Maria B.	0000-0001-9305-7521														Yes	Yes	Yes	2022-12-11
DECD	CUERVO-DIAZ	Diana Elizabeth	0000-0003-3559-565X														Yes	Yes	Yes	2022-12-12
DC	CUERVO	Daniel	0000-0002-3323-9865	Yes		Yes			Yes		Yes	Yes		Yes	Yes		Yes	Yes	Yes	2022-12-12
MB	BULA	Maria	0000-0002-0611-0521					Yes									Yes	Yes	Yes	2022-12-12
PZ	ZAPATA	Pablo	0000-0002-6986-5382									Yes		Yes	Yes		Yes	Yes	Yes	2022-12-13
NJO	OWENS	Nancy J.	0000-0001-9136-7753														Yes	Yes	Yes	2022-12-12
JHR	HATCHER ROBERTS	Janet	0000-0002-1605-3911	Yes													Yes	Yes	Yes	2022-12-12
EAM	MARTIN P.	Edith A.	0000-0001-6080-2134														Yes	Yes	Yes	2022-12-13
FP	PIQUERO	Felipe	N.A. (Patient)														Yes	Yes	Yes	2022-12-13
LFP	PINILLA	Luis Fernando	0000-0001-6940-1097	Yes	Yes							Yes						Yes	Yes	2022-12-12
EMH	MARTÍNEZ HERRERA	Eliana	0000-0001-6524-4709					Yes					Yes		Yes		Yes	Yes	Yes	2022-12-13
CJ	JARAMILLO	Ciro	0000-0002-8820-2314	Yes	Yes	Yes		Yes	Yes				Yes	Yes			Yes	Yes	Yes	2022-12-12

Contributors

#	COLLABORATOR'S SURNAME	COLLABORATOR'S Given Name	Approved the final draft for publication	Agrees on being accountable for all aspects	Date signed	Government employee	Service provider or funder	Patient or next of kin to a patient	Advocate for accessibility	Career Researcher	Land-use, service planning	Expertise communicating science
1	AGREDO LEMOS	Freddy Enrique	Yes	Yes	2022-12-21							
2	AVILA RODRIGUEZ	German	Yes	Yes	2022-12-21		Yes			Yes	Yes	
3	CONCHA-EASTMAN	Alberto	Yes	Yes	2022-12-18	Yes				Yes	Yes	
4	FRANCO	Oscar H.	Yes	Yes	2022-12-15					Yes		
5	GARCIA ALTAMIRANO	Chrístian Camilo	Yes	Yes	2022-12-15	Yes					Yes	
6	MERINO JUAREZ	Maria Fernanda	Yes	Yes	2022-12-19	Yes					Yes	
7	MILLAN	Gynna	Yes	Yes	2022-12-19					Yes	Yes	
8	MURILLO-HOYOS	Jackeline	Yes	Yes	2022-12-19					Yes	Yes	Yes
9	PAREDES Z	Gabriel D	Yes	Yes	2022-12-20	Yes	Yes				Yes	
10	PAREDES-ZAPATA	David	Yes	Yes	2022-12-15				Yes	Yes		Yes
11	ROJAS R.	Oscar	Yes	Yes	2022-12-19	Yes	Yes					
12	TOBAR-BLANDÓN	María Fernanda	Yes	Yes	2022-12-15					Yes	Yes	
